# Communicating With Patients About Software for Enhancing Privacy in Secondary Database Research Involving Record Linkage: Delphi Study

**DOI:** 10.2196/20783

**Published:** 2020-12-15

**Authors:** Cason Schmit, Kobi V Ajayi, Alva O Ferdinand, Theodoros Giannouchos, Gurudev Ilangovan, W Benjamin Nowell, Hye-Chung Kum

**Affiliations:** 1 Population Informatics Lab Department of Health Policy & Management Texas A&M University School of Public Health College Station, TX United States; 2 Southwest Rural Health Research Center Department of Health Policy & Management Texas A&M University School of Public Health College Station, TX United States; 3 Pharmacotherapy Outcomes Research Center College of Pharmacy University of Utah Salt Lake City, UT United States; 4 Global Healthy Living Foundation Upper Nyack, NY United States

**Keywords:** Delphi technique, privacy, communication barriers, medical record linkage, research subjects, big data

## Abstract

**Background:**

There is substantial prior research on the perspectives of patients on the use of health information for research. Numerous communication barriers challenge transparency between researchers and data participants in secondary database research (eg, waiver of informed consent and knowledge gaps). Individual concerns and misconceptions challenge the trust in researchers among patients despite efforts to protect data. Technical software used to protect research data can further complicate the public's understanding of research. For example, MiNDFIRL (Minimum Necessary Disclosure For Interactive Record Linkage) is a prototype software that can be used to enhance the confidentiality of data sets by restricting disclosures of identifying information during the record linkage process. However, software, such as MiNDFIRL, which is used to protect data, must overcome the aforementioned communication barriers. One proposed solution is the creation of an interactive web-based frequently asked question (FAQ) template that can be adapted and used to communicate research issues to data subjects.

**Objective:**

This study aims to improve communication with patients and transparency about how complex software, such as MiNDFIRL, is used to enhance privacy in secondary database studies to maintain the public's trust in researchers.

**Methods:**

A Delphi technique with 3 rounds of the survey was used to develop the FAQ document to communicate privacy issues related to a generic secondary database study using the MiNDFIRL software. The Delphi panel consisted of 38 patients with chronic health conditions. We revised the FAQ between Delphi rounds and provided participants with a summary of the feedback. We adopted a conservative consensus threshold of less than 10% negative feedback per FAQ section.

**Results:**

We developed a consensus language for 21 of the 24 FAQ sections. Participant feedback demonstrated preference differences (eg, brevity vs comprehensiveness). We adapted the final FAQ into an interactive web-based format that 94% (31/33) of the participants found helpful or very helpful. The template FAQ and MiNDFIRL source code are available on GitHub. The results indicate the following patient communication considerations: patients have diverse and varied preferences; the tone is important but challenging; and patients want information on security, identifiers, and final disposition of information.

**Conclusions:**

The findings of this study provide insights into what research-related information is useful to patients and how researchers can communicate such information. These findings align with the current understanding of health literacy and its challenges. Communication is essential to transparency and ethical data use, yet it is exceedingly challenging. Developing FAQ template language to accompany a complex software may enable researchers to provide greater transparency when informed consent is not possible.

## Introduction

### Transparency and Trust in Secondary Database Research

The researcher-participant relationship rests on a fragile foundation, weakened by a history of scandal and abuse. Past research abuses might not be readily remembered by the general public, but the scars remain in the social subconscious [1-4]. Although many still view research positively, transparency between researchers and the study participants is a critical element in building and maintaining publics’ trust [5]. Transparency is key to supporting informed consent and *respect for persons*, the central ethical principle of the bioethical framework that governs human subjects research [6,7].

Unfortunately, it is difficult to achieve transparency in secondary database research because ethical review bodies (ie, institutional review boards [IRBs]) frequently waive informed consent requirements. This is done because informed consent is often impractical (ie, no contact between researchers and data subjects), and it is not possible to know the purpose of data use at all instances at the time of data collection [8]. Without the traditional informed consent process, secondary database researchers have trouble cultivating strong researcher-participant relationships.

The lack of informed consent is not without consequence. Individuals want to know how their data are used and want to be partners in the research process [5,9]. Moreover, people might be reluctant to participate in research when they fear that researchers are taking advantage of their information [9]. Survey evidence suggests that the public wants researchers to do a better job at communicating with the public [9,10]. A survey of 3516 patients suggests that communication about data protection methods would help improve comfort levels of the public with research [10]. This study also found that comfort levels of the public may be improved by using methods that minimize the exposure of unique identifiers while linking data for research purposes [10]. These findings suggest that a privacy statement that increases database research transparency and discusses the software used to enhance privacy would increase publics’ trust.

However, communication between researchers and the public can be challenging [9]. The expertise of researchers often obstructs effective communication. Jargon, technical language, and complex concepts are barriers to understanding. In secondary database research, these challenges are immense. Explanations of critical concepts often require additional explanations of related concepts. For example, a participant might be interested in knowing why a researcher needs a specific data element (eg, names); this might require further explanation of related issues such as record linkage, which invites additional questions and explanations of other concepts (eg, linked data) and additional tangential issues (eg, storage, maintenance, and data reuse).

Transparency in secondary database research requires communicating research risks, including privacy and confidentiality of personal data. Data subjects want to understand how researchers use and safeguard their data. This can include employing technology and software to enhance the privacy, confidentiality, and security of sensitive information. Thus, describing how technology is used to safeguard participant data will likely promote transparency and hopefully increase trust between researchers and data subjects.

For example, we have designed and evaluated a prototype user interface called MiNDFIRL (Minimum Necessary Disclosure For Interactive Record Linkage) to enhance data set confidentiality by restricting disclosures of identifying information while linking records. One study found that the on-demand interactive interface of MiNDFIRL reduced visible characters in the identifying data elements used in record linkage by 92.15% as compared with traditional manual record linkage, with little to no impact on decision quality or completion time [11,12]. In our 2 case studies with real data from 2 teaching hospitals, used in conjunction with automated record linkage software, we found that the prototype MiNDFIRL software could effectively support high-quality record linkage with much less data disclosure than manual record linkage [13]. Disclosing how a software, such as MiNDFIRL, protects research data can help cultivate publics’ trust in researchers.

Transparency on these issues is imperative as research data are particularly sensitive [9]. Secondary database researchers frequently use health information because of its importance in understanding critical societal issues, including effective treatments, health care costs, and service utilization. However, health data also carries a risk of social, economic, and psychological harm. Researchers often disclose the potential benefits and harms of the study to the study participants in the informed consent process. However, this is more difficult in secondary database research because of the lack of direct contact with data subjects.

### Improving Transparency in Secondary Database Research

In nonresearch settings, it is common to disclose privacy and confidentiality practices in a public privacy statement on the premise that transparency coupled with accountability is an effective form of privacy protection and ethical data use [14,15]. In theory, privacy statements provide an opportunity to increase public transparency. However, privacy statements in practice are long, technical, and burdensome to process and understand.

Frequently asked question (FAQ) documents are another mechanism used to promote transparency and understanding. In contrast to a privacy statement, which forces patients to find answers to their specific questions, FAQs present common questions and provide direct answers [16]. If a person only has a few questions they want answered, an FAQ can help them quickly find answers without reading volumes of irrelevant information. Some research institutions (eg, Texas A&M University) use a question and answer format, similar to an FAQ, in the informed consent templates for research (ie, “What are the risks of participating in this research?”).

This study aims to improve communication with patients about how a complex software, such as MiNDFIRL, is used to enhance privacy in secondary database studies to maintain publics’ trust in researchers. To do this, we aim to develop a template FAQ language that describes key issues in secondary database research to the public, including the necessity of sensitive identifiers (eg, names) in the record linkage process and the safeguards used by researchers to protect data from research risks (eg, researchers identifying their neighbor in a data set). To do this, we aim to develop a template FAQ language that describes key issues in secondary database research to the public, including the necessity of sensitive identifiers (eg, names) in the record linkage process and the safeguards used by researchers to protect data from research risks (eg, researchers identifying their neighbor in a data set). Through this template FAQ, we aim to improve research transparency, promote ethical data use, and cultivate trust between researchers and the public. This study focuses on the patient community and its perceptions of the benefits and risks of research using sensitive health data without informed consent.

We focus on health data because the public has heightened awareness of its sensitivity and harm [10,17]. However, many of the key concepts that we explore (eg, identifiability and record linkage) are broadly applicable to data obtained from nonhealth sectors (eg, education and social services) [18-21]. Our study primarily focuses on creating a template language for researchers using the MiNDFIRL software for record linkage, but we also anticipate that many FAQ items that we created will have broad applicability to secondary database research.

## Methods

### Design

We conducted a Delphi study using a web-based questionnaire administered through Qualtrics XM (SAP SE). The goal of the Delphi process was to create an FAQ to anticipate and answer the questions of the data subjects related to the use of their data in secondary database research. These questions include general questions and answers related to secondary database research, record linkage, and the MiNDFIRL software (ie, facilitating record linkage while enhancing confidentiality).

The Delphi technique is useful for studying communication between researchers and data subjects for several reasons. The Delphi approach is particularly suited to investigate communication strategies with patients and data subjects, where there are differences in thought, and the body of knowledge is still developing [22,23]. In addition, the Delphi process facilitates anonymous and confidential feedback from patients with diverse perspectives without the biases common to other consensus techniques such as group discussions and interviews [24,25]. Moreover, the structured feedback in a Delphi approach prevents dominant personalities from suppressing diverse inputs and perspectives [22].

During each Delphi round, we asked the patient expert panel a series of questions about a draft FAQ document. Between each round, we revised the FAQ language and content based on the feedback of the participants. In addition to written descriptions, the FAQ drafts included visual images and a short video demonstrating how the MiNDFIRL software works during the record linkage process (referred to as *patient matching* in the FAQ) to enhance user comprehension.

The FAQ drafts contained approximately 3000 words, and the accompanying survey instrument contained approximately 1500 words. Owing to the complexity and the word length of both documents, we excluded survey results if respondents completed the study in less than 10 min, to allow for the inclusion of constructive feedback. The study received ethical approval from the IRB of Texas A&M University (IRB2019-0234).

### Participants

We identified the chronic patient population (operationalized as patients with frequent encounters with the health care system) as the appropriate *expert* panel for the Delphi study. Patients with chronic conditions are the *experts* because they are likely to have conditions that are of interest to secondary database researchers, and patient voice is essential to health care, supporting systems (eg, health research), and related policy decisions [26]. In addition, patients with chronic conditions are likely to have multiple health care providers and thus require record linkage for a comprehensive understanding of their patient experience.

### Recruitment

We recruited patients via email using purposive sampling from 3 patient-powered research networks (PPRNs) and employees and staff of a large university in the south. Specifically, ArthritisPower, Chronic Obstructive Pulmonary Disease PPRN, and Interactive Autism Network were PPRNs that were part of the national Patient-Centered Clinical Research Network that we collaborated with. We included participants with at least one diagnosed chronic disease and with at least two physician visits for their condition in the previous 12 months. We compensated participants on a graduated basis: US $20 for completing round 1, US $30 for completing round 2, and US $50 for completing round 3, amounting to a maximum of US $100 in total as gift cards. We invited 45 participants to join this study.

### FAQ Development

Following recommendations from several health care organizations to enhance the readability level of the education material, the first FAQ had a Flesch-Kincaid readability score of 7.82 [27,28]. Findings from 3 prior studies informed the content and language of the FAQ template, including 2 nominal group technique (NGT) studies and a separate Delphi study [13,29]. One NGT study and a Delphi study used panels of experts in the ethical, legal, and social implications fields to help identify legal and ethical issues associated with secondary database research involving record linkage. Another NGT study used a panel of patients with chronic conditions to help identify issues that were important to the patient population.

### Delphi Rounds Procedure

Each Delphi round contained a mix of open-ended questions and 5-point Likert scale questions (eg, strongly agree, agree, neither agree nor disagree, disagree, or strongly disagree) administered on the web via Qualtrics. All 3 surveys were pilot tested by members of the research team before being administered. The round 1 survey included 50 questions, with fewer questions in successive rounds (Delphi survey instruments and materials are detailed in Multimedia Appendices 1-6). Participants were asked to provide feedback on the content of the FAQ document, including whether FAQ sections provided information that was understandable or important to patients. In addition, in round 1, we solicited open-ended responses after each FAQ section to identify gaps, solicit suggestions for new FAQ content (ie, “Questions 1-4 were about information and how it is used in research. Is there anything more that you would like to know on this topic?”), identify specific concerns (ie, “Do you have any concerns that you would like the FAQ to address”), and seek any other general comments. In rounds 2 and 3, the survey focused on the language and content of the FAQ that participants found problematic or where participant feedback suggested divergent opinions. In rounds 2 and 3, we also provided participants with a summary of the participant feedback from the previous round and a *tracked-changes* (eg, *redline*) version of the revised FAQs to enable them to see the specific revisions that were made based on the feedback. Participants were given just over a week to complete each round of the survey, with 2 reminder emails sent out within that time to reduce attrition.

#### Round Analysis, FAQ Draft Revisions, and Consensus Criteria

After each Delphi round, we triaged the FAQ content as *problematic or nonproblematic*. If FAQ sections had fewer than 3 participants providing negative feedback (ie, *disagree* or *strongly disagree*), they were deemed *nonproblematic*. We viewed nonproblematic FAQ sections as a consensus language and made only minor edits to these sections (eg, revisions to terminology). FAQ sections that received negative feedback from 3 or more participants (on any question relating to understandability or importance) were deemed problematic. For all FAQ items, we examined open-ended feedback and comments to identify points of confusion (eg, terminology, unexplained concepts, and poor phrasing), requested clarifications (eg, examples), and other participant recommendations. One researcher incorporated the feedback in a redlined document alongside relevant participant comments to ensure that the revisions addressed the feedback. This document was shared with the research team for review and approval. If we made substantial revisions to an FAQ section (ie, for FAQ items deemed to be problematic), we solicited additional feedback in subsequent rounds.

**Figure 1:**
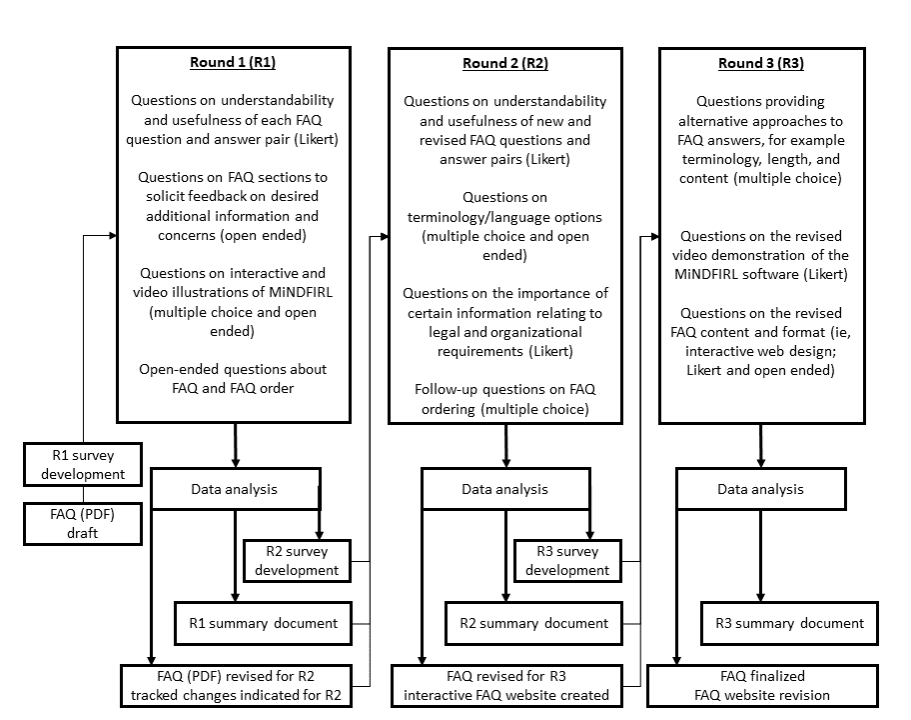
Overview of the Delphi process and content for round 1 (n=38), round 2 (n=37), and round 3 (n=33). FAQ: frequently asked question; MiNDFIRL: Minimum Necessary Disclosure For Interactive Record Linkage.

In addition to survey questions about specific FAQ question and answer content, rounds 2 and 3 contained questions to identify preferred terminology and strategies for communicating key concepts to the patient community. Whenever participant feedback suggested a divergence of opinions or suggestions, questions were devised to explore the divergence by explicitly raising the issues and providing participants with the opportunity to provide additional feedback (eg, providing alternative approaches to answering a question in the FAQ sections). An overview of our process is shown in Figure 1. The survey instruments and the round summaries provided to the participants are included in Multimedia Appendices 1-6.

We note that our consensus criterion, fewer than 3 individuals providing negative feedback, is highly conservative among Delphi studies. The Delphi technique does not demand a specific threshold for consensus [22,25,30]. Some Delphi studies use simple majorities to define consensus [25,31]. In contrast, our consensus criterion requires between 90% and 92% positive or neutral feedback, depending on the round, notably higher than other Delphi studies [25].

#### Thematic Analysis

After all 3 rounds, 2 researchers conducted a preliminary inductive thematic analysis of the open-ended responses to provide an additional context for our primary result: the FAQ template document. The thematic analysis was intended to identify broader lessons learned about communicating with patients about research from the Delphi process, including common concerns, desired content, and preferred communication approaches. One researcher reviewed the open-ended comments and generated an initial list of themes. The second researcher reviewed the initial list, all participant feedback, and made revisions to the list of themes. These draft themes were reviewed and discussed in a meeting with a third researcher providing input. We then identified participant quotes that were representative of the themes.

## Results

### Participant Demographics

A total of 45 participants were invited to participate in the Delphi study. Of those, 38 participated in round 1, resulting in a response rate of 84% (Table 1). Females (28/38, 74%) and non-Hispanic Whites (33/38, 87%) were disproportionately represented. The age range of the participants was 21 to 78 years, with a median of 49 years. The majority had a college degree or higher education (35/38, 92%), reflecting that the patients engaged in research with PPRNs and the staff at a university that we recruited from. Less than half of the sample (15/38, 39%) had 2 to 5 physician visits, with the rest having more than 5 visits in the last year and 63% (24/38) reported having good or very good health status. The most common clinical conditions included type II diabetes, thyroid disease, rheumatoid arthritis, and chronic pain.

**Table 1:** Demographic information of participants who completed round 1 (n=38).

Characteristics		Values
**Age (years)**		
	Mean (SD)	50 (14.6)
	Range	21-78
**Gender, n (%)**		
	Male	10 (26)
	Female	28 (74)
**Education, n (%)**		
	Some college credit and no degree	3 (8)
	Associate degree	7 (18)
	Bachelor’s degree	11 (29)
	Master’s degree	11 (29)
	Doctoral degree	6 (16)
**Race and ethnicity, n (%)**		
	White	33 (87)
	African American or Black	3 (8)
	Asian	1 (3)
	Other	1 (3)
**Average physician visits in 12 months, n (%)**		
	2-5 times	15 (39)
	6-10 times	14 (37)
	>10 times	9 (24)
**Self-reported health status, n (%)**		
	Excellent	2 (5)
	Very good	10 (26)
	Good	14 (37)
	Fair	12 (32)

### Principal Results

The principal result of this study is the final FAQ template document. The final FAQ template had a Flesch-Kincaid readability score of 8.66, which is slightly higher than the initial FAQ score of 7.82. The FAQ template is included in Multimedia Appendix 7, and it is also published on the web and accompanies the open-source software package for MiNDFIRL available on GitHub [32,33]. To the best of our knowledge, this is the first open-source software to include template documentation to facilitate transparent communication with subjects.

### Delphi Round 1

In round 1 (n=38), participants responded favorably to the overall FAQ, with 89.86% (2322/2584) of responses within the strongly agree and agree category across all survey questions on the FAQ content. Most participants strongly agreed or agreed that the questions included in the FAQ were easy to understand (755/798, 94.6%), the answers provided in the FAQ were easy to understand (407/494, 82.4%), and the answers contained useful information (432/494, 87.4%).

Participants generally found the visual content to be helpful (25/38, 65% of the participants indicated that the record linkage demonstration video was helpful; 31/38, 81% of the participants indicated that the visual image showing how the MiNDFIRL software reduces the privacy risk was helpful). Negative feedback about the demonstration video and image, however, was mainly related to technical issues (eg, could not view) and included a request for more information. The question that received the strongest negative feedback (18 participants either disagreed or strongly disagreed that the FAQ item contains useful information) was “What will you do if you discover that my data has been misused?” Many participants objected to the lack of specificity with the provided answer (“While we take great measures to safeguard your data, if a data breach were to occur, we would follow legal guidelines for breach notification.”) One participant noted:

What are the legal guidelines for breach notification? Are there any ramifications for the institution for a breach? Are there any remediation efforts that will be undertaken?

Of the 50 questions in round 1, 7 FAQ question and answer items were designated as *problematic* and triaged for substantial edits for round 2. In round 1, 5 participants (5/38, 13%) had concerns about the terminology used to describe information subsets (ie, *identifiers* and *nonidentifiers*).

### Delphi Round 2

A total of 37 (37/38, 97%) participants were included in round 2. Round 2 had fewer questions (25 items) than round 1 because of a high level of agreement and positive feedback. In general, the revisions made before round 2 were related to terminology and improving the readability of the FAQ. In addition, round 1 responses indicated that some concepts should be explained in more detail, and therefore, several new FAQ question and answer pairs were added to enhance the understanding (eg, “What is Patient Matching?”). We also included new visuals to aid understanding of key concepts.

In round 2, we sought specific feedback for the newly created FAQ items and the 7 FAQ items that were deemed problematic in round 1. Of these, strong majorities of participants agreed that the new and revised questions were easy to understand (mean 88%, SD 1.810), the answers were easy to understand (mean 88%, SD 1.732), the FAQ item was important (mean 83%, SD 1.5), and the FAQ item contained useful information (mean 90%, SD 1.536).

One of the FAQ items identified as problematic in round 1 received negative feedback concerning the terminology used to describe identifying information. Consequently, round 2 included two multiple choice questions and one open-ended question to identify the best terminology. In the round 1 FAQ, we used *identifiers* to refer to the subset of information within a record that is used in the record linkage process to distinguish one person from another, or conversely, confirm that 2 records pertain to the same individual. We also used the term *nonidentifiers* or *health-related study information* to refer to the information that researchers use to answer the research question. This terminology was critical to participants’ understanding of central concepts in the FAQ, namely, (1) researchers sometimes need identifying information such as names (eg, in record linkage), (2) identifying information is usually not needed when answering central research questions, and (3) researchers sometimes take steps to separate identifying information from the main research data before analysis (eg, coding data and using the MiNDFIRL software). There were several existing terms that were rejected as problematic either because they were terms of art with existing definitions that were not suited to a template FAQ for use in different research projects (eg, *protected health information*) or they were commonly used to refer to an entire record rather than subsets of the record (eg, *personally identifying information*).

In round 2, we asked participants for their preferred terms and included 4 options for each term, including some options suggested by participants in round 1. A plurality of round 2 participants voted for *identifying information* (16/38, 43%) and a majority of round 2 participants separately voted for *nonidentifying information* (23/37, 62%). An open-ended follow-up question allowed participants to offer additional thoughts on the suggested terms. A total of 4 participants offered support for *identifying information* or *nonidentifying information* as an alternative for their votes (Table 2).

Round 2 also contained a series of questions to improve the FAQ item pertaining to data misuse, which received the strongest negative feedback of the round 1 FAQ items. Creating a template FAQ language was particularly challenging given that the applicable laws, institutional policies, and ethical requirements are likely to vary significantly depending on the specific project [34]. Nonetheless, strong participant dissatisfaction with a vague reference to legal and institutional requirements clearly indicated that participants wanted more information. In round 2, we included 5 questions seeking feedback on what type of information participants would want for this FAQ item. Most participants agreed or strongly agreed that it was important to include links to legal requirements (30/37, 81%) or organizational rules (24/37, 65%) and thought it was important to summarize legal breach notification requirements (28/37, 76%), organizational notification requirements (25/37, 68%), or IRB required notification requirements (29/37, 78%).

Round 2 contained additional questions evaluating the revisions to the 6 other FAQ items deemed problematic in round 1 and the 3 newly added FAQ items. Participant feedback in round 2 indicated that only 3 of the FAQ items deemed problematic in round 1 were still problematic after the round 2 revisions (ie, at least three participants disagreed or strongly disagreed that the FAQ question or answer was easy to understand, important, or contained useful information). One of the open-ended comments suggested that 1 of the FAQ items remained problematic because it lacked a clear definition for *linked data*. Moreover, 1 FAQ item had conflicting feedback, specifically the need for detail versus brevity. The open-ended feedback suggested that the third FAQ item, “What difference will my data make?” was problematic for a variety of reasons, including FAQ clarity and apparent misalignment between the questions and answers included in the FAQ. In addition, several participants objected to the use of the phrase *people like you* when describing the representativeness of a sample.

**Table 2:** Terminology preferences of the participants for identifying and nonidentifying information (n=38).

Terminology preferences		Values, n (%)
**Subset of information used to link records (ie, identifying information)^a^**		
	Identifiers	3 (8)
	Identifying information	16 (43)
	Identifiable information	4 (11)
	Information that can be used to identify individuals	14 (38)
**Subset of information that is used to answer the research question (ie, nonidentifying information)^b^**		
	Nonidentifiers	2 (5)
	Nonidentifying information	23 (62)
	Nonidentifiable information	5 (14)
	Health-related study data	7 (19)

^a^Other terms suggested by participants: private information, personal information, patient data, confidential data, confidential information, information for identifying individuals, and personally identifiable (identifying) information.

^b^Other terms suggested by participants: data for health studies, publicly available data, social data, information that does not reveal your identity, anonymous study data, information that does not identify individuals, and information that is used as data.

All 3 of the newly introduced FAQ items were deemed problematic based on round 2 feedback. Of these new FAQ items, 1 described record linkage in general (“What is patient matching?”) and the other 2 specifically described the privacy-preserving record linkage software, MiNDFIRL. The negative open-ended feedback from these 3 questions indicated that some participants struggled with the length, complexity, and sometimes the tone (ie, patronizing) of the FAQ explanations. However, negative feedback on the length and complexity often directly conflicted with positive feedback related to detail and clarity. For example, 1 participant stated, *“*The technical terminology cannot be avoided. However, it is most informative. I would leave this section as is*.”* Similarly, although many participants indicated that the included visuals were helpful, the feedback indicated that the visuals were a point of confusion and frustration to others.

One new FAQ item (“How can MiNDFIRL help patient-matching while hiding identifiers?”) used a text narrative to describe how a researcher would use MiNDFIRL to conduct record linkage. Participants found this static text narrative clear, but tedious to follow. In round 3, the substance of this FAQ item was incorporated into a YouTube video demonstration (ie, FAQ text as the script) of the MiNDFIRL software [35].

### Delphi Round 3

A total of 33 (33/37, 89%) participants were included in round 3. We included 1 round 3 question that specifically solicited feedback on the preference of the participants for simplicity and brevity versus detail and completeness, (ie, whether we should eliminate 2 explanatory subsections of the FAQ item, “What pieces of information about me will the researchers see?”). A strong majority (25/33, 76%) of the participants preferred that the subsections be included for those who wanted the information.

On the basis of the feedback from rounds 1 and 2, we included a question in round 3 to solicit feedback on how to communicate statistics to the patient audience. The statistic described the proportion in percentage of identifying information that was revealed to a user of a MiNDFIRL software prototype as opposed to a user linking records without the software. Feedback from prior rounds suggested that patients would struggle to understand percentages. However, a majority of participants seemed to favor using percentages when asked in round 3.

In round 3, we provided alternative options for 2 problematic FAQ items. A majority of the participants (19/33, 58% and 20/33, 61%) favored the shorter alternative of each FAQ.

One FAQ item that was deemed problematic in round 2 was converted into a YouTube video demonstration for round 3 (Multimedia Appendix 8) [35]. Participants overwhelmingly found this video helpful or very helpful (31/33, 94%). Negative feedback was minor and focused on production, sound quality, and technical issues with playing the video.

In total, 3 of the 6 remaining problematic FAQ items had conflicting feedback in round 2. These items received negative feedback from a minority of our participants (10%-16%). For these questions, conflicting participant feedback in round 2 (ie, negative feedback vs positive feedback) raised doubts about substantial revisions to appease the minority of participants with negative feedback being able to maximize participant scores further. For example, we received positive feedback on the level of detail and completeness of these FAQ items and negative feedback requesting that the same FAQ items be shorter. We were unsure how to address these divergent comments with text edits. Thus, instead of adding specific questions in round 3, we revised these FAQ items where participant feedback indicated clear areas of improvement, for example, correcting visuals and adding requested definitions.

In addition, we attempted to address conflicting participant feedback on the competing values of simplicity and brevity versus detail and completeness by changing the format of the FAQ document in a few ways. First, we created an interactive website FAQ with expandable FAQ sections. This permitted users to quickly access sections that interest them and not be overwhelmed by the volume of other content. Second, we cut most definitions from the main text of the FAQ and replaced them with definition pop-up boxes that appear when a user’s mouse hovers over a key term. This reduced the volume of text while still providing information if needed. Third, we bolded the text of important information within each FAQ section to aid content skimming. A strong majority of the round 3 participants (31/33, 94%) found this revised format helpful or very helpful.

We asked our expert panel one 5-point Likert question on the overall helpfulness of the FAQ website. In total, 30 of the 33 respondents rated the FAQ website as *very helpful* (n=21), *helpful* (n=6), or *somewhat helpful* (n=3).

### Preliminary Thematic Analysis

The preliminary inductive thematic analysis identified 9 themes in the open-ended responses of the participants for all 3 rounds. These themes are summarized in Textbox 1. Representative quotes for these themes are available in Multimedia Appendix 9.

**Preliminary themes identified by inductive analysis and a brief description.**

Simplicity and brevity

- Expressing a preference for short and direct explanations

Detail and completeness

- Expressing a preference for complete explanations with sufficient details for clarity and understanding

Readability

- Expressing a preference for content that is easy to read and uses layman language

Terminology and definitions

- Expressing a preference for clearly defined terms and avoiding technical jargon

Tone

- Concerning the tone of explanations (eg, conversational and not patronizing)

Examples

- Concerning the utility of examples of key concepts

Visuals

- Concerning the utility of graphics, video, and interactive aids

Data disposition and future uses

- Expressing concerns relating to what happens to research data at the end of a project (eg, destruction, reuse, and storage)

Patient rights

- Concerning the explanation of patient rights and protection

### Participant Attrition

There were no significant participant attrition issues. Of the 38 participants who completed round 1, only 6 did not complete all 3 rounds. All 6 were women (aged 27-57 years) who reported having acquired a bachelor’s, master’s, or doctoral degree and reported fair to excellent health. Moreover, 4 of these participants were White.

## Discussion

### Principal Findings

Although the primary aim of this study is to develop a template language that could be used in a privacy statement for research using a specific software (MiNDFIRL), our results provide broad insights into communication with patients regarding how their data are used in research and how different software is used to enhance privacy. Communicating effectively with patients is an essential component of public health ethics, yet it is exceedingly difficult to communicate effectively [15,36]. Secondary database research uses an exceedingly broad range of research aims and methodologies. Moreover, the public is increasingly concerned about how their data are being used by the government, academia, and businesses [19,37]. Thus, proper transparency requires clearly describing the technical subject matter, communicating the purpose and process of data use, and addressing common concerns while using language that can be understood by a lay audience. To the best of our knowledge, this is the first effort to develop documentation in patient voice on an open-source software to facilitate transparent communication with the public on issues related to how a software addresses privacy concerns.

Our results indicate several considerations when communicating with patients. These considerations align with existing knowledge of health literacy (eg, simple language, visual aids, define terms, and cultural considerations) [38].

Although it may be obvious, our results support the notion that the patient community has varied preferences and opinions. This diversity manifested itself in comments about various aspects of the template disclosure language. For instance, there were frequent disagreements about the competing virtues of comprehensiveness versus conciseness, which might relate to varied levels of health literacy within our sample (ie, patients with chronic conditions) [38].

Communicating with the correct voice or tone when discussing highly technical subject matter can also be challenging. For instance, some of our participants expressed strong preferences for definitions of key terms. However, 1 respondent conveyed that an FAQ section with several definitions came across as a *textbook* answer. We used the second-person tense in our FAQ, which received positive feedback when used correctly (ie, personal and conversational). On the basis of participant feedback, we believe that a personal and conversational tone can be effective in cultivating trust between researchers and the public. However, we discovered that the second-person tense can come across as patronizing or worse. For example, when discussing representativeness, we used the phrase *people like you*. Although some participants had no issues with this phrase, several participants thought that the phrase had a negative tone and connotation and should be used carefully, if used at all.

Similarly, our results show that providing examples can prove to be beneficial and can create new challenges. Some participants found the examples to be helpful or requested examples for certain content. However, adding examples increases the content volume for the readers to absorb. If designed well, visuals can be particularly helpful to some patients. During the brief turnaround between Delphi rounds, we developed imperfect visuals that were helpful to many, but were confusing to others. Participant feedback was critical for identifying and correcting potentially confusing elements in the visuals.

The results from round 1 allow for another interesting observation. In round 1, we provided participants with a noninteractive FAQ document (PDF) and solicited feedback from participants as they progressed through each section of the document. The feedback from 8 of the 38 participants on earlier questions suggested that readers were developing questions and identifying concerns as they read the document. Although many of these questions and concerns were not germane to the section that they reviewed (and usually covered in detail subsequently), the feedback suggests that readers will either have preexisting questions that they want to be answered or will develop new questions as they read a privacy statement. This provides some support for an FAQ-style privacy statement, which identifies common questions and provides answers, as opposed to traditional privacy statements that can be challenging to navigate for personally relevant content [39].

The web-based, interactive FAQ received strong support. The interactive features (eg, expandable text and on-demand definitions) allowed us to provide information to those who want it, while reducing on-screen text for those who feel overwhelmed. This finding is consistent with other research showing preferences for web-based approaches in some populations [40].

We note that some organizations are moving away from web-based FAQs to communicate issues with the public [41]. For example, related FAQ answers might contain duplicative content, contributing to longer documents, and using questions as headings (as opposed to their answers) might slow a reader’s search for information. Our template FAQ included measures to control some of these concerns. For example, on-demand content for key terms and concepts reduces visible duplicative content, and highlighted key points aid readers in finding important information. However, future research should explore whether alternative modes of research transparency in secondary database research are preferable to an FAQ.

In addition to the FAQ-specific feedback, our participants identified a number of concerns that may be common in the broader patient community. For example, participants were interested in the use of encryption, the use of which seemed to relieve some concerns. Similar to prior work [33], patients also expressed concerns regarding the use of specific identifiers (ie, names and social security number). Several participants expressed questions and concerns relating to any future use, maintenance, or destruction of data pertaining to them. Researchers developing a privacy statement or disclosure for future research should consider including information on these topics to address these common concerns.

### Limitations

This study has several limitations. First, we set out to develop a template privacy statement language to be adapted for secondary database research projects using a specific record linkage software, MiNDFIRL. As a result, our template language may have limited applicability outside of secondary database research involving record linkage. Second, our *expert* patient Delphi panel may not broadly represent the diversity of patient experiences. We note that although our Delphi panel was mostly White, female, and well educated, patients who did not complete the study had similar characteristics. Although we observed saturation in the responses indicating that the coverage of issues was good among the participant group, future work should closely consider demographic differences, particularly for those populations that have been historically marginalized in research. For example, we conducted a subsequent survey of the FAQ with a nationally representative sample (ie, meeting US Census demographic targets) of more than 500 people. Initial results indicate that, in general, more than 80% were moderately, very, or extremely satisfied, but future work should focus on investigating the differential preferences by demographics and socioeconomic status, including education level, to better detect gaps. Third, the use of the Delphi method may have resulted in repeated exposure bias over the 3 rounds. Finally, the Delphi method is useful for identifying consensus, but it cannot measure effectiveness. Future evaluations should include pre-post testing to assess the effectiveness of this template language.

### Comparisons With Prior Work

There has been much work on understanding the experiences and perspectives of participants, especially in the context of using health data for research [5,9,10,17,26,40]. However, this study is the first to employ a Delphi method to engage patients with the objective of creating a privacy statement in an FAQ format to communicate issues, risks, and benefits of using their data and a software in a record linkage study.

### Conclusions

Although this study aimed to develop a communication tool for use with a specific record linkage prototype software (ie, MiNDFIRL), the lessons of this research have broad applicability to efforts of researchers to communicate with data subjects.

Our results support the existence of a diverse patient population with varied preferences and information needs. Despite these diverse preferences and needs, we were able to develop a consensus language that can help communicate complex research issues, including identifiability, record linkage, and technical privacy protections. We believe that this patient-friendly language is adaptable to other research contexts.

Moreover, our findings support a compromise between individuals who want detail and individuals who want brevity. An interactive FAQ document helps connect patients with the answers that they care about and enables on-demand additional content (ie, definitions, additional explanation, and examples) without cluttering the page for all readers. Adopting patient-friendly public disclosures relating to privacy safeguards and risks, such as the template FAQ, will help to promote transparency and trust in researchers among the public and the patient community.

## Appendix

Multimedia Appendix 1 Delphi round 1 survey instrument.

Multimedia Appendix 2 Delphi round 1 summary provided to round 2 participants.

Multimedia Appendix 3 Delphi round 2 survey instrument.

Multimedia Appendix 4 Delphi round 2 summary provided to round 3 participants.

Multimedia Appendix 5 Delphi round 3 survey instrument.

Multimedia Appendix 6 Delphi round 3 summary provided to Delphi participants.

Multimedia Appendix 7 Final web-based frequently asked question template language.

Multimedia Appendix 8 Video demonstration of Minimum Necessary Disclosure For Interactive Record Linkage provided to Delphi participants.

Multimedia Appendix 9 Thematic analysis with representative patient quotes.

## Abbreviations

FAQ: frequently asked question
IRB: institutional review board
MiNDFIRL: Minimum Necessary Disclosure For Interactive Record Linkage
NGT: nominal group technique
PCORI: Patient-Centered Outcomes Research Institute
PPRN: patient-powered research network
