# Supplementary material for: Communicating With Patients About Software for Enhancing Privacy in Secondary Database Research Involving Record Linkage: Delphi Study
Source: J Med Internet Res. 2020 Dec 15;22(12):e20783. doi: 10.2196/20783 (PMC7772068; doi:10.2196/20783)
Supplement: Multimedia Appendix 1 [file jmir_v22i12e20783_app1.pdf]

This is a Multimedia Appendix to a full manuscript published in the J Med Internet Res.
For full copyright and citation information see http://dx.doi.org/10.2196/jmir.20783

Setting

What is Patient Matching?

Patient Matching is the process of linking records of the same real-world person. Patient matching helps researchers answer difficult questions. For example, is disease treatment A better than treatment B for keeping patients healthy? To do this, we might want to count the number of emergency room visits Jane Doe made this year across several hospitals. This requires linking records from all the hospitals she visited.

This is hard because a universal identification number does not exist to easily link records in different systems. Instead, we have to use the personally identifiable information (PII), like social security numbers (SSNs), first & last names, birthdates, race, and gender to decide which records belong to the same person.

Still, patient matching is hard. Identifiers are not unique. Different people may share the same name. Names are inconsistent (e.g. nicknames). Data is sometimes missing (e.g. SSNs are often missing). Names change over time (e.g. changing a last name at marriage). Data can have errors (e.g. typos).

Let’s compare two databases. Which records do you think refer to the same person?

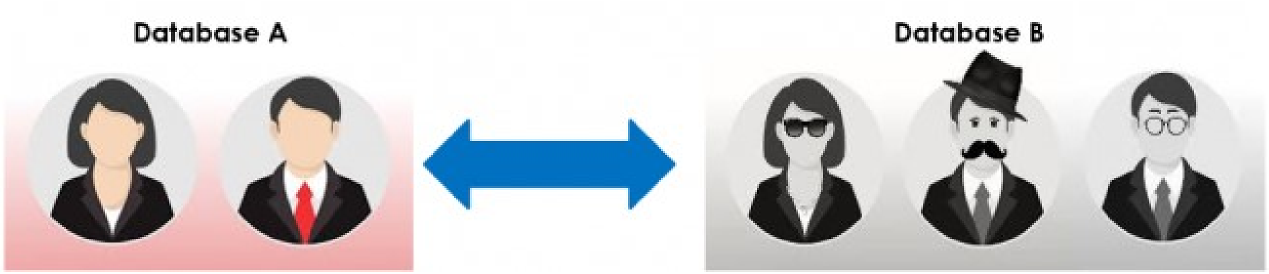

Privacy and Patient Matching in Retrospective Database Studies

Many studies of existing data use PII to match records from different databases. Getting consent for this type of research is often impossible because the researchers do not have contact with the individuals in the data. Thus, researchers try to reduce the privacy risks to ensure the research is ethical.

Ways to reduce risk include:

● Limit the people who can access the PII
● Restrict information uses
● Use secure computing systems
● Code data so the information can be studied without knowing who it belongs to

Goals & Procedures for this Survey

Researchers are trying to find new ways to protect privacy in patient matching. This survey is about a new patient-matching software. The software protects privacy in database studies. The software is called MINDFIRL. It stands for Minimum Necessary Disclosure For Interactive Record Linkage. We are developing a Frequently Asked Question (FAQ) page to help people understand how this software works to protect privacy in research. Ideally, these FAQs will be useful to help answer a patient’s questions about the use of his or her data in a database research study. We are asking for your help so that the FAQs include information important to you or others like you, and is easy to understand. This

study has three rounds. In the first round, we will ask for your opinion on a draft FAQ document. The FAQ describes a study that uses the MINDFIRL software for patient matching. In rounds 2 and 3, we will ask for your thoughts on the summary of anonymous responses from other survey participants so that we can get closer to consensus. The goal is to make the FAQ more useful to patients like you.

Please answer the following questions:

There are two sections to this survey.

- Section 1. Background - We will ask basic demographics questions (round 1 only)
- Section 2: Questions about the understandability, usefulness, and importance of questions and answers to sections of the FAQ. Note that some questions will ask you to focus on the questions covered in the FAQ, and other questions will ask about both the question and answers in the FAQ.

****Please remember to use Google Chrome to complete the survey.**

Background Information

1. What is your age as of your last birthday?

2. What is the highest degree or level of school you have completed?

Some high school, no diploma

High school graduate or the equivalent (e.g. GED)

Some college credit, no degree

Associate degree

Bachelor's degree

Master's degree

Professional degree (e.g. MD, JD, DDS, DVM)

Doctoral degree (e.g. PhD, EdD, DrPH)

3. Which gender do you identify with?

Male

Female

Non-binary/third gender

Prefer to self-describe:

I would prefer not to comment

4. What is your race?

American Indian or Alaska Native

Black or African American

Asian

Pacific Islander

White

Other race

5. Are you Hispanic?

Yes

No

6. On average, how many times a year do you seek care from a healthcare professional for yourself?

1

2 – 5

6 - 10

>10

7. In general, how would you describe your health?

Excellent

Very Good

Good

Fair

Poor

8. [Optional] If you feel comfortable, please let us know whether you live with a chronic condition and what the condition is:

Delhpi Survey

DELPHI SURVEY

Scenario: Assume that you are a patient who gets health care several times each year. You see different doctors in different hospitals. One hospital emails you and says that your data will be used in a research study. The researchers will use personally identifiable information (PII) to match the records of the same people in different hospitals. The researchers have decided to use the MINDFIRL software to match these records. The hospital's email includes a link to a FAQ about the research.

The following are questions about the FAQ that we would like your help in writing. Click
here to get the full pdf of the FAQ. We recommend that you either print the pdf of the FAQ or keep it opened on your screen for your reference. It may be useful to read the whole document before you start the survey.

Section 2. In the following section, please let us know your thoughts about the questions and answers in the FAQ.

Frequently Asked Questions

Section 2.1: Questions about the data and identifiers

FAQ 1. Why do you need to know who I am?
Our goal is to match records from different databases. Thus, we need to know some limited information about you so we do not mismatch your records with someone else's. We refer to this limited information as 'identifiers.'

2.1.a) Please tell us what you think about the following statements.

	Strongly agree	Agree	Neither agree nor disagree	Disagree	Strongly disagree
The question is easy to understand.	○	○	○	○	○
The question contains useful information.	○	○	○	○	○
The answer is easy to understand.	○	○	○	○	○
The answer contains useful information.	○	○	○	○	○

FAQ 2. What are identifiers?

Identifiers are pieces of information that help us distinguish you from someone else. These may include your date of birth, your gender, your marital status, your race or ethnicity, your identification number, or your name, among other things.

2.1.b) Please tell us what you think about the following statements.

	Strongly agree	Agree	Neither agree nor disagree	Disagree	Strongly disagree
The question is easy to understand	○	○	○	○	○
The question contains useful information.	○	○	○	○	○
The answer is easy to understand	○	○	○	○	○
The answer contains useful information.	○	○	○	○	○

FAQ 3. What information about me will the researchers see?

The researchers need different information for different steps of the research process. We only need identifiers to do patient matching. Additionally, we only need non-identifiers when we are using your data to learn more about science or medicine.

a. Identifiers

The researchers that will be doing the record matching will have access to identifiers. Information such as your name, date of birth, marital status, and gender help distinguish you from other people. Researchers need to access identifiers to match patient records.

We are using the MINDFIRL software to protect identifiers and prevent unnecessary privacy loss during this process. For example, MINDFIRL tells researchers when two records have the same identifiers without showing details.

In these cases, researchers might not need to see specific identifiers to make a match. MINDFIRL also tells researcher when records are highly similar without showing details. MINDFIRL only shows identifiers on an ‘as needed’ basis. For example, a researcher might want to see some details to know if a difference is important. This means that MINDFIRL can help catch common matching problems, such as nicknames (e.g., Pam v. Pamela) or typos, without showing the rest of your identifiers.

b. Non-identifiers, health-related study data

Non-identifiers are everything else in the data. Non-identifiers could include information such as diagnosis, medications, or blood pressure. We will only use the non-identifiers for the main research after the matching is done. After matching records, MINDFIRL separates identifiers from the non-identifiers. Thus, no one can access the identifiers AND the health-related data at the same time. We will code your non-identifiers to protect your identity. This allows us to use your information to make scientific or medical discoveries without knowing which information belongs to you.

2.1.c) Please tell us what you think about the following statements.

	Strongly agree	Agree	Neither agree nor disagree	Disagree	Strongly disagree
The question is easy to understand.	○	○	○	○	○
The question contains useful information.	○	○	○	○	○
The answer is easy to understand.	○	○	○	○	○
The answer contains useful information.	○	○	○	○	○

FAQ 4. If a researcher sees my name in the data when matching, how much will they know about me?

If a researcher sees your name in the data, they might see some more identifiers about you (e.g., birth dates, identification numbers, race, gender). But they will not see other health details, such as your diagnosis, health status, blood pressure, etc. Your name is an identifier. We only use identifiers during the patient-matching step of the research to help link records from different databases. In this step other details about you, such as your health information, are not used to match records. We only use this health-related data once we remove your identifiers to protect your privacy.

2.1.d) Please tell us what you think about the following statements.

	Strongly agree	Agree	Neither agree nor disagree	Disagree	Strongly disagree
The question is easy to understand.	○	○	○	○	○
The question contains useful information.	○	○	○	○	○
The answer is easy to understand.	○	○	○	○	○
The answer contains useful information.	○	○	○	○	○

2.1.e) Questions 1 - 4 were about information and how it is used in research. Is there anything more that you would like to know on this topic?
(Type "No" if you do you not have any comment)

2.1.f) Do you have any concerns that you would like the FAQ to address about information and how it is used in research?
(Type "No" if you do you not have any comment)

Section 2.2: Questions about MINDFIRL and the patient matching process

FAQ 5. What does MINDFIRL look like?
Click below to see a video of MINDFIRL and to try matching records with it.

http://mindfil4.herokuapp.com/?mode=4

2.2.a) Was the video helpful?

Yes No

2.2.b) Please provide a rationale for your answer on whether the video was helpful or not.

2.2.c) Was the interactive demonstration helpful?

Yes No

2.2.d) Please provide a rationale for your answer on whether the interactive demonstration was helpful or not.

FAQ 6. What information do you need in order to match my records?

For some people, very few identifiers are needed to match records. For example, records from someone with a very unique name may be easily matched with just a name and perhaps their date of birth. In contrast, the records for someone with a common name may be harder for the researchers to accurately match. As a result, additional information such as gender, race or ethnicity, and identification numbers may be needed.

2.2.e) Please tell us what you think about the following statements.

	Strongly agree	Agree	Neither agree nor disagree	Disagree	Strongly disagree
The question is easy to understand.	○	○	○	○	○
The question contains useful information.	○	○	○	○	○
The answer is easy to understand.	○	○	○	○	○
The answer contains useful information.	○	○	○	○	○

FAQ 7. Can I be identified in the linked data?

In the age of big data, it is almost impossible to make a dataset fully anonymized

and useful at the same time. However, the data we analyze for our research will not contain the identifiers used to match records. Instead, we will remove all identifiers from the matched data and [Researcher should select (1) keep them in a separate file, or (2) destroy them] before it is used for analysis.

2.2.f) Please tell us what you think about the following statements.

	Strongly agree	Agree	Neither agree nor disagree	Disagree	Strongly disagree
The question is easy to understand.	O	O	O	O	O
The question contains useful information.	O	O	O	O	O
The answer is easy to understand.	O	O	O	O	O
The answer contains useful information.	O	O	O	O	O

FAQ 8. Are there risks to patient matching?

Patient matching is not very risky on its own. There is always a slight chance that someone outside our research group may try to gain access to your data or that someone matching the data may misuse it. We work hard to minimize these risks. First, we store all data on secure computers that meet legal standards to minimize the risk of someone breaking into the data. Second, each researcher on this project is trained to use the software properly and comply with the law. These efforts will minimize misuse of the data.

2.2.g) Please tell us what you think about the following statements.

	Strongly agree	Agree	Neither agree nor disagree	Disagree	Strongly disagree
The question is easy to understand.	O	O	O	O	O
	O	O	O	O	O

	Strongly agree	Agree	Neither agree nor disagree	Disagree	Strongly disagree
The question contains useful information.					
The answer is easy to understand.	○	○	○	○	○
The answer contains useful information.	○	○	○	○	○

FAQ 9. Does MINDFIRL reduce risks to patient matching?

Yes, MINDFIRL is designed to improve privacy in database studies. Thus, we expect that MINDFIRL will lower the risk of individual studies. MINDFIRL includes tools to promote transparency and researcher accountability to limit risk. For example, MINDFIRL tracks what identifiers are viewed and who viewed them. This information is used to discourage the misuse of your information. It also allows for setting hard limits on how much data is used. See figure below.

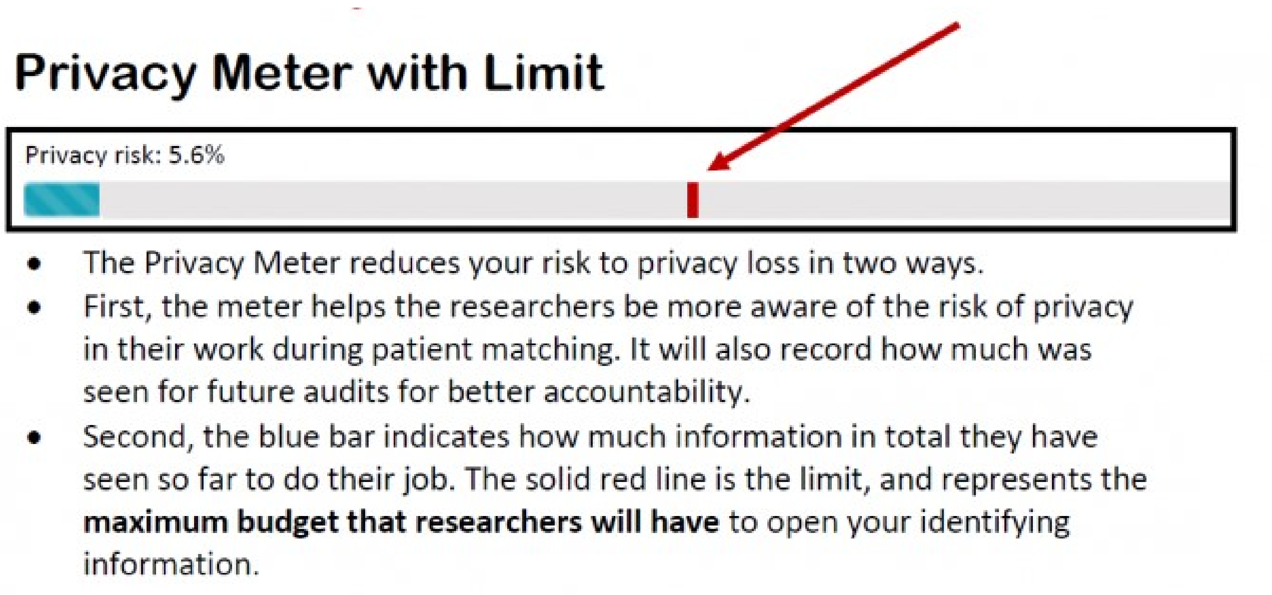

2.2.h) Please tell us what you think about the following statements.

	Strongly agree	Agree	Neither agree nor disagree	Disagree	Strongly disagree
The question is easy to understand.	○	○	○	○	○
The question contains useful information.	○	○	○	○	○
The answer is easy to understand.	○	○	○	○	○
The answer contains useful information.	○	○	○	○	○
The picture and explanation above were helpful to understand how MINDFIRL works.	○	○	○	○	○

FAQ10. How does MINDFIRL enhance privacy?

MINDFIRL improves privacy by greatly reducing the identifying information viewed by researchers. MINDFIRL only shows identifiers if a researcher thinks they need it to match patient records accurately. In addition, MINDFIRL gives researchers clues to help researchers understand if two masked identifiers are the same, similar, or different. This helps researchers match records without revealing identifying information.

2.2.i) Please tell us what you think about the following statements.

	Strongly agree	Agree	Neither agree nor disagree	Disagree	Strongly disagree
The question is easy to understand.	○	○	○	○	○
The question contains useful information.	○	○	○	○	○
The answer is easy to understand	○	○	○	○	○

	Strongly agree	Agree	Neither agree nor disagree	Disagree	Strongly disagree
The answer contains useful information.	○	○	○	○	○

FAQ 11. Does MINDFIRL reduce the accuracy of matched records?
No. One study showed that people who used an early version of MINDFIRL were just as accurate as people who saw 100% of the identifiers. However, the people who used MINDFIRL only saw 7% of the identifiers.

2.2.j) Please tell us what you think about the following statements.

	Strongly agree	Agree	Neither agree nor disagree	Disagree	Strongly disagree
The question is easy to understand.	○	○	○	○	○
The question contains useful information.	○	○	○	○	○
The answer is easy to understand.	○	○	○	○	○
The answer contains useful information.	○	○	○	○	○

2.2.k) Questions 5 - 11 were about MINDFIRL and the patient-matching process. Is there anything more that you would like to know on this topic?
(Type "No" if you do you not have any comment)

2.2.I) Do you have any concerns that you would like the FAQ to address about MINDFIRL and the patient-matching process?
(Type "No" if you do you not have any comment)

The following sections contain FAQ questions that might have different answers depending on the specific research project. Researchers who use this FAQ for their projects will have to fill out the answers with information specific to their project. For the following sections we want you to focus on the questions and any RED portions of the answers.

Section 2.3: Questions about where and how my matched data will be stored and protected

FAQ 12. Where will data about me be kept?

a. Identifiers

Your identifying information will be kept… [This information will vary depending on the specific research project and protocol. Researchers will describe how they will store and protect information used for the study]

One example response might be: All research data is stored in secure server at Texas A&M University. The Texas A&M University Information Technology (IT) department maintains the servers. See the next section on how the data is secured on this server. Your identifiers will be kept in a separate secure location with different access controls (e.g., password protection). Only the staff involved with patient matching will have access to this folder.

b. Non-identifiers

Your non-identifying information such as diagnosis type, medications, etc., will be kept…[This information will vary depending on the specific research project and protocol. Researchers will describe how they will store and protect information used for the study]

One example response might be: The non-identifiers is stored in the same secured server as the identifiers. However, the non-identifiers will be stored in a different secure folder from the identifiers. Only staff conducting analysis with the data will have access to the folder with the non-identifying data.

2.3.a) Please tell us what you think about the following statements.

	Strongly agree	Agree	Neither agree nor disagree	Disagree	Strongly disagree
The question is easy to understand.	○	○	○	○	○
I believe that an answer to this question would contain useful information.	○	○	○	○	○

2.3.b) How important is it for researchers to answer the question above (FAQ 12)?

Very important	Important	Moderately important	Slightly important	Not important

FAQ 13. What security measures are you using to protect my data?

[This information will vary depending on the specific research project and protocol. Researchers will describe the specific security measures for the study]

Some possible answers might be:
• The server is located in the secure facility with 24/7 monitoring
• Dual authentication (For example, a password and a code sent to a researcher's phone)
• Firewall
• Virtual Private Network (VPN) encrypted connections
• No data is allowed to be taken off the server
• Identifiers are stored separately from non-identifiers
• Using MINDFIRL to reduce access to identifiers
• Security audits for compliance.

2.3.c) Please tell us what you think about the following statements.

	Strongly agree	Agree	Neither agree nor disagree	Disagree	Strongly disagree
The question is easy to understand.	○	○	○	○	○
I believe that an answer to this question would contain useful information.	○	○	○	○	○

2.3.d) How important is it for researchers to answer the question above (FAQ 13)?

Very important	Important	Moderately important	Slightly important	Not important

FAQ 14. What are you doing to make sure that my data is being used responsibly?

This research was reviewed by the institutional review board (IRB) at [Researchers will fill in their IRB information]. The IRB oversees research to make sure it is legally and ethically permissible. We are also using the

MINDFIRL software for record linkage to limit access to information that can identify you. This is part of our commitment to conducting responsible research. Furthermore, … [This information will vary depending on the specific research project, protocol, and institutional policies. MINDFIRL allows researchers to customize settings for transparency and accountability. Researchers will describe the specific safeguards that are in place to ensure responsible data use, including policies, MINDFIRL settings, and required trainings.]

2.3.e) Please tell us what you think about the following statements.

	Strongly agree	Agree	Neither agree nor disagree	Disagree	Strongly disagree
The question is easy to understand.	○	○	○	○	○
The question contains useful information.	○	○	○	○	○
The answer is easy to understand.	○	○	○	○	○
The answer contains useful information.	○	○	○	○	○

2.3.f) How important is it for researchers to answer the question above (FAQ 14)?

Very important	Important	Moderately important	Slightly important	Not important

FAQ 15. What will you do if you discover that my data has been misused?
While we take great measures to safeguard your data, if a data breach were to occur, we would follow legal guidelines for breach notification.

2.3.g) Please tell us what you think about the following statements.

	Strongly agree	Agree	Neither agree nor disagree	Disagree	Strongly disagree
The question is easy to understand.	○	○	○	○	○
The question contains useful information.	○	○	○	○	○
The answer is easy to understand.	○	○	○	○	○
The answer contains useful information.	○	○	○	○	○

2.3.h) How important is it for researchers to answer the question above (FAQ 15)?

Very important	Important	Moderately important	Slightly important	Not important

2.3.i) Questions 12 – 15 were about how information is stored and protected in research. The answers in this section will be different depending on the specific research project. Is there anything more that you would like to know on this topic?
(Type "No" if you do you not have any comment)

2.3.j) Do you have any concerns that you would like the FAQ to address about how information is stored and protected in research?
(Type "No" if you do you not have any comment)

Section 2.4: Questions about the researchers

FAQ 16. Who will have access to my data?
[This information will vary depending on the specific research project. Researchers will describe the qualifications for authorized data users and should consider identifying the research team.]

2.4.a) Please tell us what you think about the following statements.

	Strongly agree	Agree	Neither agree nor disagree	Disagree	Strongly disagree
The question is easy to understand.	○	○	○	○	○
I believe that an answer to this question would contain useful information.	○	○	○	○	○

2.4.b) How important is it for researchers to answer the question above (FAQ 16)?

Very important	Important	Moderately important	Slightly important	Not Important

2.4.c) Question 16 was about the research team. Is there anything more that you would like to know on this topic?
(Type "No" if you do you not have any comment)

2.4.d) Do you have any concerns that you would like the FAQ to address about the research team?
(Type "No" if you do you not have any comment)

Section 2.5: Questions about the impact my data will have

FAQ 17. Why is my data needed?

Your data is needed so that we can better understand … [This information will vary depending on the specific research project. Researchers will describe the specific research question and provide enough context for readers to understand the problem the research is trying to address.]

2.5.a) Please tell us what you think about the following statements.

	Strongly agree	Agree	Neither agree nor disagree	Disagree	Strongly disagree
The question is easy to understand.	○	○	○	○	○

	Strongly agree	Agree	Neither agree nor disagree	Disagree	Strongly disagree
I believe that an answer to this question would contain useful information.	○	○	○	○	○

2.5.b) How important is it for researchers to answer the question above (FAQ 17)?

Very important	Important	Moderately important	Slightly important	Not important

FAQ 18. How is my data contributing to or advancing science?

[This information will vary depending on the specific research project. Researchers will try to help readers understand how their research contributes to addressing a specific problem. For example, "Your data is advancing our knowledge of problem A in that it helps us determine associations between X and Y."]

2.5.c) Please tell us what you think about the following statements.

	Strongly agree	Agree	Neither agree nor disagree	Disagree	Strongly disagree
The question is easy to understand.	○	○	○	○	○
I believe that an answer to this question would contain useful information.	○	○	○	○	○

2.5.d) How important is it for researchers to answer the question above (FAQ 18)?

Very important	Important	Moderately important	Slightly important	Not important

FAQ 19. What difference is my data going to make?

In research, we use information about a group of people, called a “sample,” to understand things about a larger group or “population.” If the sample is too different from the larger population then we cannot learn very much from the research. If people like you are not included in the research, then what we learn will not be useful to you or others like you. For example, if young adults are excluded from all studies about drug safety, it will be difficult to ever know if any drugs are safe to use on young adults.

2.5.e) Please tell us what you think about the following statements.

	Strongly agree	Agree	Neither agree nor disagree	Disagree	Strongly disagree
The question is easy to understand.	○	○	○	○	○
The question contains useful information.	○	○	○	○	○
The answer is easy to understand.	○	○	○	○	○
The answer contains useful information.	○	○	○	○	○

2.5.f) Questions 17 – 19 were about the potential impact of your data. Is there anything more that you would like to know on this topic?
(Type "No" if you do you not have any comment)

2.5.g) Do you have any concerns that you would like the FAQ to address about the potential impact of your data?
(Type "No" if you do you not have any comment)

Section 2.6: Questions about what happens to my data once the study is completed

FAQ 20. What will happen to my data after this study is completed?
[This information will vary depending on the specific research project. Researchers will discuss their plans to destroy, store, or reuse the study data.]

2.6.a) Please tell us what you think about the following statements.

	Strongly agree	Agree	Neither agree nor disagree	Disagree	Strongly disagree
The question is easy to understand.	○	○	○	○	○
I believe that an answer to this question would contain useful information.	○	○	○	○	○

2.6.b) How important is it for researchers to answer the question above (FAQ 20)?

Very important	Important	Moderately important	Slightly important	Not important

FAQ 21. Will my matched data be used for other research studies?

[This information will vary depending on the specific research project. Researchers will discuss any plans to reuse the study data.]

2.6.c) Please tell us what you think about the following statements.

	Strongly agree	Agree	Neither agree nor disagree	Disagree	Strongly disagree
The question is easy to understand.	○	○	○	○	○
I believe that an answer to this question would contain useful information.	○	○	○	○	○

2.6.d) How important is it for researchers to answer the question above (FAQ 21)?

Very important	Important	Moderately important	Slightly important	Not important

FAQ 22. Where can I get more information?

[This information will vary depending on the specific research project. Researchers provide contact information.]

2.6.e) Please tell us what you think about the following statements.

	Strongly agree	Agree	Neither agree nor disagree	Disagree	Strongly disagree
The question is easy to understand.	○	○	○	○	○
I believe that an answer to this question would	○	○	○	○	○

	Strongly agree	Agree	Neither agree nor disagree	Disagree	Strongly disagree
contain useful information.					

2.6.f) How important is it for researchers to answer the question above (FAQ 22)?

Very important	Important	Moderately important	Slightly important	Not important

2.6.g) Questions 20 -- 22 were about what happens to my data once the study is completed. Is there anything more that you would like to know on this topic?
(Type "No" if you do you not have any comment)

2.6.h) Do you have any concerns that you would like the FAQ to address about what happens to my data once the study is completed?
(Type "No" if you do you not have any comment)

Section 2.7: Overall FAQ

2.7.a) Do you have any other comments about this FAQ overall?
(Type "No" if you do you not have any comment)

2.7.b) Please type 'Agree' if you are satisfied with the order of the sections as they were presented. If you were not satisfied, indicate below the order in which you think they should appear.

Section 2.1: Questions about the data and identifiers
Section 2.2: Questions about MINDFIRL and the patient matching process
Section 2.3: Questions about where and how my matched data will be stored and protected
Section 2.4: Questions about the researchers
Section 2.5: Questions about the impact my data will have
Section 2.6: Questions about what happens to my data once the study is completed

Giftcard

We thank you for your participation in our Delphi study.

Please select your preferred method of payment:

Amazon gift card

Target gift card

Powered by Qualtrics
