# Supplementary material for: Communicating With Patients About Software for Enhancing Privacy in Secondary Database Research Involving Record Linkage: Delphi Study
Source: J Med Internet Res. 2020 Dec 15;22(12):e20783. doi: 10.2196/20783 (PMC7772068; doi:10.2196/20783)
Supplement: Multimedia Appendix 2 [file jmir_v22i12e20783_app2.pdf]

This is a Multimedia Appendix to a full manuscript published in the J Med Internet Res.
For full copyright and citation information see http://dx.doi.org/10.2196/jmir.20783

SUMMARY OF ROUND 1

There were 38 respondents in Round 1. Based on your responses, there were five questions and accompanying template responses that were particularly problematic, and two additional questions and accompanying template language that were somewhat problematic. The table below provides a summary of the responses received on these seven items.

**Particularly Problematic**	
**Question and Template Response**	**Summary of Responses**
**1. What information about me will the researchers see? [2.1.3]**<br>**Template Response:**<br>The researchers need different information for different steps of the research process. We only need identifiers to do patient matching. Additionally, we only need non-identifiers when we are using your data to learn more about science or medicine.<br>a. Identifiers<br>The researchers that will be doing the record matching will have access to identifiers. Information such as your name, date of birth, marital status, and gender help distinguish you from other people. Researchers need to access identifiers to match patient records.<br><br>We are using the MINDFIRL software to protect identifiers and prevent unnecessary privacy loss during this process. For example, MINDFIRL tells researchers when two records have the same identifiers without showing details. In these cases, researchers might not need to see specific identifiers to make a match. MINDFIRL also tells researcher when records are highly similar without showing details. MINDFIRL only shows identifiers on an 'as needed' basis. For example, a researcher might want to see some details to know if a difference is important. This means that MINDFIRL can help catch common matching problems, such as nicknames (e.g., Pam v. Pamela) or typos, without showing the rest of your identifiers.	Seven (7) participants disagreed or strongly disagreed that the template answer provide contains useful information. One (1) participant strongly disagreed that the question is easy to understand, and 1 participant disagreed that the answer provides useful information. Seven participants neither agreed or disagreed that the question and answer contain useful information or that the answer was easy to understand.<br><br>Suggestions to improving the response to the question included telling patients that the information that researchers will see is information that healthcare providers already have collected on their patients during previous visits and a sense of where the data is pulled from. Additionally, one participant suggested that a statement on whether the persons doing the linkage and the persons doing the research are the same. In addition, it was suggested that a statement on whether the general release of information that patients sign when seeking care is what allows the use of patient data for the research study.<br><br>Several participants indicated that the reading/required literacy level for the response as it stands is too high, and that words such as 'identifying information' and 'non-identifying information' would be more appropriate instead of 'identifiers' and 'non-identifiers.'

b. Non-identifiers, health-related study data<br>Non-identifiers are everything else in the data. Non-identifiers could include information such as diagnosis, medications, or blood pressure. We will only use the non-identifiers for the main research after the matching is done. After matching records, MINDFIRL separates identifiers from the non-identifiers. Thus, no one can access the identifiers AND the health-related data at the same time. We will code your non-identifiers to protect your identity. This allows us to use your information to make scientific or medical discoveries without knowing which information belongs to you.	
**2. Can I be identified in the linked data? [2.2.7]**<br>**Template Response**<br>In the age of big data, it is almost impossible to make a dataset fully anonymized and useful at the same time. However, the data we analyze for our research will not contain the identifiers used to match records. Instead, we will remove all identifiers from the matched data and [*Researcher should select* (1) keep them in a separate file, or (2) destroy them] before it is used for analysis.	Four (4) participants strongly disagreed that the question is easy to understand. In terms of whether the question contains useful information, 6 participants neither agreed nor disagreed, and 1 particular strongly disagreed. Ten (10) participants indicated that they disagreed or strongly disagreed that the template response provided was easy to understand, while 6 participants neither agreed nor disagreed. Seven (7) respondents neither agreed nor disagreed that the template response contains useful information, and 3 participants disagreed or strongly disagreed that it did.<br><br>One participant suggested that a definition of 'linked data' be provided for clarity. Another participant indicated that the response is confusing because it states that all identifiers will be removed, but implies that a researcher could click on identifying cells to get additional information for a match decision.
**3. Does MINDFIRL reduce the quality of matched records? [2.2.11]**<br>**Template Response**<br>No. One study showed that people who used an early version of MINDFIRL were just as accurate as people who saw 100% of the identifiers. However, the people who used MINDFIRL only saw 7% of the identifiers.	Four (4) participants indicated that they disagreed or strongly disagreed that the question is easy to understand, while 2 participants indicated that they neither agreed nor disagreed with this statement. In terms of whether the question contains useful information, 3 participants disagreed or strongly disagreed and 4 participants neither agreed nor disagreed. Four (4) participants disagreed or strongly disagreed that the provided template response was easy to understand, and 5 participants disagreed or strongly disagreed that the answer contains useful information. Notably, one participant provided alternative wording to 2.2.11 to instead ask, "Does using MINDFIRL to use the minimum required identifiers impact the accuracy of patient matching?" Additionally, several participants

	indicated that the wording of the response was at too high a reading/literacy level and suggested that less technical language be used to answer the question.
**4. What will you do if you discover that an unauthorized person has accessed my data or my data has been otherwise misused [2.3.15]**<br>**Template Response**<br>While we take great measures to safeguard your data, if a data breach were to occur, we would follow legal guidelines for breach notification.	Eighteen (18) participants disagreed or strongly disagreed that the provided template response contains useful information, while 6 indicated that they disagreed or strongly disagreed that the response was easy to understand. Twenty-nine (29) participants indicated that it is very important for researchers to provide an answer to the question in their FAQ, while 7 indicated that it was important.<br><br>A few participants wondered what exactly constituted 'legal guidelines' and 'breach notification,' and suggested that more details be provided on what they entail. One participant suggested providing links to additional resources, while another suggested that more details should be provided on the process that would be followed should a breach occur. Information on who would be notified, how soon notification would occur, and whether the project would continue with or without the breached data were suggested additions.<br><br>Other suggestions included the inclusion of a name, email, and phone contact information for patients wishing to contact someone if they believe that their PII has been misused.
**5. What difference is my data going to make? [2.5.18]**<br>**Template Response**<br>In research, we use information about a group of people, called a "sample," to understand things about a larger group or "population." If the sample is too different from the larger population then we cannot learn very much from the research. If people like you are not included in the research, then what we learn will not be useful to you or others like you. For example, if young adults are excluded from all studies about drug safety, it will be difficult to ever know if any drugs are safe to use on young adults.	Four (4) participants disagreed or strongly disagreed that the provided template response was easy to understand, while 3 participants indicated that they disagreed or strongly disagreed that the answer contains useful information. One participant stated that the answer to the question was not provided in the template response.
**Somewhat Problematic**	
**Question and Template Response**	**Summary of Responses**
**6. Does MINDFIRL reduce risks to patient matching? [2.2.9]**<br>**Template Response**	Three (3) participants neither agreed nor disagreed that the question contains useful information, and 3 neither agreed nor disagreed that the provided

<table>
<tr>
<td>Yes, MINDFIRL is designed to improve privacy in database studies. Thus, we expect that MINDFIRL will lower the risk of individual studies. MINDFIRL includes tools to promote transparency and researcher accountability to limit risk. For example, MINDFIRL tracks what identifiers are viewed and who viewed them. This information is used to discourage the misuse of your information. It also allows for setting hard limits on how much data is used. See figure below.

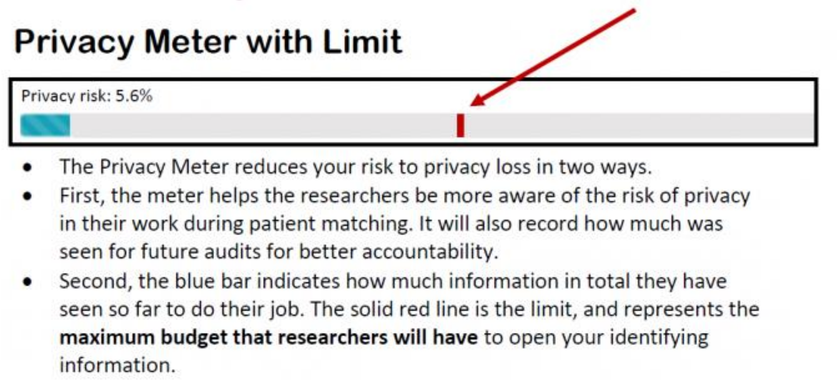
</td>
<td>template response contains useful information. Two (2) participants disagreed or strongly disagreed that the provided template response was easy to understand.

A few respondents indicated that the use of the word ‘budget’ in the figure was not ideal. Suggestions for a different word included ‘firm limit’ or ‘cap.’</td>
</tr>
<tr>
<td>7. What are you doing to make sure that my data are being used responsibly? [2.3.14]
Template Response
This research was reviewed by the institutional review board (IRB) at [Researchers will fill in their IRB information]. The IRB oversees research to make sure it is legally and ethically permissible. We are also using the MINDFIRL software for record linkage to limit access to information that can identify you. This is part of our commitment to conducting responsible research. Furthermore, … [This information will vary depending on the specific research project, protocol, and institutional policies. MINDFIRL allows researchers to customize settings for transparency and accountability. Researchers will describe the specific safeguards that are in place to ensure responsible data use, including policies, MINDFIRL settings, and required trainings.</td>
<td>Two (2) participants indicated that they disagreed or strongly disagreed that the provided template response is easy to understand, while 7 indicated that they neither agreed nor disagreed. Three (3) participants neither agreed nor disagreed that the question contains useful information.

Participant suggestions included wording on what ‘data being used responsibly,’ and ‘legal guidelines for breach notification’ mean, as patients may not be familiar with these concepts. Additionally, one participant suggested that the FAQ include information on what institutional review and an IRB are. Specifically, suggestion questions for the FAQ included, “What is the IRB?’ and ‘’Why is the IRB important?’ Several participants cautioned that terms need to be explicitly defined for patients as it cannot be assumed that they are familiar with terms that researchers may be familiar with.</td>
</tr>
</table>

In addition to specifically noting the specific questions and template responses that need to be significantly edited, you may be interested in a breakdown of the responses to each question and template response in the FAQ. The graphs below provide this breakdown.

Frequently Asked Questions
<u>Section 2.1: Questions about the data and identifiers</u>

1. Why do you need to know who I am?
   Our goal is to match records from different databases. Thus, we need to know some limited information about you so we do not mismatch your records with someone else's. We refer to this limited information as 'identifiers.'

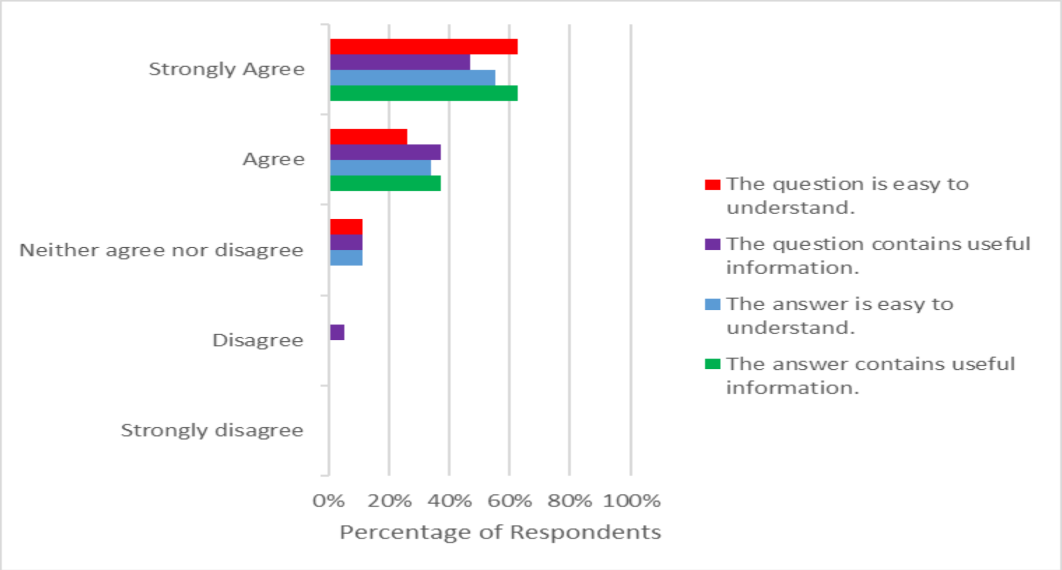

2. What are identifiers?
   Identifiers are pieces of information that help us distinguish you from someone else. These may include your date of birth, your gender, your marital status, your race or ethnicity, your identification number, or your name, among other things.

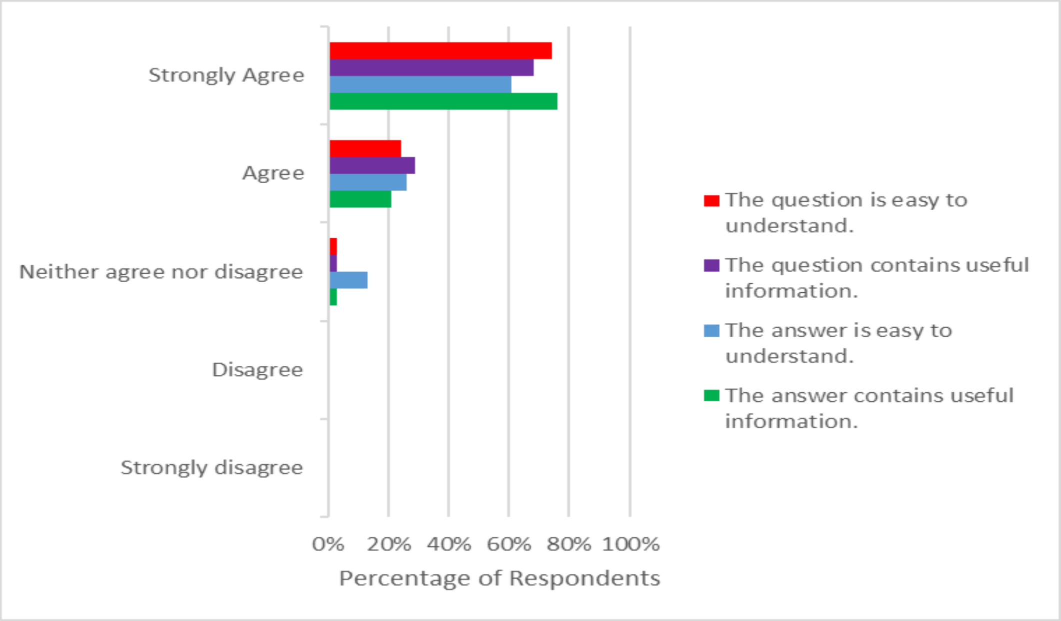

3. What information about me will the researchers see?
The researchers need different information for different steps of the research process. We only need identifiers to do patient matching. Additionally, we only need non-identifiers when we are using your data to learn more about science or medicine.
    a. Identifiers
    The researchers that will be doing the record matching will have access to identifiers. Information such as your name, date of birth, marital status, and gender help distinguish you from other people. Researchers need to access identifiers to match patient records.

    We are using the MINDFIRL software to protect identifiers and prevent unnecessary privacy loss during this process. For example, MINDFIRL tells researchers when two records have the same identifiers without showing details. In these cases, researchers might not need to see specific identifiers to make a match. MINDFIRL also tells researcher when records are highly similar without showing details. MINDFIRL only shows identifiers on an 'as needed' basis. For example, a researcher might want to see some details to know if a difference is important. This means that MINDFIRL can help catch common matching problems, such as nicknames (e.g., Pam v. Pamela) or typos, without showing the rest of your identifiers.
    b. Non-identifiers, health-related study data
    Non-identifiers are everything else in the data. Non-identifiers could include information such as diagnosis, medications, or blood pressure. We will only use the non-identifiers for the main research after the matching is done. After matching records, MINDFIRL separates identifiers from the non-identifiers. Thus, no one can access the identifiers AND the health-related data at the same time.

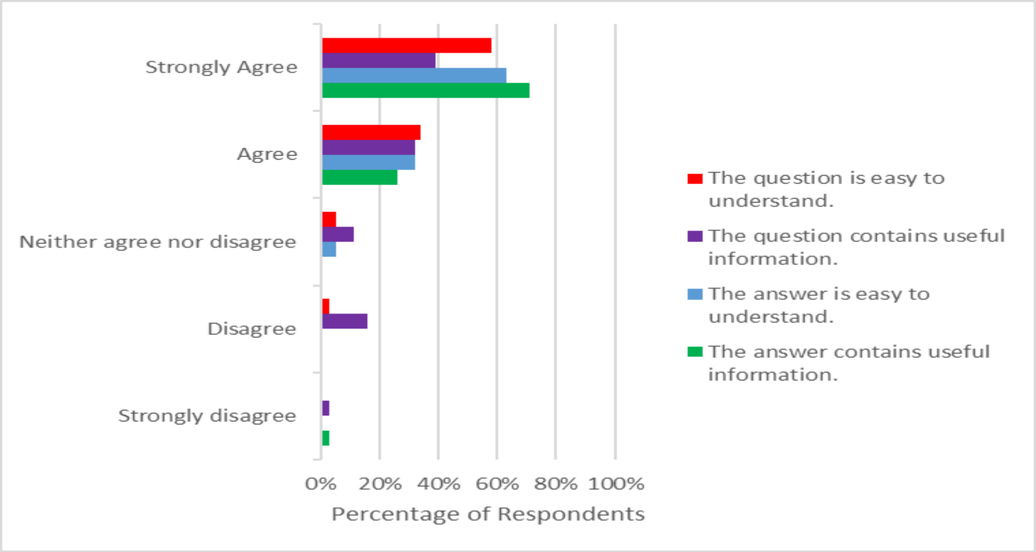

4. If a researcher sees my name in the data when matching, how much will they know about me? If a researcher sees your name in the data, they might see some more identifiers about you (e.g., birth dates, identification numbers, race, gender). But they will not see other health details, such as your diagnosis, health status, blood pressure, etc. Your name is an identifier. We only use identifiers during the patient-matching step of the research to help link records from different databases. In this step other details about you, such as your health information, are not used to match records. We only use this health-related data once we remove your identifiers to protect your privacy.

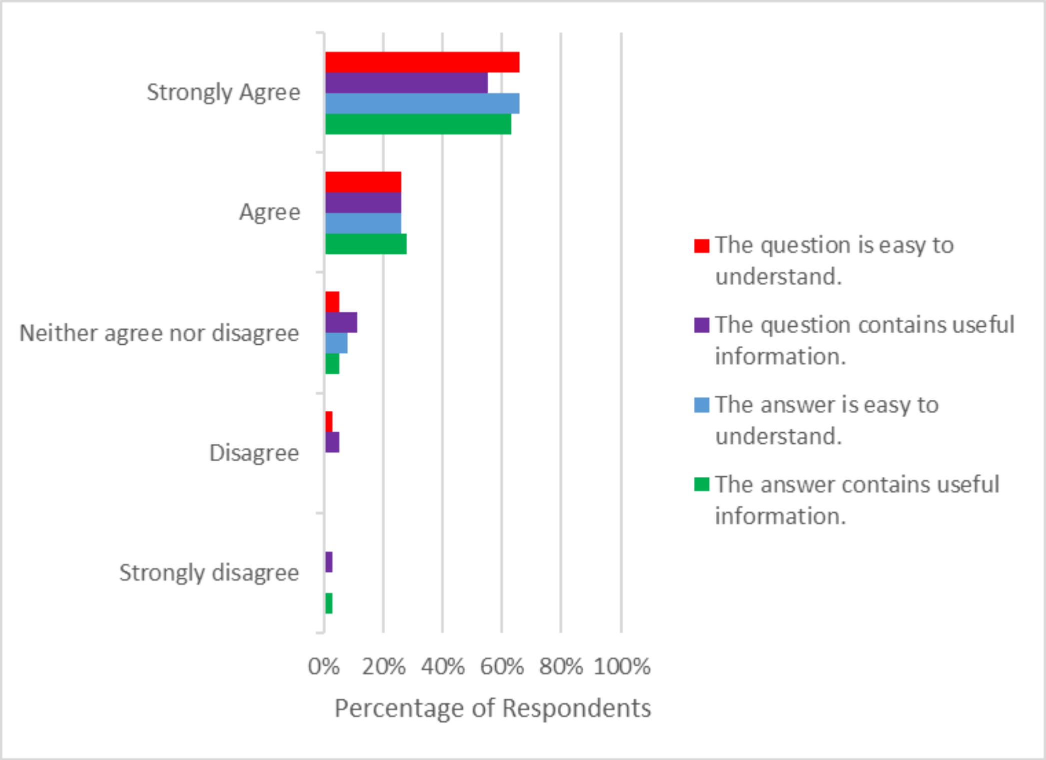

5. What does MINDFIRL look like?
Click below to see a video of MINDFIRL and try it. Note this only works on a computer (not your phone) and you have to use Chrome.

http://mindfil4.herokuapp.com/tutorial/clickable/demo

Was the video helpful?

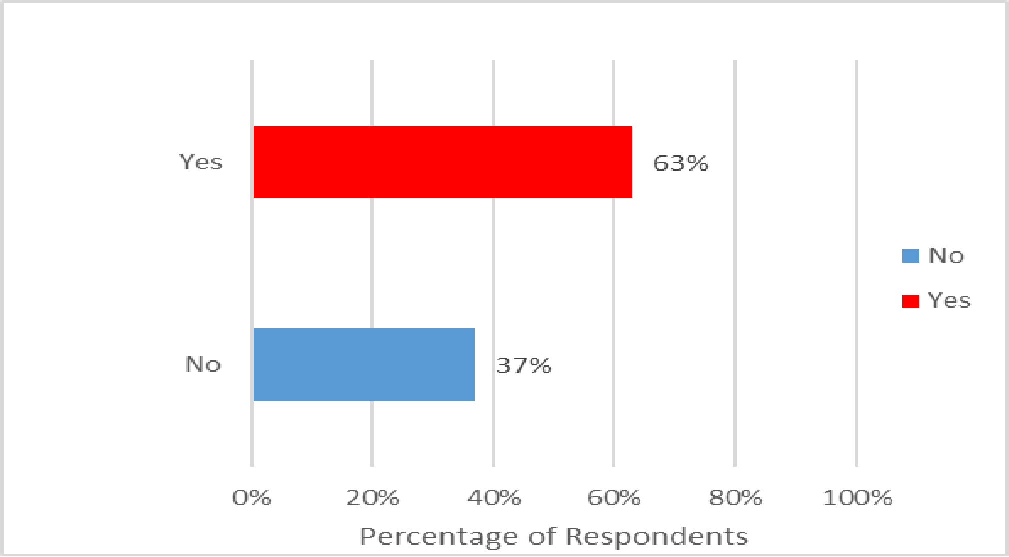

Was the interactive demonstration helpful?

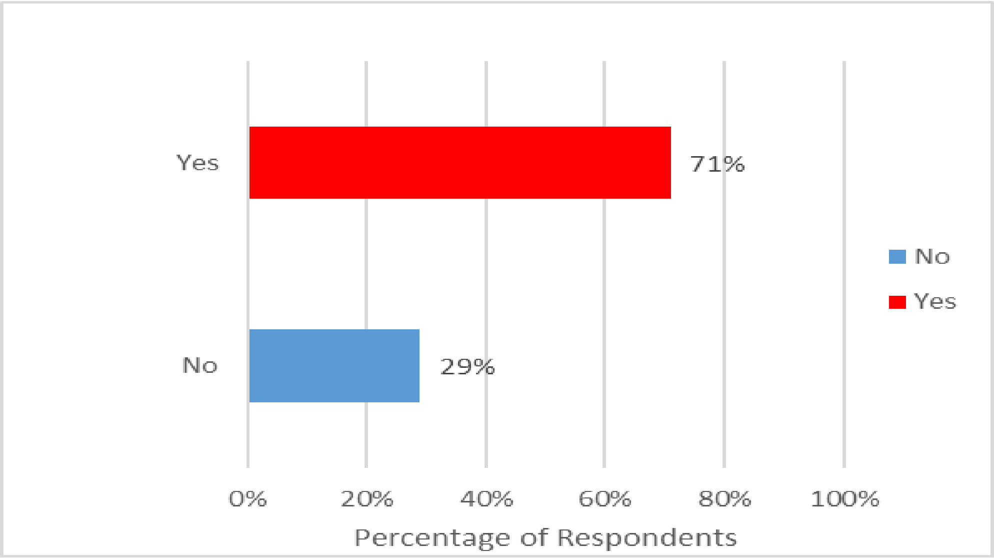

6. What information do you need in order to match my records?
For some people, very few identifiers are needed to match records. For example, records from someone with a very unique name may be easily matched with just a name and perhaps their date of birth. In contrast, the records for someone with a common name may be harder for the researchers to accurately match. As a result, additional information such as gender, race or ethnicity, and identification numbers may be needed.

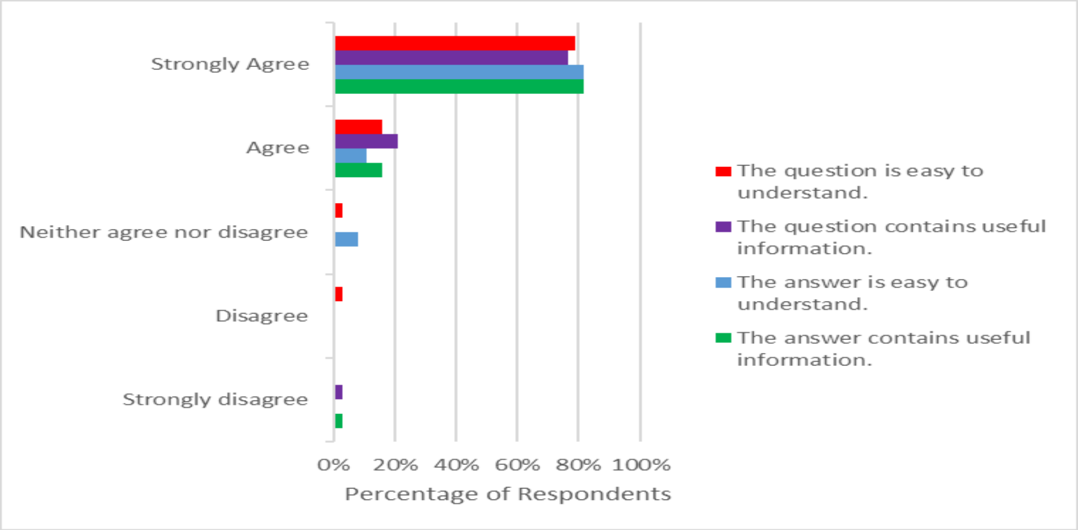

7. Can I be identified in the linked data?
In the age of big data, it is almost impossible to make a dataset fully anonymized and useful at the same time. However, the data we analyze for our research will not contain the identifiers used to match records. Instead, we will remove all identifiers from the matched data and [*Researcher should select* (1) keep them in a separate file, or (2) destroy them] before it is used for analysis.

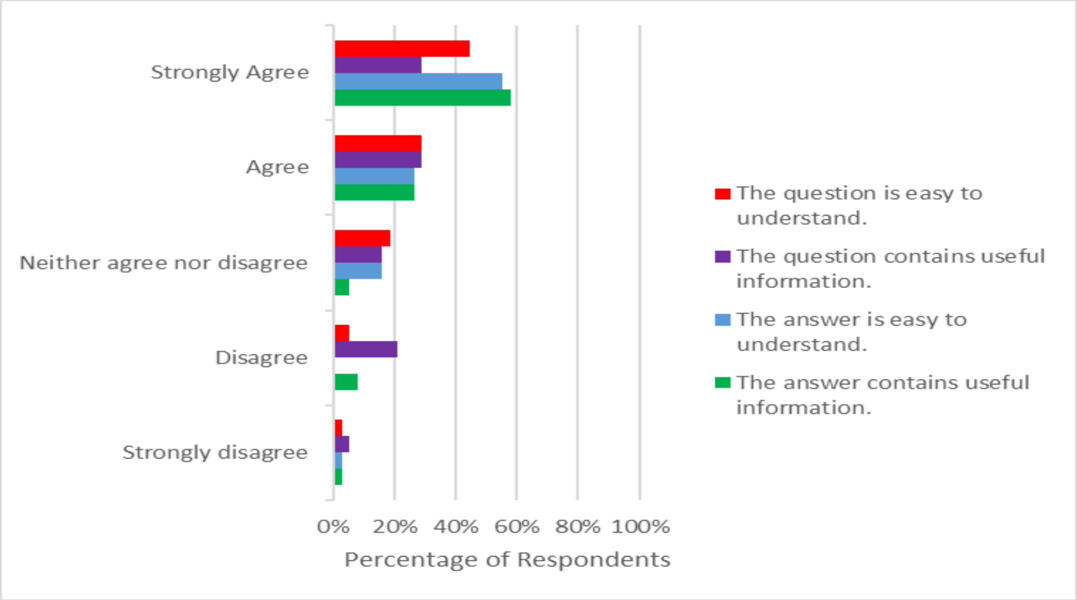

8. Are there risks to patient matching?

Patient matching is not very risky on its own. There is always a slight chance that someone outside our research group may try to gain access to your data or that someone matching the data may misuse it. We work hard to minimize these risks. First, we store all data on secure computers that meet legal standards to minimize the risk of someone breaking into the data. Second, each researcher on this project is trained to use the software properly and comply with the law. These efforts will minimize misuse of the data.

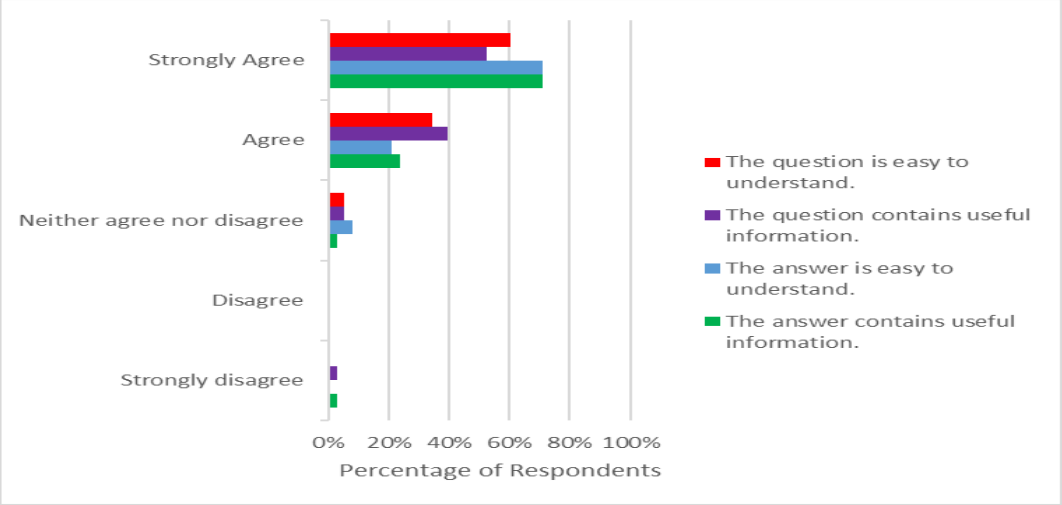

9. Does MINDFIRL reduce risks to patient matching?
Yes, MINDFIRL is designed to improve privacy in database studies. Thus, we expect that MINDFIRL will lower the risk of individual studies. MINDFIRL includes tools to promote transparency and researcher accountability to limit risk. For example, MINDFIRL tracks what identifiers are viewed and who viewed them. This information is used to discourage the misuse of your information. It also allows for setting hard limits on how much data is used. See figure below.

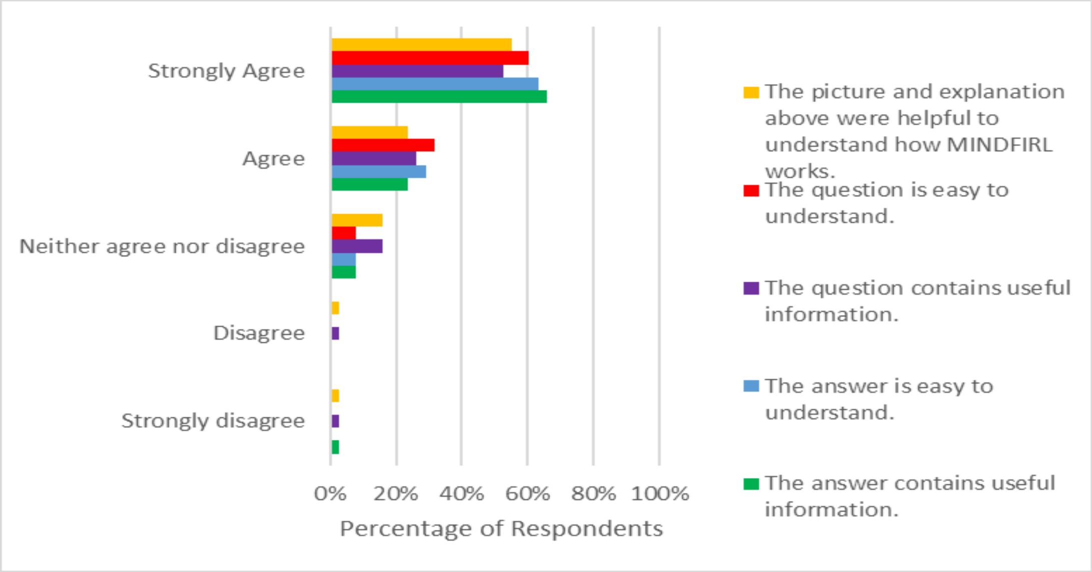

10. How does MINDFIRL enhance privacy?
MINDFIRL improves privacy by greatly reducing the identifying information viewed by researchers. MINDFIRL only shows identifiers if a researcher thinks they need it to match patient records accurately. In addition, MINDFIRL gives researchers clues to help researchers understand

if two masked identifiers are the same, similar, or different. This helps researchers match records without revealing identifying information.

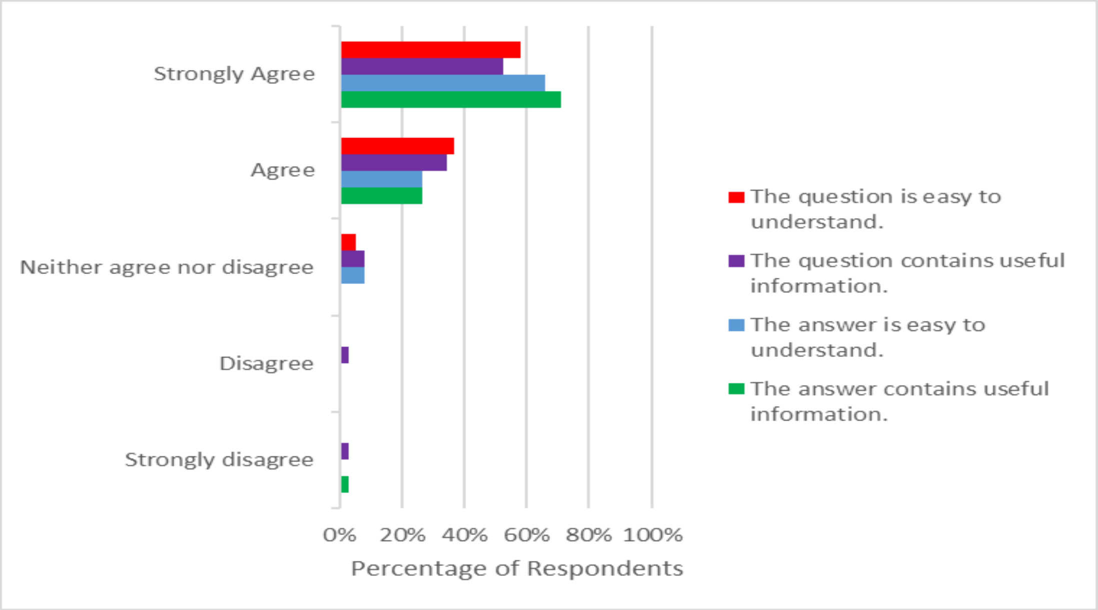

11. Does MINDFIRL reduce the quality of matched records?
No. One study showed that people who used an early version of MINDFIRL were just as accurate as people who saw 100% of the identifiers. However, the people who used MINDFIRL only saw 7% of the identifiers.

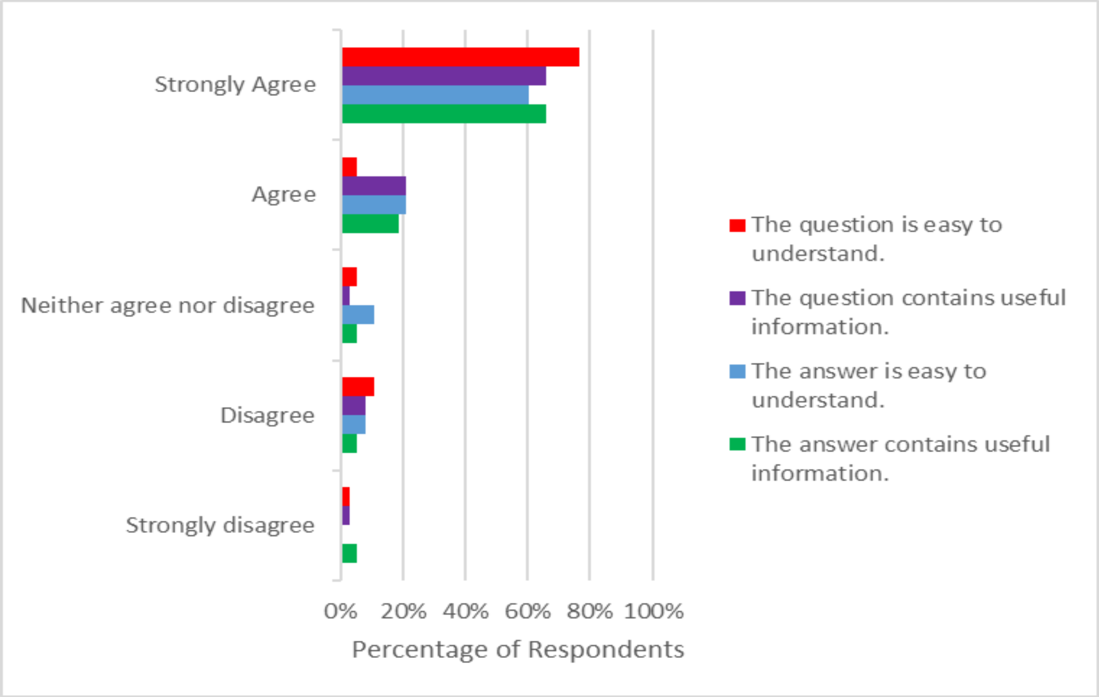

12. Where will data about me be kept?

   a. Identifiers

   Your identifying information will be kept… [This information will vary depending on the specific research project and protocol. Researchers will describe how they will store and protect information used for the study]

   One example response might be: All research data is stored in secure server at Texas A&M University. The Texas A&M University Information Technology (IT) department maintains the servers. See the next section on how the data is secured on this server. Your identifiers will be kept in a separate secure location with different access controls (e.g., password protection). Only the staff involved with patient matching will have access to this folder.

   b. Non-identifiers

   Your non-identifying information such as diagnosis type, medications, etc., will be kept…[This information will vary depending on the specific research project and protocol. Researchers will describe how they will store and protect information used for the study]

   One example response might be: The non-identifiers is stored in the same secured server as the identifiers. However, the non-identifiers will be stored in a different secure folder from the identifiers. Only staff conducting analysis with the data will have access to the folder with the non-identifying data.

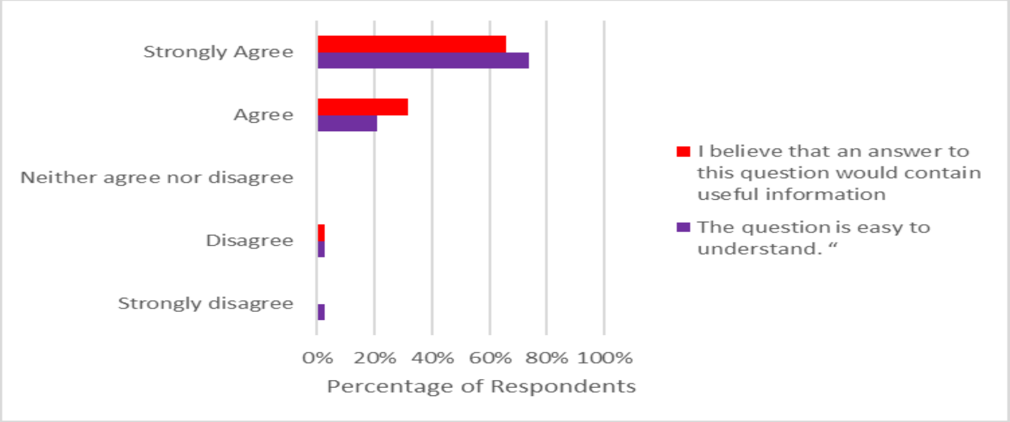

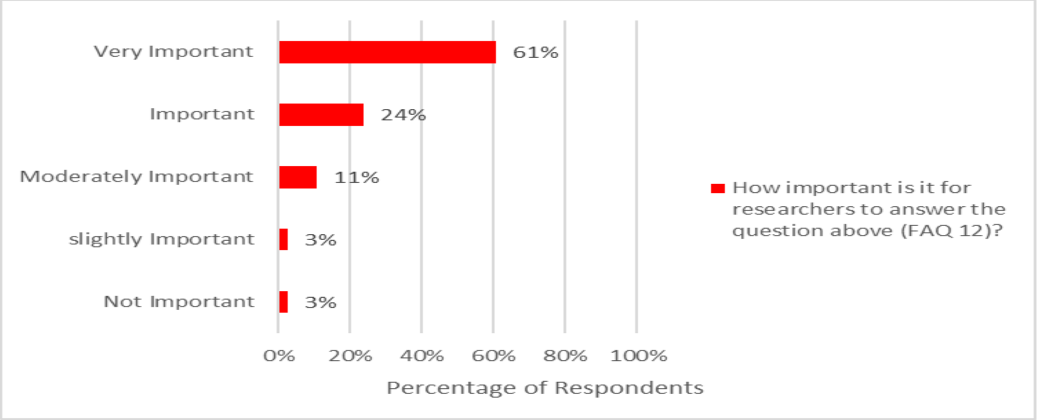

13. What security measures are you using to protect my data?

[This information will vary depending on the specific research project and protocol. Researchers will describe the specific security measures for the study]

Some possible answers might be:

- The server is located in the secure facility with 24/7 monitoring
- Dual authentication (For example, a password and a code sent to a researcher's phone)
- Firewall
- Virtual Private Network (VPN) encrypted connections
- No data is allowed to be taken off the server
- Identifiers are stored separately from non-identifiers
- Using MINDFIRL to reduce access to identifiers
- Security audits for compliance.

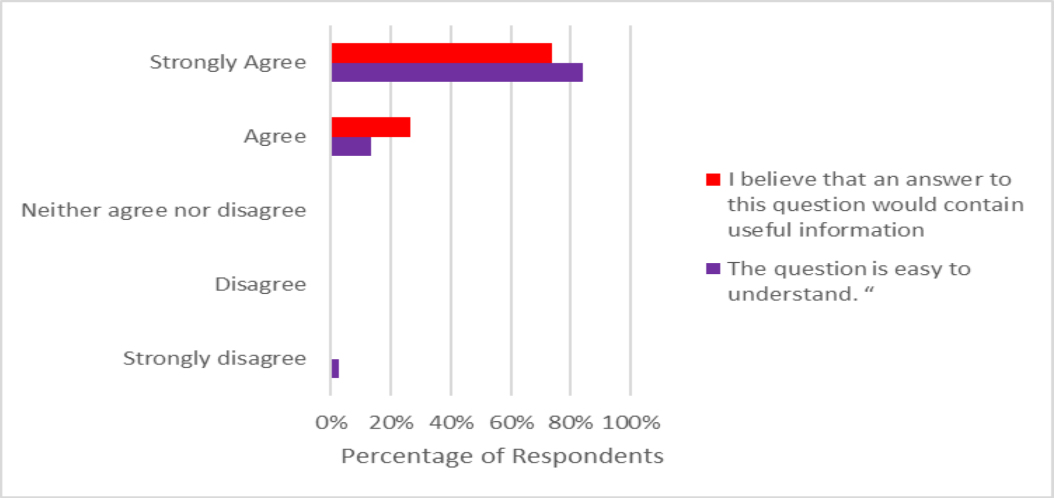

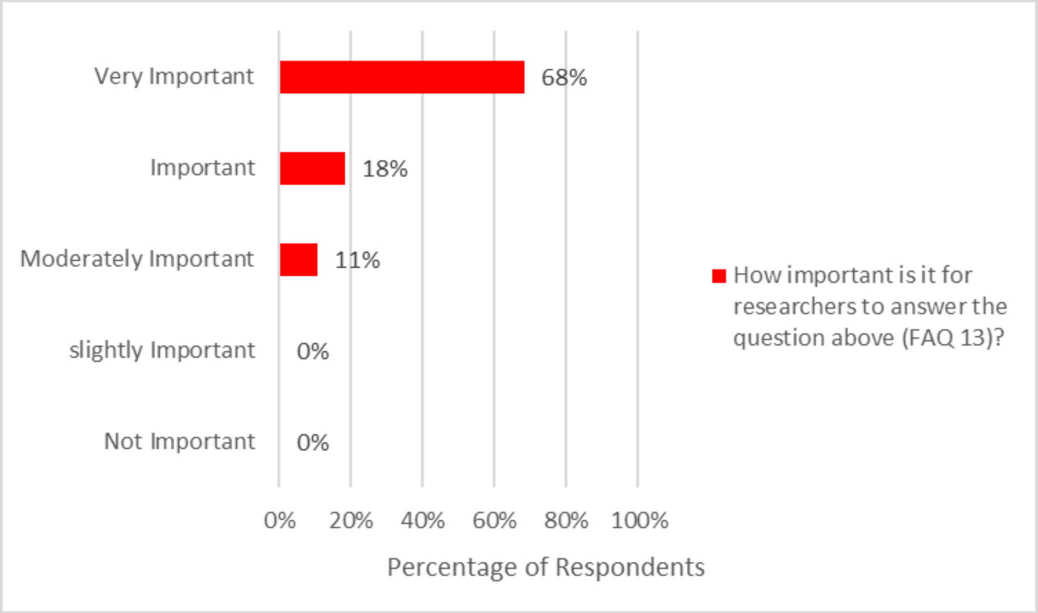

14. What are you doing to make sure that my data is being used responsibly?

This research was reviewed by the institutional review board (IRB) at [Researchers will fill in their IRB information]. The IRB oversees research to make sure it is legally and ethically permissible. We are also using the MINDFIRL software for record linkage to limit access to information that can identify you. This is part of our commitment to conducting responsible research. Furthermore, … [This information will vary depending on the specific research project, protocol, and institutional policies. MINDFIRL allows researchers to customize settings for transparency and accountability. Researchers will describe the specific safeguards that are in place to ensure responsible data use, including policies, MINDFIRL settings, and required trainings.]

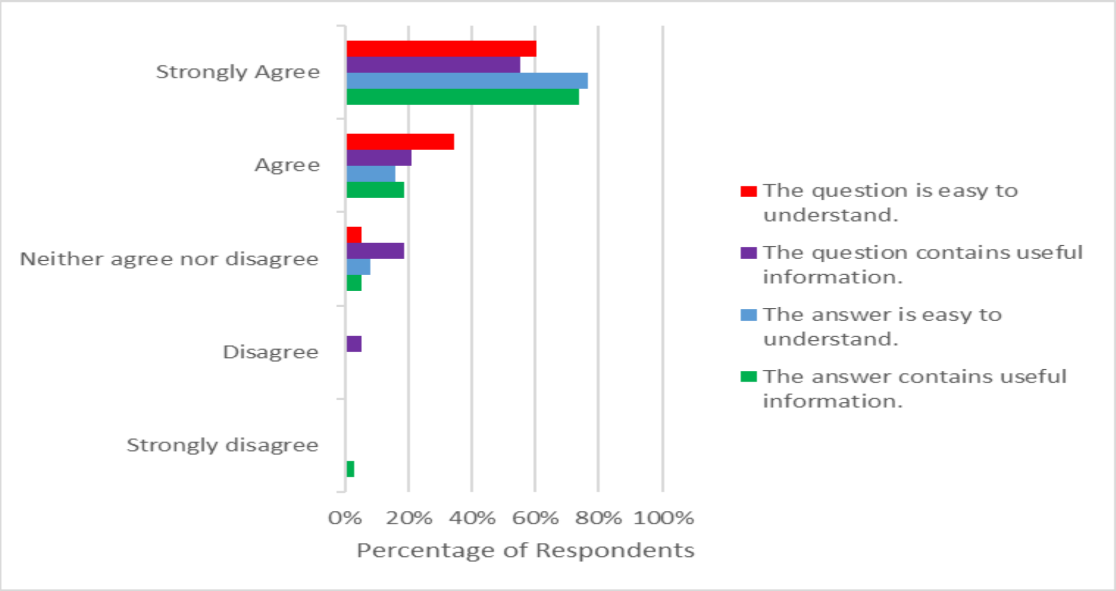

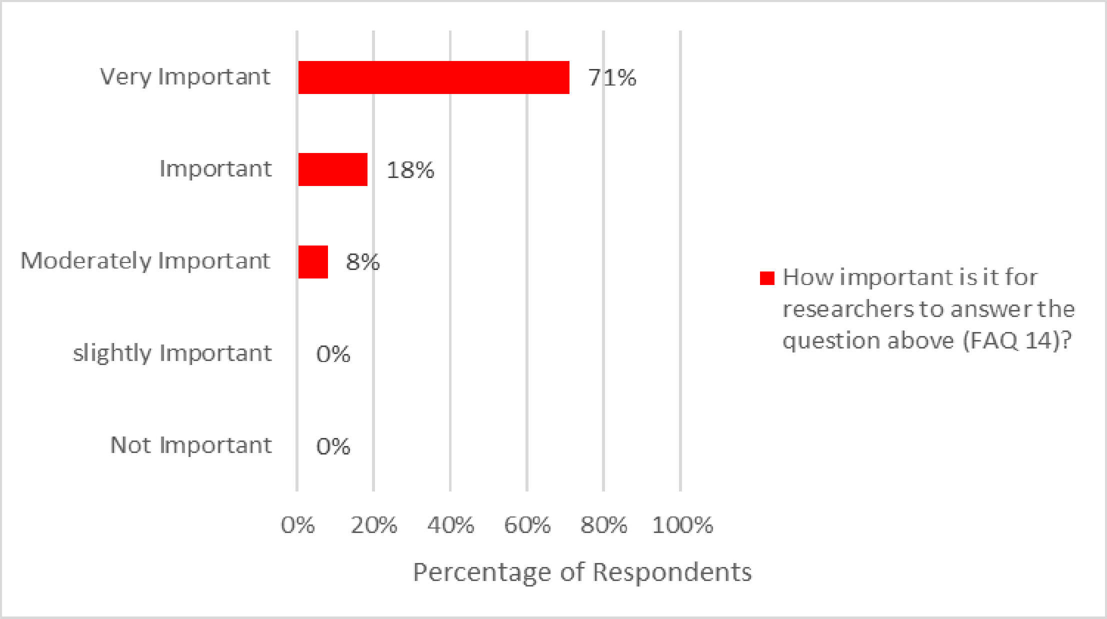

15. What will you do if you discover that my data has been misused?
While we take great measures to safeguard your data, if a data breach were to occur, we would follow legal guidelines for breach notification.

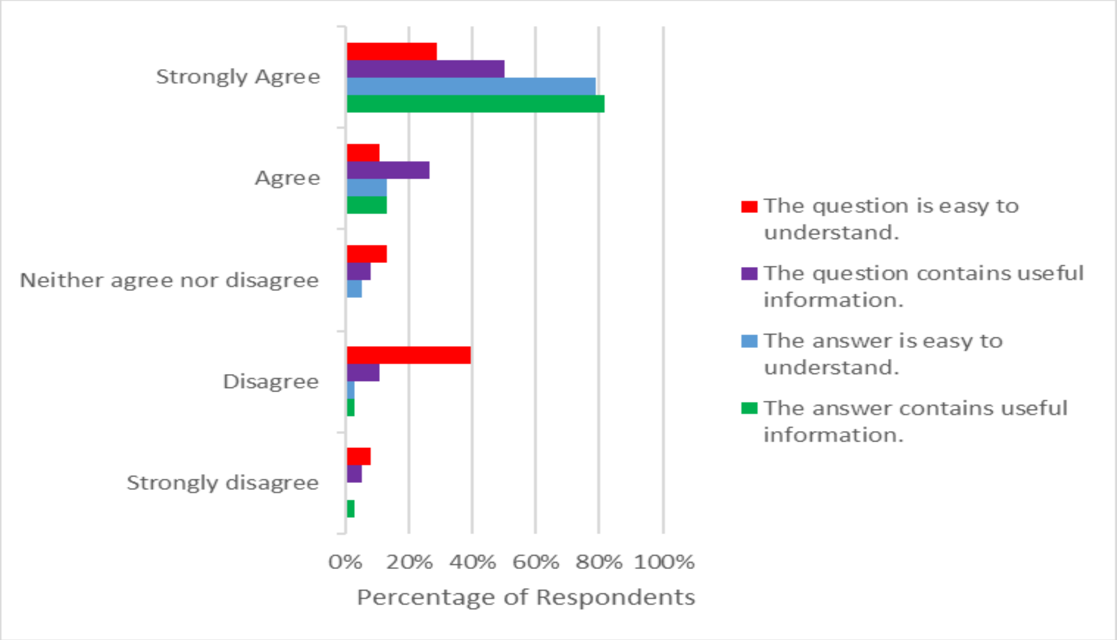

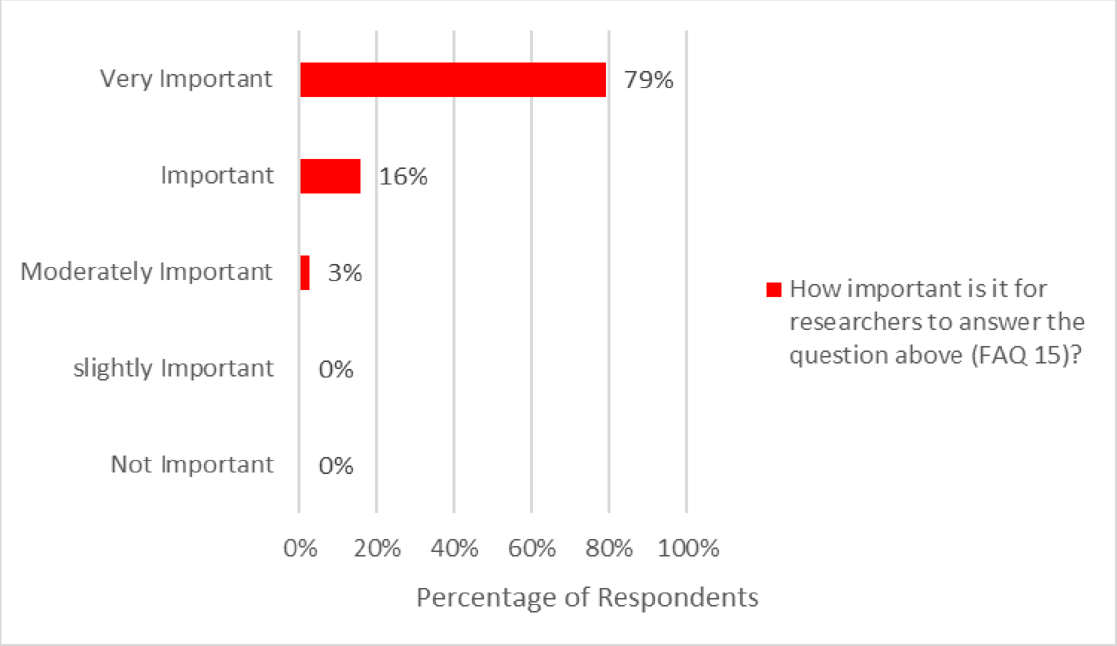

16. Who will have access to my data?
[This information will vary depending on the specific research project. Researchers will describe the qualifications for authorized data users and should consider identifying the research team.]

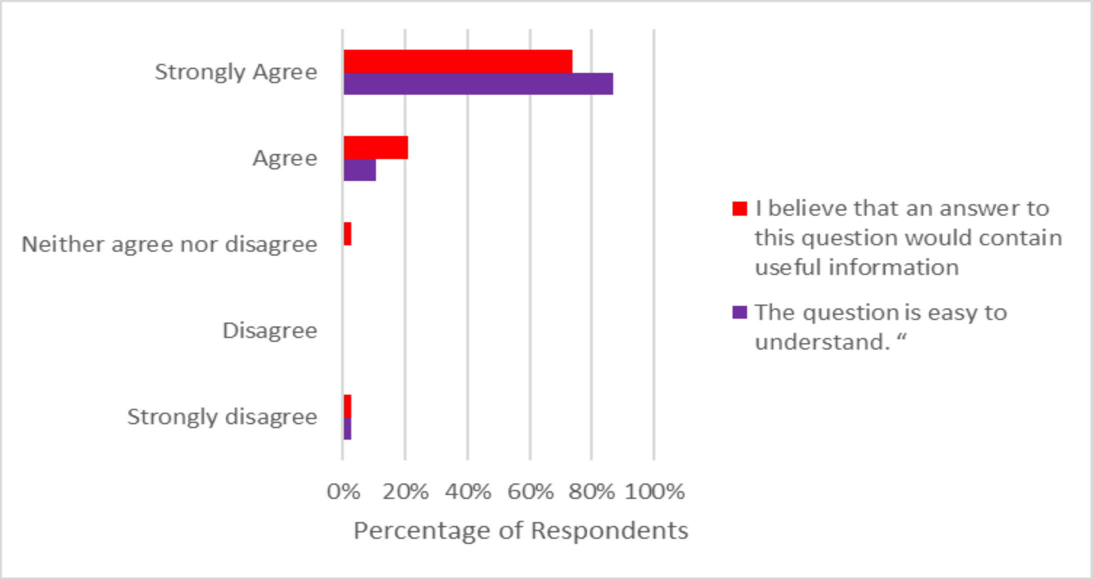

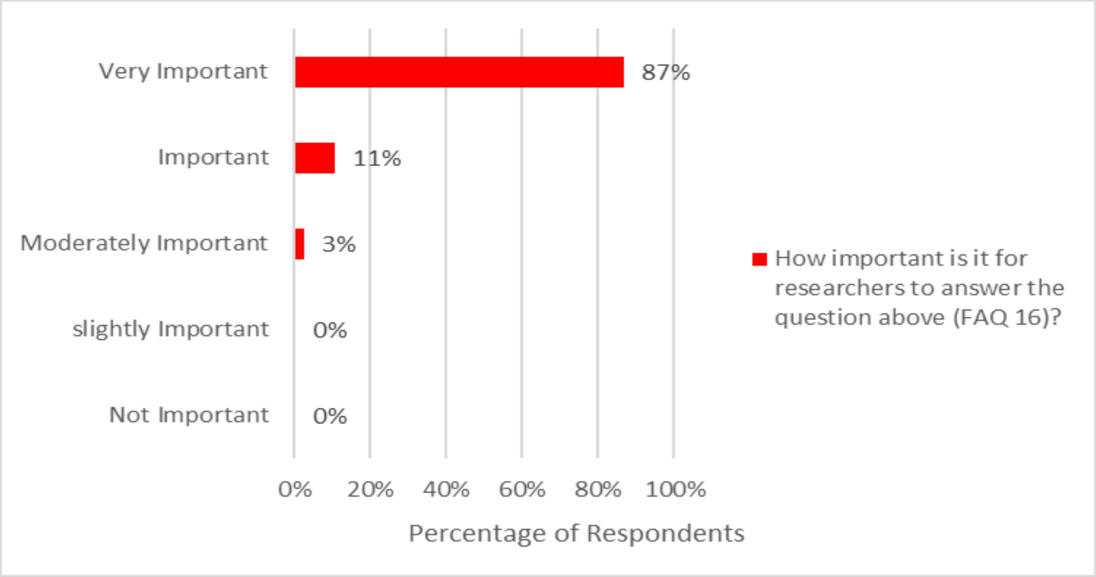

17. Why is my data needed?

Your data is needed so that we can better understand … [This information will vary depending on the specific research project. Researchers will describe the specific research question and provide enough context for readers to understand the problem the research is trying to address.]

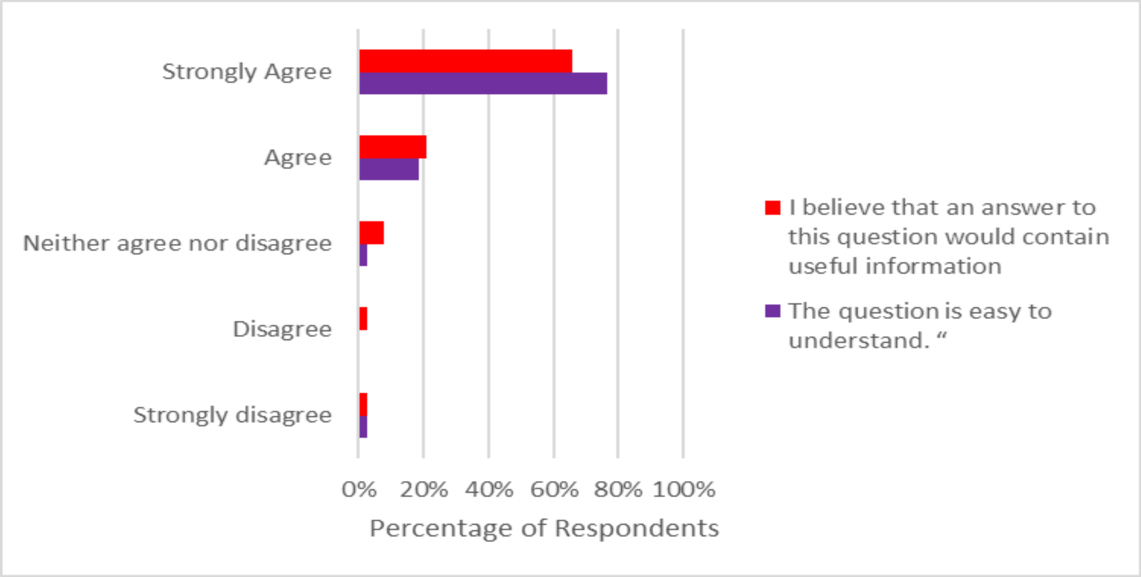

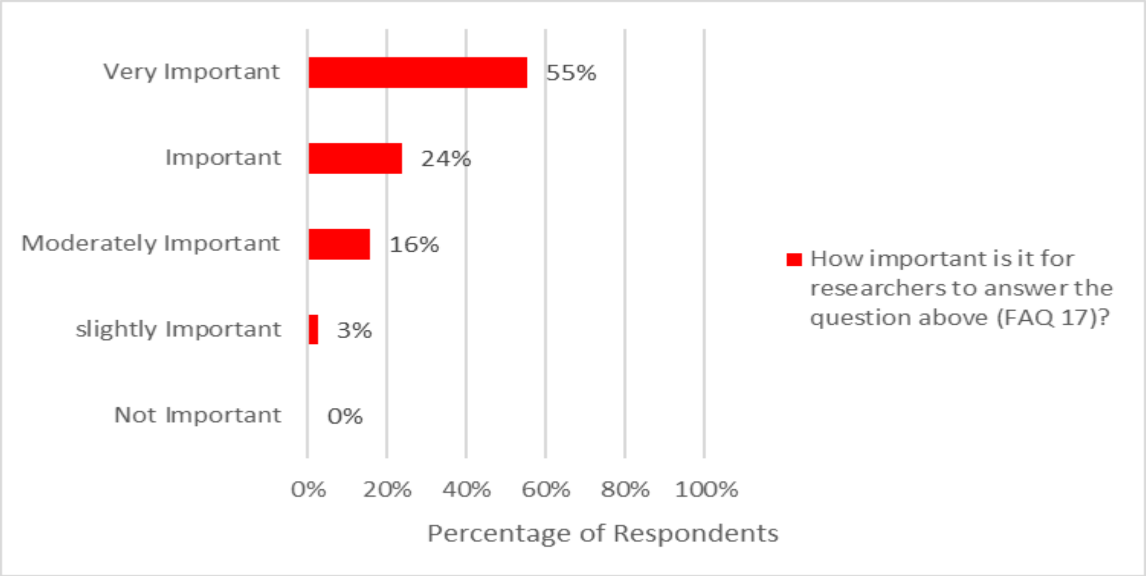

18. How is my data contributing to or advancing science?

[This information will vary depending on the specific research project. Researchers will try to help readers understand how their research contributes to addressing a specific problem. For example, "Your data is advancing our knowledge of problem A in that it helps us determine associations between X and Y."]

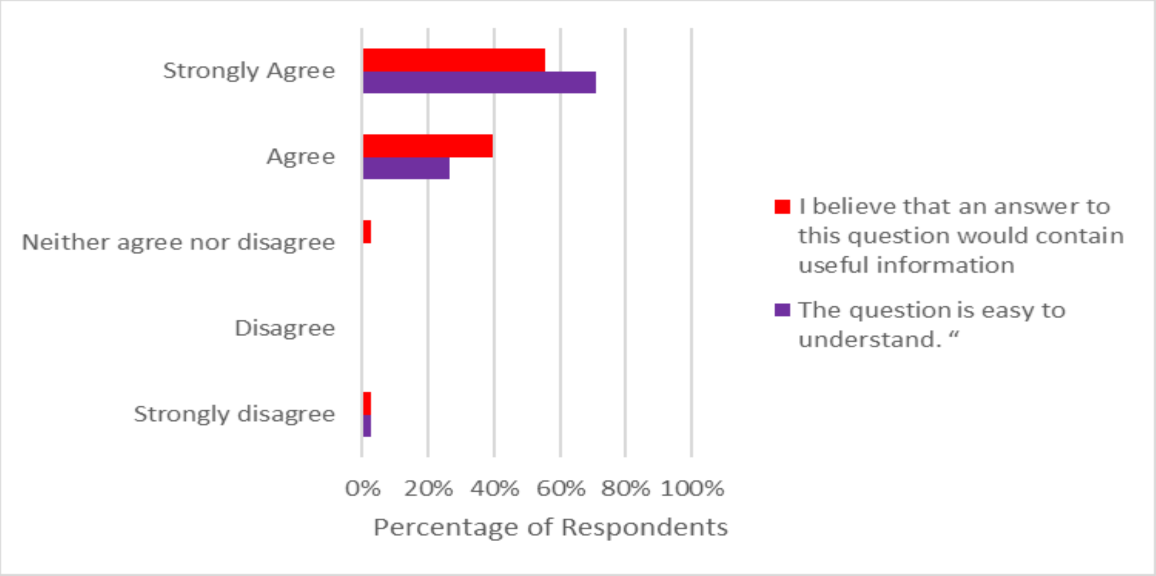

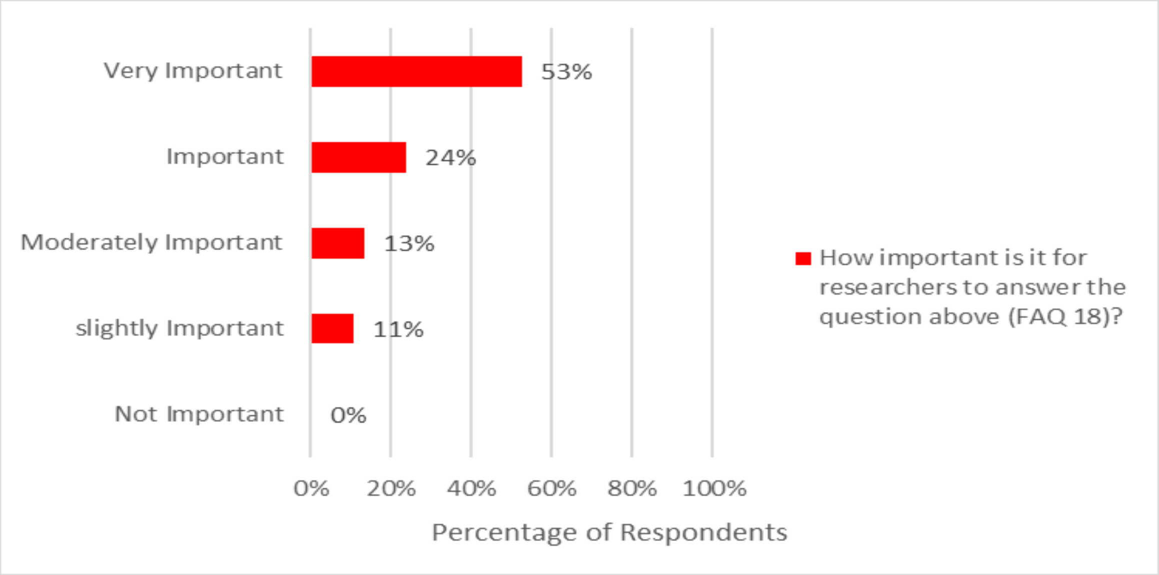

19. What difference is my data going to make?

In research, we use information about a group of people, called a "sample," to understand things about a larger group or "population." If the sample is too different from the larger population then we cannot learn very much from the research. If people like you are not included in the research, then what we learn will not be useful to you or others like you. For example, if young adults are excluded from all studies about drug safety, it will be difficult to ever know if any drugs are safe to use on young adults.

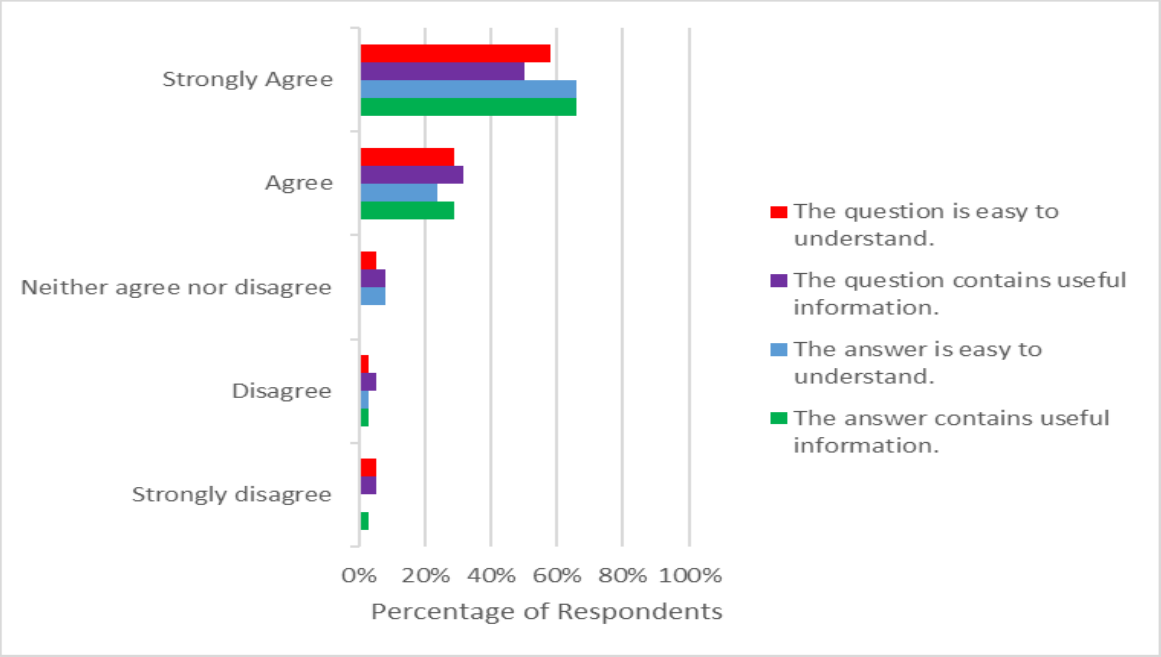

20. What will happen to my data after this study is completed?
[This information will vary depending on the specific research project. Researchers will discuss their plans to destroy, store, or reuse the study data.]

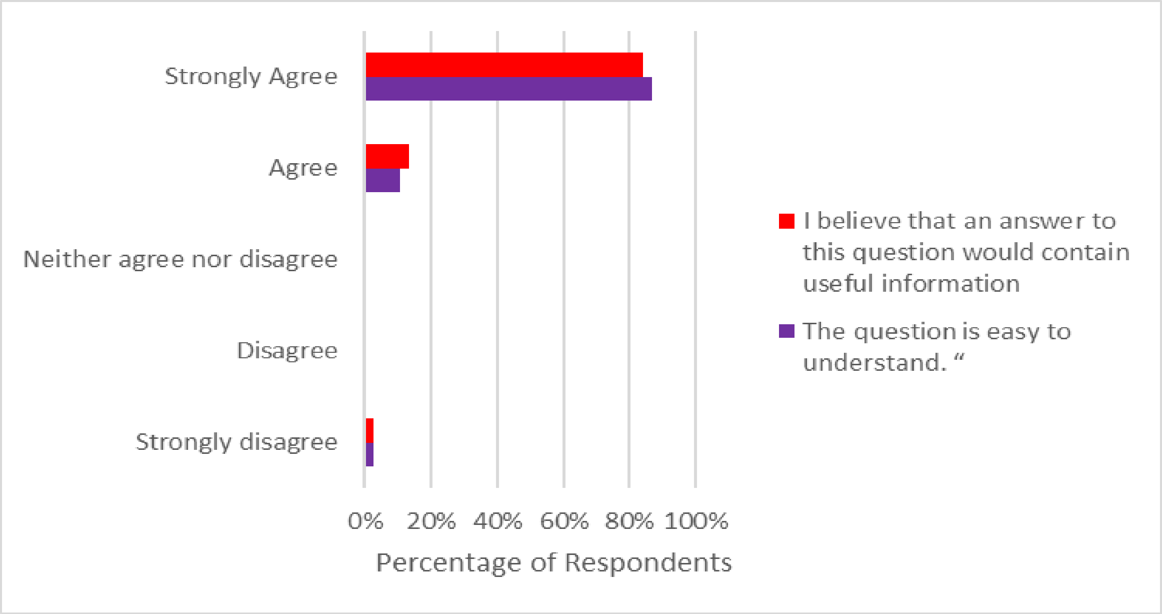

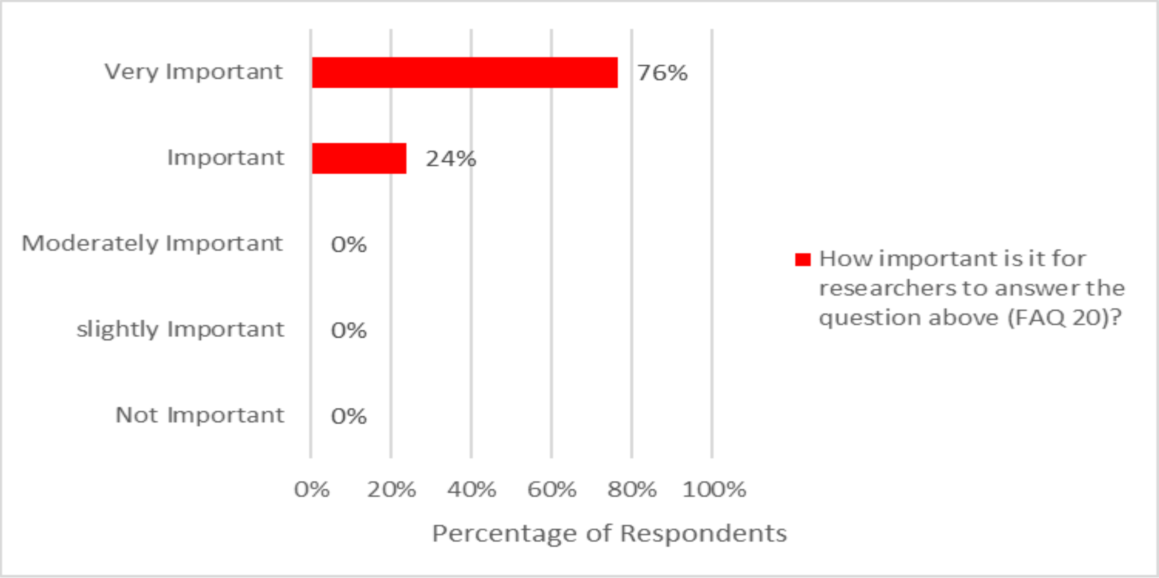

21. Will my matched data be used for other research studies?
[This information will vary depending on the specific research project. Researchers will discuss any plans to reuse the study data.]

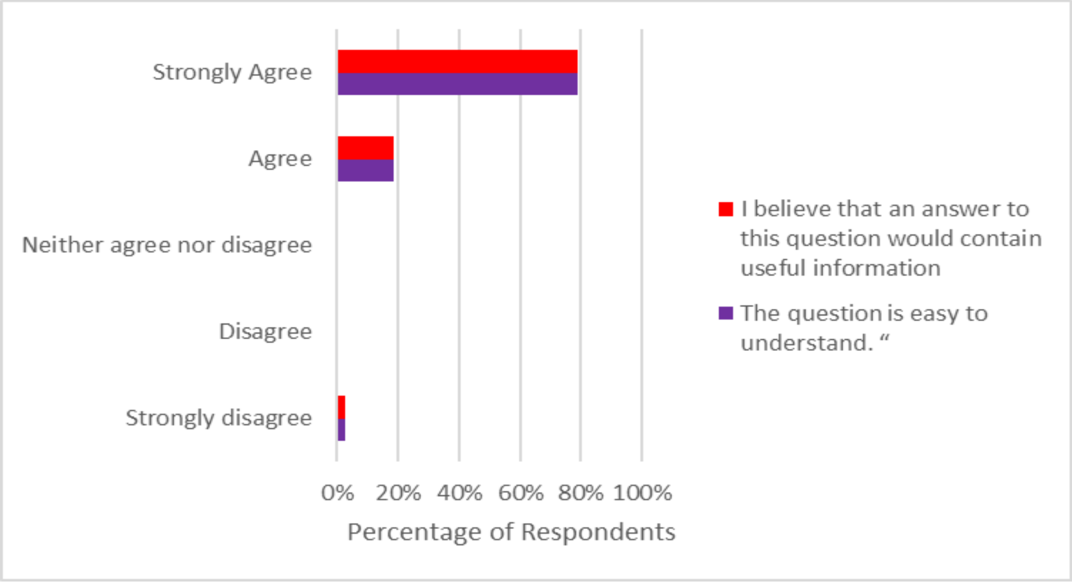

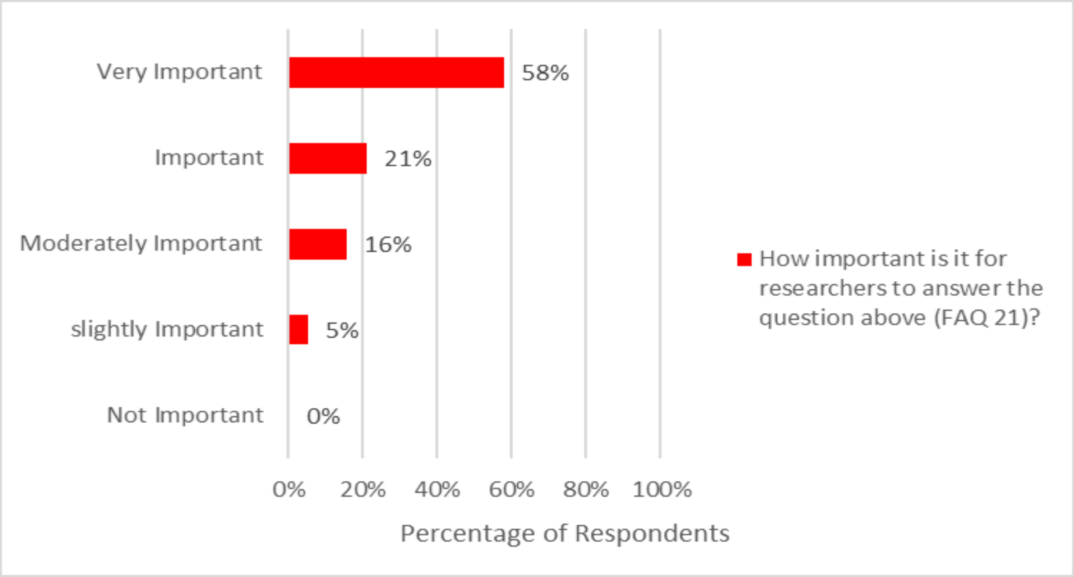

22. Where can I get more information?
    [This information will vary depending on the specific research project. Researchers provide contact information.]

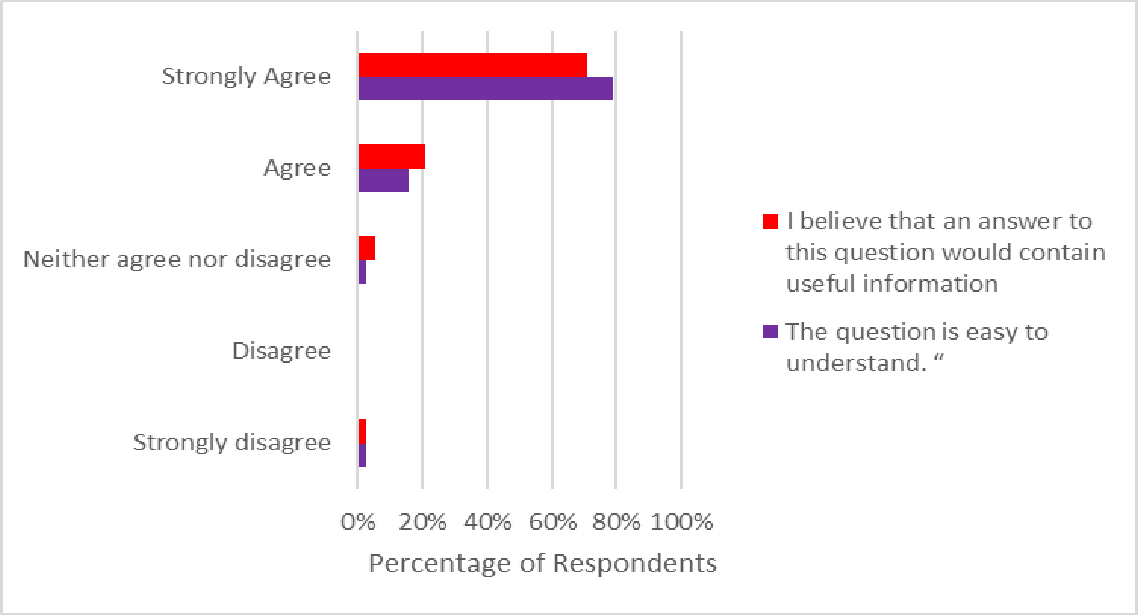

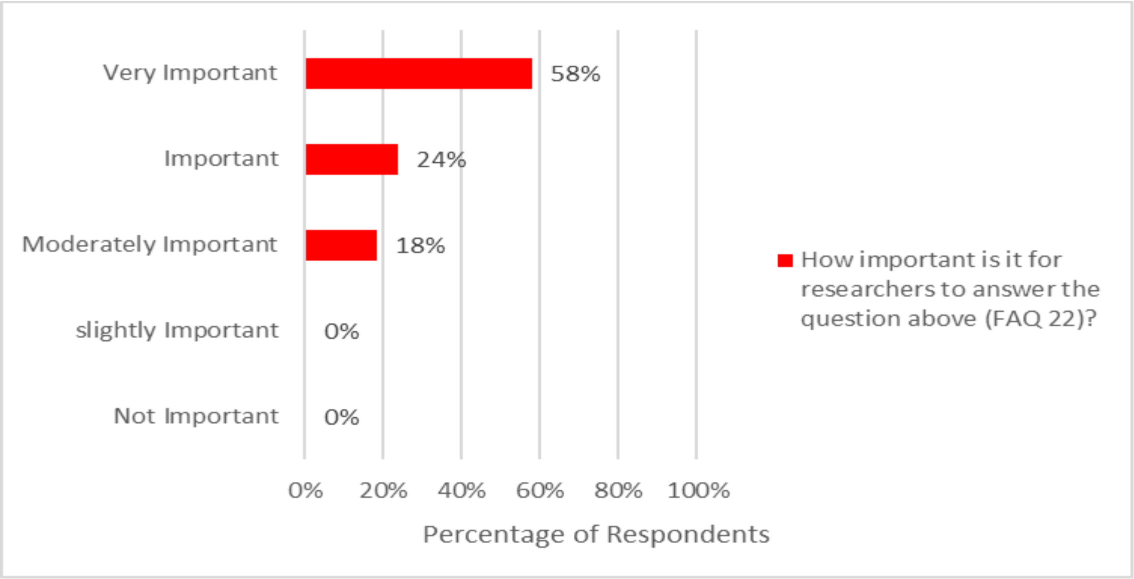
Very Important
58%
Important
24%
Moderately Important
18%
slightly Important
0%
Not Important
0%
0%
20%
40%
60%
80%
100%
Percentage of Respondents
How important is it for researchers to answer the question above (FAQ 22)?
