# Supplementary material for: Communicating With Patients About Software for Enhancing Privacy in Secondary Database Research Involving Record Linkage: Delphi Study
Source: J Med Internet Res. 2020 Dec 15;22(12):e20783. doi: 10.2196/20783 (PMC7772068; doi:10.2196/20783)
Supplement: Multimedia Appendix 3 [file jmir_v22i12e20783_app3.pdf]

This is a Multimedia Appendix to a full manuscript published in the J Med Internet Res.
For full copyright and citation information see http://dx.doi.org/10.2196/jmir.20783

Round 2 Overview

Round 2 Overview

Thirty-eight [38] participants responded to the Round 1 survey with very informative feedback. Thank you! We revised the FAQ in response to this valuable feedback. In this second round of the Delphi, we would like your help to continue to improve the FAQ.

- You can find the revised Frequently Asked Questions (FAQ) document here in full. Please print or keep the document open on a separate screen, and review as you respond to the survey questions.

- There are five sections in the Round 2 survey as outlined below:
    - SECTION 1: General Questions about the FAQ.
    - SECTION 2: Revisions to some specific FAQs. We will provide summary responses, and revised text for you to share your input.
    - SECTION 3: Order of the sections of the FAQ.
    - SECTION 4: Your thoughts about risk in database-only research.
    - SECTION 5: Your thoughts on the Revised FAQ Document.

Section 1: General Questions about FAQ

SECTION 1: General Questions about the FAQ

A number of Round 1 participants suggested that the terms “identifiers” and “non-identifiers” were not ideal for the patient community. These terms were

used in section 2.1.3 in the FAQ. We would like your help in identifying terms that would be easier to understand.

Q1.1 Please indicate the term that you prefer the most for "identifiers":

Identifiers

Identifying information

Identifiable information

Information that can be used to identify individuals

Q1.2 Please list other any other terms that would be more appropriate for *information that can be used to identify individuals*.
(If you have no comments, please type in NA)

Q1.3 Please indicate the term that you prefer the most for "non-identifiers":

Non-identifiers

Non-Identifying information

Non identifiable information

Health-related study data

Q1.4 Please list any other terms that would be more appropriate for *the information used in research that does not contain identifiers*.
(If you have no comments, please type in NA)

A number of participants wanted more information relating to the "legal guidelines" mentioned in the following questions and answer.

2.3.15 What will you do if you discover that an unauthorized person has accessed my data or my data has been otherwise misused?

While we take great measures to safeguard your data, if a data breach were to occur, we would follow legal guidelines for breach notification.

This is a difficult FAQ question to answer in general language because there are many different privacy laws that could apply to different research projects. We would like your help in identifying a better answer for (a) patients reading this FAQ and (b) researchers who want to adapt and use this FAQ for their projects.

Please respond to the following statements:

	Strongly Agree	Agree	Neither agree nor disagree	Disagree	Strongly disagree
Q1.5	○	○	○	○	○

	Strongly Agree	Agree	Neither agree nor disagree	Disagree	Strongly disagree
Including internet links to the legal requirements is important for this answer					
Q1.6 Including internet links to any organizational rules is important for this answer	○	○	○	○	○
Q1.7 The researchers should summarize any legal breach notification requirements	○	○	○	○	○
Q1.8 The researchers should summarize any organizational breach notification requirements	○	○	○	○	○
Q1.9 The researchers should summarize any breach notification processes required by the Institutional Review Board (IRB)	○	○	○	○	○

Section 2 Revision of selected FAQ's

SECTION 2: Revisions to selected FAQ’s

Based on your responses in Round 1, there were five questions and accompanying template responses that were particularly problematic, and two additional questions and accompanying template language that were somewhat problematic. We revised these questions below with "tracked changes" (see **green** text). In addition, we have added a few more FAQ items based on your responses as well.

In this section, we will ask you to focus on some of these revised questions and give us your feedback. For each question that you saw previously in Round 1,

we provide a summary of the responses in text, followed by a screenshot of the revised language with tracked changes.

Note: Asterisks indicate magnitude of negative feedback. Questions with three asterisks (***) were particularly problematic, and questions with two (**) were somewhat problematic.

***2.1.3 What information about me will the researchers see?
[NEW] 2.2.5.a What is patient-matching?
[NEW] 2.2.5b What is MINDFIRL?
***2.2.7 Can I be identified in the linked data?
**2.2.9 Does MINDFIRL reduce lower the privacy risks of patient matching?
***2.2.11 Does MINDFIRL reduce the quality of matched records?
[NEW] 2.2.12 How can MINDFIRL help patient-matching while hiding identifiers?
**2.3.14 What are you doing to make sure that my data is being used responsibly?
***2.5.18 What difference is my data going to make?

*****2.1.3 What information about me will the researchers see?**

Summary of Responses

Suggestions to improving the response to the question included telling patients that the information that researchers will see is information that healthcare providers already have collected on their patients during previous visits and a sense of where the data is pulled from. Additionally, one participant suggested that a statement on whether the persons doing the linkage and the persons doing the research are the same. In addition, it was suggested that a statement on whether the general release of information that patients sign when seeking care is what allows the use of patient data for the research study.

Several participants indicated that the reading/required literacy level for the response as it stands is too high, and that words such as ‘identifying information’

and ‘non-identifying information’ would be more appropriate instead of ‘identifiers’ and ‘non-identifiers.’

Based on this feedback, we have edited the response to 2.1.3 as seen here:

2.1.3 What information about me will the researchers see? [Note: we will revise identifier/non-identifier terminology based on survey feedback in this round]

We~~The researchers~~ need different information for different steps of the research process. We only need identifiers to do patient matching. Additionally, we only need non-identifiers when we are using your health related data to learn more about science or medicine. We will use a software program called MINDFIRL (MINimum Necessary Disclosure For Interactive Record Linkage) to keep identifiers separate from non-identifiers and ensure that no one can access identifiers and non-identifiers together at the same time. In many cases, patient matching is done all with a linkage software, like MINDFIRL, and a person may never see your identifiers because the computer is automatically matching patients without human effort. For others records where the computer is not sure, researchers are asked to determine the match manually.

a. Who will be able to see the i~~I~~dentifiers?

The researchers that will be doing the record matching will have access to identifiers. Information such as your name, date of birth, marital status, and gender help distinguish you from other people. Our r~~R~~esearchers need to access identifiers to match patient records.

We are using the MINDFIRL software to protect identifiers and prevent unnecessary privacy loss during this process. First, MINDFIRL separates identifiers from the non-identifiers. This means that, no one can access the identifiers AND the health-related data at the same time. ~~For example~~Second, MINDFIRL tells researchers when two records have the same identifiers without showing details. In these cases, our researchers might not need to see specific identifiers to make a match. MINDFIRL also tells researcher when records are highly similar without showing details. MINDFIRL only shows identifiers on an ‘as needed’ basis. For example, a researcher might want to see some details to know if a difference is important (e.g, to tell twins apart). This means that MINDFIRL can help catch common matching problems, such as nicknames (e.g., Pam v. Pamela) or typos (e.g., John v. Jonh), without showing the rest of your identifiers.

b. Who will be able to see the Non-identifiers or~~,~~ health-related study data?

Non-identifiers are everything else in the data. Non-identifiers could include information such as diagnosis, medications, or blood pressure. ~~We~~ Our researchers will only use the non-identifiers for the main research after the matching is done. In some cases, the same researchers who match the records will use the non-identifiers for the main research. However, ~~After matching records,~~ MINDFIRL separates identifiers from the non-identifiers. This means that~~Thus~~, no one can access the identifiers AND the health-related data at the same time. We will code your non-identifiers to protect your identity. This allows us to use your information to make scientific or medical discoveries without knowing which information belongs to you.

Please tell us what you think about the following statements:

	Strongly agree	Somewhat agree	Neither agree nor disagree	Disagree	Strongly disagree
The question is easy to understand.	O	O	O	O	O
An answer to this question is important to me.	O	O	O	O	O
The answer is easy to understand.	O	O	O	O	O
The answer contains useful information.	O	O	O	O	O

Do you have any thoughts or comments relating to the updated response above?
(If you have no comments, please type in NA)

[NEW] 2.2.5.a What is patient-matching?

[NEW] 2.2.5.a What is patient-matching?

Patient Matching is the process of linking records of the same real-world person from different databases. Patient matching helps researchers answer difficult questions. For example, is disease treatment A better than treatment B for keeping patients healthy? To do this, we might want to count the number of emergency room visits Jane Doe made this year across several hospitals. This requires linking records from all the hospitals she visited.

This is hard because a universal identification number does not exist to easily link records in different systems. Instead, we have to use the personally identifiable information (PII), like social security numbers (SSNs), first & last names, birthdates, race, and gender to decide which records belong to the same person.

Still, patient matching is hard. Identifiers are not unique. Different people may share the same name. Names are inconsistent (e.g. nicknames). Data is sometimes missing (e.g. SSNs are often missing). Names change over time (e.g., changing a last name at marriage). Data can have errors (e.g. typos). Below is an example of what patient-matching might look like:

	ID	First name	Last name	DoB(M/D/Y)	Sex	Race
Patient in Database 1 →	8000002767	JUDE	WILLIAM	09/09/1906	M	W
Patient in Database 2 →	8000003567	JUDE	WILLIAM JR	09/09/1960	M	B

Please tell us what you think about the following statements:

	Strongly agree	Somewhat agree	Neither agree nor disagree	Disagree	Strongly disagree
The question is easy to understand.	○	○	○	○	○
An answer to this question is important to me.	○	○	○	○	○
The answer is easy to understand.	○	○	○	○	○
The answer contains useful information.	○	○	○	○	○

Do you have any thoughts or comments relating to the **New question and answer** above?
(If you have no comments, please type in NA)

[NEW] 2.2.5.b What is MINDFIRL?

[NEW] 2.2.5b What is MINDFIRL?

MINDFIRL (Minimum Necessary Disclosure for Interactive Record Linkage) is a software that we use to help protect privacy in the patient matching process of our research. It protects privacy in a few ways. First, it separates the identifiers from the health-related study information. That way, the researchers can view identifiers to help them accurately match records without seeing sensitive information.

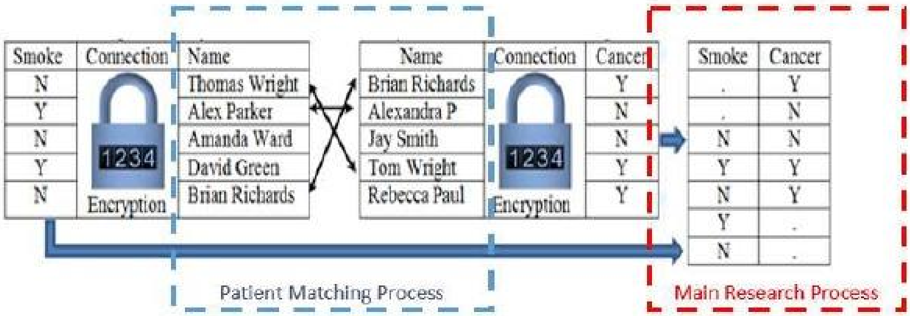

This image shows how MINDFIRL helps the patient-matching process while limiting access to sensitive information. The identifiers are separated from the rest of the information during the patient-matching process. In this way, if a researcher sees someone's identifying information, the researcher cannot see any other sensitive information at the same time.

Second, MINDFIRL hides identifiers, while giving our researchers clues about how similar or different the same identifier is in two records. That way, MINDFIRL allows our researcher to match records without seeing the specific identifiers. See below.

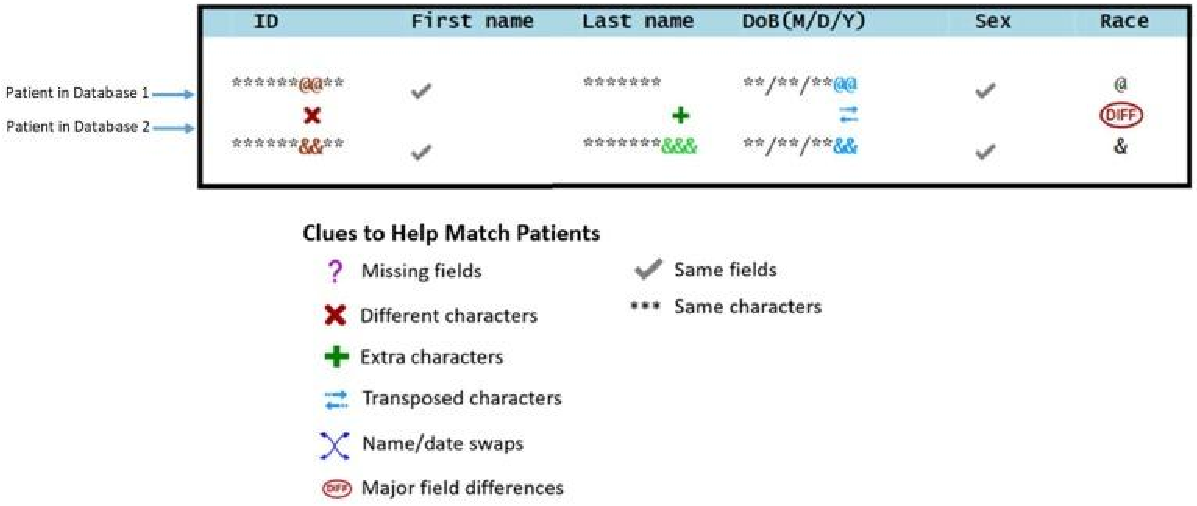

Third, MINDFIRL allows researchers to selectively reveal information to help accurately match patients. This way, a research does not have to see every identifier in order to make a correct match. You can see how this works in this clickable demo (You must use chrome on your PC for this demo to work well. The link will not work on a phone.): http://mindfil4.herokuapp.com/tutorial/clickable/demo.

Finally, MINDFIRL tracks and records the information that researchers see. In this way, the research supervisors can ensure that the researchers who are matching records are not abusing their position by revealing too much.

Please tell us what you think about the following statements:

	Strongly agree	Somewhat agree	Neither agree nor disagree	Disagree	Strongly disagree
The question is easy to understand.	○	○	○	○	○
An answer to this question is important to me.	○	○	○	○	○
The answer is easy to understand.	○	○	○	○	○
The answer contains useful information.	○	○	○	○	○

Do you have any thoughts or comments relating to the **New question and answer** above?
(If you have no comments, please type in NA)

*****2.2.7 Can I be identified in the linked data?**

Summary of Responses

One participant suggested that a definition of 'linked data' be provided for clarity. Another participant indicated that the response is confusing because it states that all identifiers will be removed, but implies that a researcher could click on identifying cells to get additional information for a match decision.

Based on this feedback, we have edited the response to 2.2.7 as seen here:

2.2.7 Can I be identified in the linked data?

It is unlikely, but possible, that you might be identified in the linked data. We remove all identifiers in the linked data, so if someone wanted to identify you in the linked data they would have to use some other information to tell you apart from everyone in the linked data (and the rest of the world). We use encryption and secure computer systems to protect this data to reduce the risk that someone can identify you in the data. ~~In the age of big data, it is almost impossible to make a dataset fully anonymized and useful at the same time. However, the data we analyze for our research will not contain the identifiers used to match records. Instead, we will remove all identifiers from the matched data and [*Researcher should select* (1) keep them in a separate file, or (2) destroy them] before it is used for analysis.~~

Please tell us what you think about the following statements:

	Strongly agree	Somewhat agree	Neither agree nor disagree	Disagree	Strongly disagree
The question is easy to understand.	○	○	○	○	○
An answer to this question is important to me.	○	○	○	○	○
The answer is easy to understand.	○	○	○	○	○
The answer contains useful information.	○	○	○	○	○

Do you have any thoughts or comments relating to the updated response above?
(If you have no comments, please type in NA)

2.2.9 Does MINDFIRL reduce lower the privacy risks of patient matching?

Summary of Responses

A few respondents indicated that the use of the word ‘budget’ in the figure was not ideal. Suggestions for a different word included ‘firm limit’ or ‘cap.’

Based on this feedback, we have edited the response to 2.2.9 as seen here:

2.2.9 Does MINDFIRL ~~reduce~~ lower the privacy risks ~~to~~f patient matching?

Yes, MINDFIRL is designed to improve privacy in database studies. ~~Thus~~As a result, we expect that MINDFIRL will lower the risk of individual studies. MINDFIRL includes tools to promote transparency and monitor researcher ~~accountability~~ activity to limit risk. For example, MINDFIRL tracks what identifiers are viewed and who viewed them. This is similar to a store that has a surveillance camera to make sure that the cashier does not take money from the cash register. This tracking information is used to discourage the misuse of your information. It also allows for setting hard limits on how much data is used. See figure below.

Privacy Meter with Limit

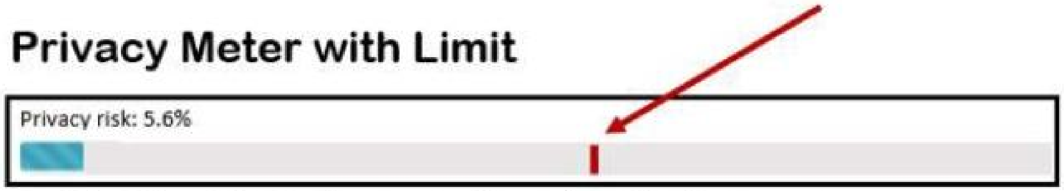

- The Privacy Meter reduces your risk to privacy loss in two ways.
- First, the meter helps the researchers be more aware of the risk of privacy in their work during patient matching. It will also record how much was seen for future audits for better accountability.
- Second, the blue bar indicates how much information in total they have seen so far to do their job. The solid red line is the limit, and represents the **maximum amount of identifying information that can be revealed** during the patient matching process.

Please tell us what you think about the following statements:

	Strongly agree	Somewhat agree	Neither agree nor disagree	Disagree	Strongly disagree
The question is easy to understand.	○	○	○	○	○
An answer to this question is important to me.	○	○	○	○	○
The answer is easy to understand.	○	○	○	○	○
The answer contains useful information.	○	○	○	○	○

Do you have any thoughts or comments relating to the updated response above?
(If you have no comments, please type in NA)

*****2.2.11 Does MINDFIRL reduce the quality of matched records?**

Summary of Responses

One participant provided alternative wording to 2.2.11 to instead ask, “Does using MINDFIRL to use the minimum required identifiers impact the accuracy of patient matching?" Additionally, several participants indicated that the wording of the response was at too high a reading/literacy level and suggested that less technical language be used to answer the question.

Based on this feedback, we have edited the response to 2.2.11 as seen here:

2.2.11 Does MINDFIRL reduce the quality of matched records?

No. One study showed that people who used an early version of MINDFIRL were just as accurate as people who saw 100% of the identifiers. However, the people who used MINDFIRL saw 93% fewer identifiers. This means that people using MINDFIRL were just as good at patient-matching as people who saw everyone’s identifiers even though people using MINDFIRL saw far fewer identifiers. See below for an example.~~only saw 7% of the identifiers.~~

Please tell us what you think about the following statements:

	Strongly agree	Somewhat agree	Neither agree nor disagree	Disagree	Strongly disagree
The question is easy to understand.	○	○	○	○	○
	○	○	○	○	○

	Strongly agree	Somewhat agree	Neither agree nor disagree	Disagree	Strongly disagree
An answer to this question is important to me.					
The answer is easy to understand.	○	○	○	○	○
The answer contains useful information.	○	○	○	○	○

Do you have any thoughts or comments relating to the updated response above?
(If you have no comments, please type in NA)

[NEW] 2.2.12 How can MINDFIRL help patient-matching while hiding identifiers?

[NEW] 2.2.12 How can MINDFIRL help patient-matching while hiding identifiers?

In the example below, a researcher is trying to determine if the patient in Database 1 is the same as the patient in Database 2. The records are highly similar (look at the number of *), but there are some differences.

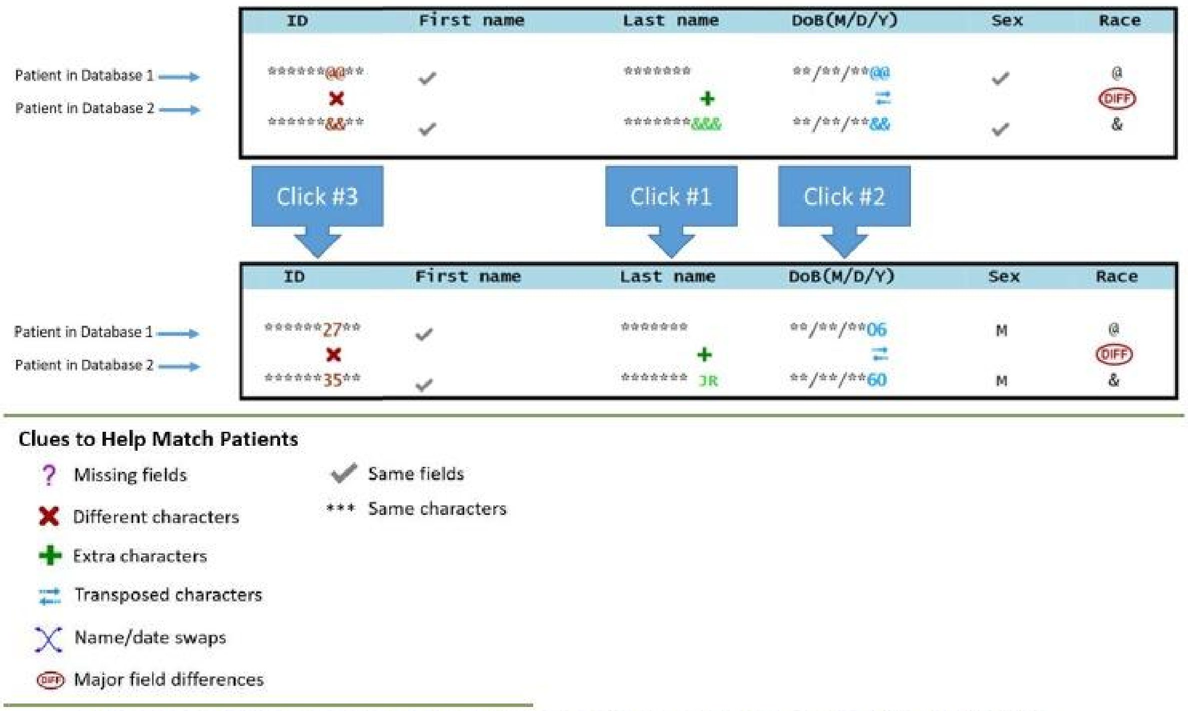

The researcher is interested in the differences in the last name so she clicks on it. With one click, MINDFIRL shows the researcher only what is different between the two records. In this case, it is possible that these are two different people, perhaps a father (Sr.) and a son (Jr.). Of course the researcher knows that it is possible that they are the same person, but the "Jr" was never recorded in Database 1, so the researcher clicks on the date of birth (Click 2). The date of birth is similar in Database 1 and Database 2, we can see that the age difference is reasonable for a father and son, but it is still possible that a typo switched the "6" and "0" in one of the databases. The researcher needs more information, so she click on the ID Number. MINDFIRL only reveals the two numbers that are different. The researcher sees that these numbers are unlikely to be a typo and is now confident that these two records represent two different people. Looking at the bottom image, you can see how MINDFIRL helps researchers link records while hiding the bulk of information from view.

Please tell us what you think about the following statements:

	Strongly agree	Somewhat agree	Neither agree nor disagree	Disagree	Strongly disagree
The question is easy to understand.	O	O	O	O	O
	O	O	O	O	O

	Strongly agree	Somewhat agree	Neither agree nor disagree	Disagree	Strongly disagree
An answer to this question is important to me.					
The answer is easy to understand.	○	○	○	○	○
The answer contains useful information.	○	○	○	○	○

Do you have any thoughts or comments relating to the **New question and answer** above?
(If you have no comments, please type in NA)

****2.3.14 What are you doing to make sure that my data is being used responsibly**

Summary of Responses

Participant suggestions included wording on what ‘data being used responsibly,’ and ‘legal guidelines for breach notification’ mean, as patients may not be familiar with these concepts. Additionally, one participant suggested that the FAQ include information on what institutional review and an IRB are. Specifically, suggestion questions for the FAQ included, “What is the IRB?’ and ‘’Why is the IRB important?’ Several participants cautioned that terms need to be explicitly defined for patients as it cannot be assumed that they are familiar with terms that researchers may be familiar with.

Based on this feedback, we have edited the response to 2.3.14 as seen here:

We note that the links in the image below will not work. So we provide the links here.

* For more on IRBs [click here]

2.3.14 What are you doing to make sure that my data is being used responsibly?

This research is ~~was~~ supervised and reviewed by the institutional review board (IRB) at [Researchers will fill in their IRB information]. The IRB is an organization that oversees research to make sure ~~it~~ the research is legal~~ly~~ and ethical~~ly permissible~~. Our IRB is responsible to protect the rights, welfare and well-being of the individuals in our research. The IRB monitors this research to make sure that we are sticking to the approved research plan. For more on IRBs click here.

We are also using the MINDFIRL software for record linkage to limit access to information that can identify you. This is part of our commitment to conducting responsible research. Furthermore, … [This information will vary depending on the specific research project, protocol, and institutional policies. MINDFIRL allows researchers to customize settings for transparency and accountability. Researchers will describe the specific safeguards that are in place to ensure responsible data use, including policies, MINDFIRL settings, and required trainings.]

Additionally, we are using secure computers and encryption to ensure that only approved researchers can access this research data safely.

Please tell us what you think about the following statements:

	Strongly agree	Somewhat agree	Neither agree nor disagree	Disagree	Strongly disagree
The question is easy to understand.	○	○	○	○	○
An answer to this question is important to me.	○	○	○	○	○
The answer is easy to understand.	○	○	○	○	○
The answer contains useful information.	○	○	○	○	○

Do you have any thoughts or comments relating to the updated response above?
(If you have no comments, please type in NA)

***2.5.18 What difference is my data going to make?**

Summary of Responses

Four (4) participants disagreed or strongly disagreed that the provided template response was easy to understand, while 3 participants indicated that they disagreed or strongly disagreed that the answer contains useful information. One participant stated that the answer to the question was not provided in the template response.

Based on this feedback, we have edited the response to 2.5.18 as seen here:

2.5.18 What difference is my data going to make?

In short, your data will help make any discoveries from this research more helpful to people like you. In research, we use information about a group of people, called a "sample," to understand things about a larger group or "population." If the sample is too different from the larger population then we cannot learn very much from the research. If people like you are not included in the research, then what we learn will not be useful to you or others like you. For example, if young adults are excluded from all studies about drug safety, it will be difficult to ever know if any drugs are safe to use on young adults. In other words, without your data it will be harder for us to understand how this research relates to people like you.

Please tell us what you think about the following statements:

	Strongly agree	Somewhat agree	Neither agree nor disagree	Disagree	Strongly disagree
The question is easy to understand.	○	○	○	○	○
An answer to this question is important to me.	○	○	○	○	○
The answer is easy to understand.	○	○	○	○	○
The answer contains useful information.	○	○	○	○	○

Do you have any thoughts or comments relating to the updated response above?
(If you have no comments, please type in NA)

SECTION 3: Order of the FAQ

SECTION 3: Order of the FAQ

In the first survey, we asked you about whether you believed the FAQ sections were in the right order. Below are the sections in the same order as Round 1:

Section 2.1: Questions about the data and identifiers
Section 2.2: Questions about MINDFIRL and the patient matching process

Section 2.3: Questions about where and how my matched data will be stored and protected
Section 2.4: Questions about the researchers
Section 2.5: Questions about the impact my data will have
Section 2.6: Questions about what happens to my data once the study is completed

A number of participants suggested moving Sections 2.4 and 2.5 earlier in the document. Some of these participants suggested that Sections 2.4 and 2.5 provide important context for the other questions. Please let us know where you think these sections should be moved to. You may review the questions in each section in the revised FAQ.

Q3.1 Where do you think the Section 2.4 questions should go?:

First

Second

Third

Fourth (do not move)

Fifth

Last

Q3.2 Where do you think the Section 2.5 questions should go?:

First

Second

Third

Fourth

Fifth (do not move)

Last

Q3.3 Please review the entire revised FAQ and provide any comments or suggestions below.
Please reference specific sections of the FAQ using the line numbers to the left of the questions and answers. For example, “I would suggest that you simplify the language in lines 25-28.”
(If you have no comments, please type in NA)

SECTION 4: Your thoughts about risk in database-only research

SECTION 4: Your thoughts about risk in database-only research

We would like to understand how you view the risks of database studies. In database studies, researchers use information that already exists. The data is usually collected for some other purpose (e.g., health care), but reused for research. Because of this, the researchers do not have direct contact with the individuals in the database.
For the purpose of these questions, you can assume the following:

- **Database research is usually approved and monitored by an Institutional Review Board (IRB), like all other research involving human participants.**
- An IRB is an organization that oversees research to make sure the research is legal and ethical. The IRB is responsible to protect the rights,

welfare and well-being of the individuals in research. For more on IRBs click here.

- **"Minimal risk" means the types of risk experienced in ordinary life.**

For each sentence below, please consider how much you agree with the given statement about database studies or risk level based on your interpretation of the rule above.

	Strongly agree	Agree	Neither agree nor disagree	Dis
Q4.I<br>The nature of the risk in the **database study** is not substantively different than risks experienced in ordinary life.	O	O	O	
Q4.2<br>Risk experienced in ordinary life in the modern digital society includes risk of privacy violation and loss of confidentiality due to linked data because data is everywhere and different dataset are linked for various purposes.	O	O	O	
Q4.3<br>As such, a **database study** involving linking data using PII is a minimal risk study as it poses no greater risks than those ordinarily encountered in everyday life.	O	O	O	
Q4.4<br>Despite the use of MINDFIRL software, which aims to reduce unnecessary disclosures for purposes of record linkage, all database study have a risk of unexpected disclosures due to a potential breach of the computer system hosting the data.	O	O	O	
Q4.5<br>The risk of unexpected disclosures due to a potential breach of the computer system with the data is not more than those encountered in ordinary life, considering the numerous breaches of computer systems in the news.	O	O	O	
Q4.6<br>The use of the MINDFIRL software will further reduce risk to the minimum necessary to conduct reliable record linkage.	O	O	O	

	Strongly agree	Agree	Neither agree nor disagree	Dis
Q4.7<br>Overall, do you believe that the risks of a **database study** (with access to identifiable information) are greater than minimal risk for "regular health data" (i.e., excluding highly confidential data such HIV, mental health, substance abuse).	○	○	○	
Q4.8<br>Overall, do you believe that the risks of a **database study** (with access to identifiable information) are greater than minimal risk for sensitive data such as HIV, mental health, substance abuse.	○	○	○	

Q4.9 Please provide your thoughts and feedback about any of the above statements in the space below:

(If you have no comments, please type in NA)

Q4.10 Do you think there are database-only studies (i.e., there is no contact with participants) that may constitute greater than minimal risk (i.e., greater than the types of risk experienced in ordinary life)?
(If you have no comments, please type in NA)

Q4.11 If Yes, please specify what might constitute greater than minimal risk in a database only study (i.e., greater than the types of risk experienced in ordinary life).
(If you have no comments, please type in NA)

Closing

If you are curious about the breakdown of responses from all questions in Round 1, you can access a document containing this information here:

We thank you for your participation in our Delphi study.

Please select your preferred method of payment:

Amazon gift card

Target gift card

Thank you for your help in shaping the content of the MINDFIRL FAQ. We plan to send you the link to the final round of this study (Round 3) in early June.

Powered by Qualtrics
