# Supplementary material for: Communicating With Patients About Software for Enhancing Privacy in Secondary Database Research Involving Record Linkage: Delphi Study
Source: J Med Internet Res. 2020 Dec 15;22(12):e20783. doi: 10.2196/20783 (PMC7772068; doi:10.2196/20783)
Supplement: Multimedia Appendix 4 [file jmir_v22i12e20783_app4.pdf]

This is a Multimedia Appendix to a full manuscript published in the J Med Internet Res.
For full copyright and citation information see http://dx.doi.org/10.2196/jmir.20783

SUMMARY OF ROUND 2

There were 35 participants in Round 1. Based on your responses, there were a few questions and responses that were particularly problematic. In the table below, we have provided a summary of the questions and responses in which fewer than 80% of the participants strongly agreed or agreed with ease of the questions and answers provided or the importance and understandability of the template responses.

**Particularly Problematic**	
**Question and Template Response**	**Summary of Responses**
**Does MINDFIRL reduce the quality of matched records? [2.2.11]**<br><br>**Template Response:**<br>No. One study showed that people who used an early version of MINDFIRL were just as accurate as people who saw 100% of the identifiers. However, the people who used MINDFIRL saw 93% fewer identifiers. This means that people using MINDFIRL were just as good at patient-matching as people who saw everyone's identifiers even though people using MINDFIRL saw far fewer identifiers. See below for an example.	Eleven participants (31%) did not strongly agree or agree with questions concerning the ease of the question presented, the importance of the answer, the understandability of the template response or the usefulness of the information presented in the template response. Generally, these participants provided feedback indicating that the template response was clunky and not appropriate for a lay audience.<br><br>On the other hand, several participants indicated that the revised template language was must better than the response provided in Round 1, and that the question and response were easy to understand.
**What difference is my data going to make? [2.5.18]**<br><br>**Template Response:**<br>In research, we use information about a group of people, called a "sample," to understand things about a larger group or "population." If the sample is too different from the larger population then we cannot learn very much from the research. If people like you are not included in the research, then what we learn will not be useful to you or others like you. For example, if young adults are excluded from all studies about drug safety, it will be difficult to ever know if any drugs are safe to use on young adults.	Ten participants (29%) did not strongly agree or agree with questions concerning the ease of the question presented, the importance of the answer, the understandability of the template response or the usefulness of the information presented in the template response.<br><br>Some participants suggested that the phrasing of the question did not align with the template response. Suggestions were made for re-phrasing (e.g. "How will my data be useful for this research?" One participant suggested removal of the phrase "people like you." Another suggested that the template response was too 'textbook.'
**What pieces of information about me will the researchers see? [2.1.3]**<br><br>**Template Response:** [Note: we will revise identifier/non-identifier terminology based on survey feedback in Round 2.]<br>We need different information for different steps of the research process. We only need identifiers to do patient matching. Additionally, we only need non-identifiers when we are using your health related data to learn more about science or medicine. We will use a software program called MINDFIRL (MINimum Necessary Disclosure For	Twelve participants (34%) did not strongly agree or agree with the questions concerning the ease of the question presented, the importance of the answer, the understandability of the template response, or the usefulness of the information presented in the template response. Some participants indicated that there was too much detail in the response for patients to digest (i.e., the FAQ should be shortened). Others suggested that more detail is needed. For example, one participant stated, "This is important information for people to have if they want it. Initially I thought that this might be too much information, but then I reread it and I think that the changes make it easier to understand. Most people

Interactive Record Linkage) to keep identifiers separate from non-identifiers and ensure that no one can access identifiers and non-identifiers together at the same time. In many cases, patient matching is done all with a linkage software, like MINDFIRL, and a person may never see your identifiers because the computer is automatically matching patients without human effort. For others records where the computer is not sure, researchers are asked to determine the match manually.<br>a. Who will be able to see the identifiers?<br>The researchers that will be doing the record matching will have access to identifiers. Information such as your name, date of birth, marital status, and gender help distinguish you from other people. Our researchers need to access identifiers to match patient records.<br><br>We are using the MINDFIRL software to protect identifiers and prevent unnecessary privacy loss during this process. First, MINDFIRL separates identifiers from the non-identifiers. This means that, no one can access the identifiers AND the health-related data at the same time. Second, MINDFIRL tells researchers when two records have the same identifiers without showing details. In these cases, our researchers might not need to see specific identifiers to make a match. MINDFIRL also tells researcher when records are highly similar without showing details. MINDFIRL only shows identifiers on an 'as needed' basis. For example, a researcher might want to see some details to know if a difference is important (e.g, to tell twins apart). This means that MINDFIRL can help catch common matching problems, such as nicknames (e.g., Pam v. Pamela) or typos (e.g., John v. Jonh), without showing the rest of your identifiers.<br><br>b. Who will be able to see the Non-identifiers or health-related study data?<br>Non-identifiers are everything else in the data. Non-identifiers could include information such as diagnosis, medications, or blood pressure. Our researchers will only use the non-identifiers for the main research after the matching is done. In some cases, the same researchers who match the records will	who are going to take the time to read through this don't mind if it is too much information. They mind if it is not enough to understand. I like the changes proposed," while another stated, "Too long, too much detail."

use the non-identifiers for the main research. However, MINDFIRL separates identifiers from the non-identifiers. This means that, no one can access the identifiers AND the health-related data at the same time. We will code your non-identifiers to protect your identity. This allows us to use your information to make scientific or medical discoveries without knowing which information belongs to you.	
**What will you do if you discover that an unauthorized person has accessed my data or my data has been otherwise misused? [2.3.15]**<br><br>Q1.5 including links to the legal requirements is important for this answer.<br>**Response:**<br>Respondents that answered "Neutral" = 5<br>Respondents that answered "Disagree or Strongly Disagree" =1<br><br>Q1.6 including links to any organizational rules is important for this answer.<br>**Response:**<br>Respondents that answered "Neutral" = 9<br>Respondents that answered "Disagree or Strongly Disagree" =2<br><br>Q1.7 The researchers should summarize any legal breach notification requirements.<br>**Response:**<br>Respondents that answered "Neutral" = 4<br>Respondents that answered "Disagree or Strongly Disagree" =3<br><br>Q1.8 The researchers should summarize any organizational breach notification requirements.<br>**Response:**<br>Respondents that answered "Neutral" = 8<br>Respondents that answered "Disagree or Strongly Disagree" =2<br><br>Q1.9 The researchers should summarize any breach notification processes required by the Institutional Review Board (IRB).<br>**Response:**<br>Respondents that answered "Neutral" = 4<br>Respondents that answered "Disagree or Strongly Disagree" =2	Eleven participants (31%) disagreed, strongly disagreed or were neutral about whether links to organizational rules is important for inclusion in the FAQ (Q1.6).<br><br>Ten participants (29%) disagreed, strongly disagreed or were neutral about whether researchers should summarize any organizational breach notification requirements in the FAQ (Q1.8).<br><br>For all other sub-questions (Q1.5. Q1.7, and Q1.9), 80% or more of the participants either agreed or strongly agreed with inclusion of summaries of and links to legal requirements and breach notification processes of the researchers' Institutional Review Board(s).

You may be interested in a breakdown of the responses to each question and template response in the FAQ. The graphs below provide this breakdown.

Q1.1 Please indicate the term that you prefer the most for "identifiers":

a) **Identifiers. N= 3**
b) **Identifying information. N =15**
c) **Identifiable information. N= 4**
d) **Information that can be used to identify individuals. N =13**

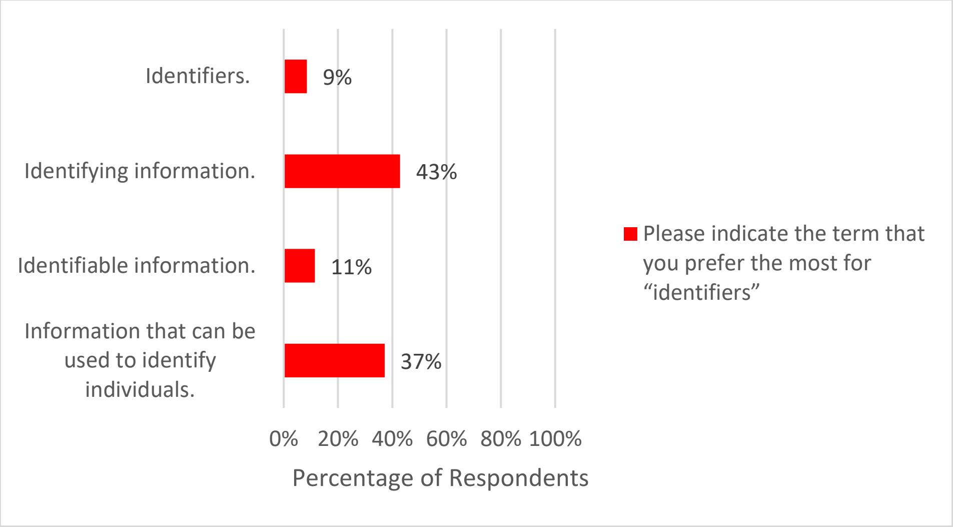

Q1.3 Please indicate the term that you prefer the most for "non-identifiers":

a) **Non-identifiers. N= 2**
b) **Non-Identifying information. N= 22**
c) **Non identifiable information. N= 4**
d) **Health-related study data. N= 7**

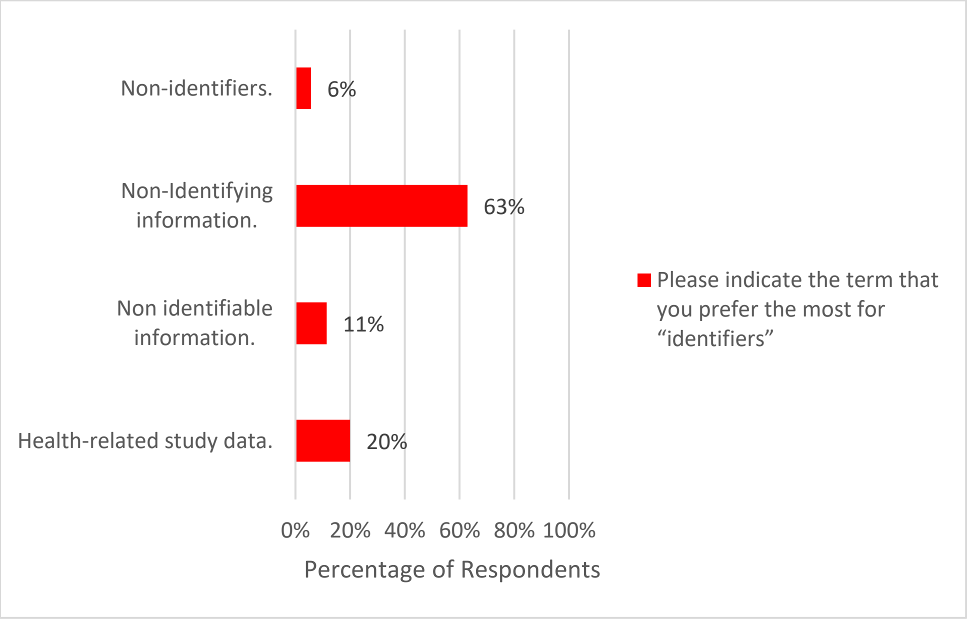

A number of participants wanted more information relating to the "legal guidelines" mentioned in the following question and answer:

2.3.15 What will you do if you discover that an unauthorized person has accessed my data or my data has been otherwise misused?

While we take great measures to safeguard your data, if a data breach were to occur, we would follow legal guidelines for breach notification.

This is a difficult question to answer in general language because there are many different privacy laws that could apply to different research projects. We would like your help in identifying a better answer for a) patients reading this FAQ and b) researchers who want to adapt and use this FAQ for their projects.

Please respond to the following statements:

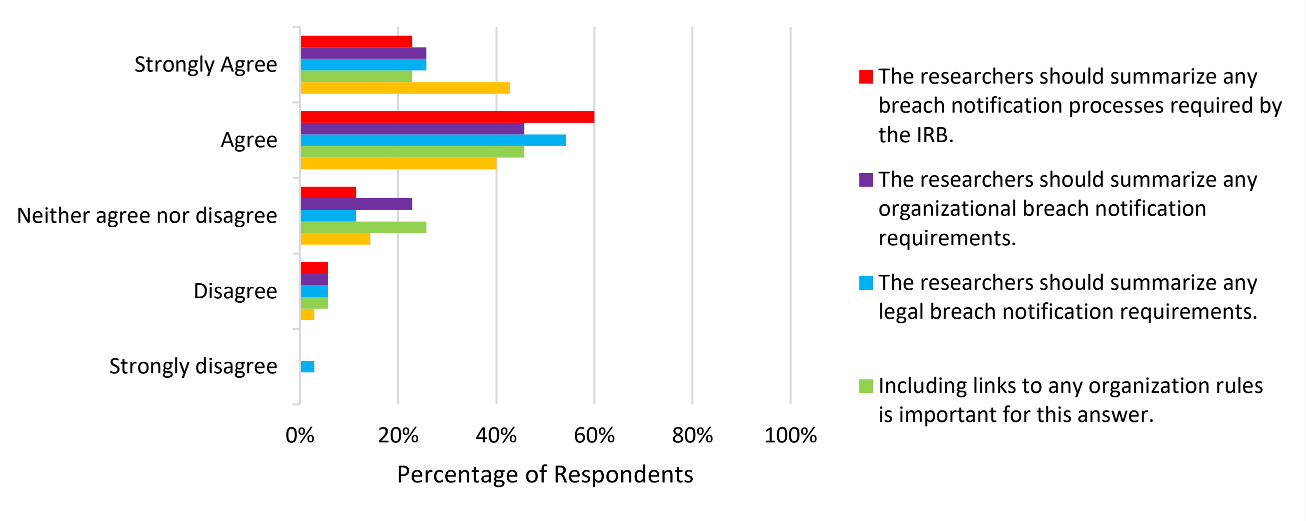

Error! Reference source not found.

[Note: we will revise identifier/non-identifier terminology based on survey feedback in Round 2.]

We need different information for different steps of the research process. We only need identifiers to do patient matching. Additionally, we only need non-identifiers when we are using your health related data to learn more about science or medicine. We will use a software program called MINDFIRL (MINimum Necessary Disclosure For Interactive Record Linkage) to keep identifiers separate from non-identifiers and ensure that no one can access identifiers and non-identifiers together at the same time. In many cases, patient matching is done all with a linkage software, like MINDFIRL, and a person may never see your identifiers because the computer is automatically matching patients without human effort. For others records where the computer is not sure, researchers are asked to determine the match manually.

a. Who will be able to see the identifiers?

The researchers that will be doing the record matching will have access to identifiers. Information such as your name, date of birth, marital status, and gender help distinguish you from other people. Our researchers need to access identifiers to match patient records.

We are using the MINDFIRL software to protect identifiers and prevent unnecessary privacy loss during this process. First, MINDFIRL separates identifiers from the non-identifiers. This means that no one can access the identifiers AND the health-related data at the same time. Second, MINDFIRL tells researchers when two records have the same identifiers without showing details. In these cases, our researchers might not need to see specific identifiers to make a match. MINDFIRL also tells researcher when records are highly similar without showing details. MINDFIRL only shows identifiers on an 'as needed' basis. For example, a researcher might want to see some details to know if a difference is important (e.g, to tell twins apart). This means that MINDFIRL can help catch common matching problems, such as nicknames (e.g., Pam v. Pamela) or typos (e.g., John v. Jonh), without showing the rest of your identifiers.

b. Who will be able to see the Non-identifiers or health-related study data?

Non-identifiers are everything else in the data. Non-identifiers could include information such as diagnosis, medications, or blood pressure. Our researchers will only use the non-identifiers for the main research after the matching is done. In some cases, the same researchers who match the records will use the non-identifiers for the main research. However, MINDFIRL separates identifiers from the non-identifiers. This means that no one can access the identifiers AND the health-related data at the same time. We will code your non-identifiers to protect your identity. This allows us to use your information to make scientific or medical discoveries without knowing which information belongs to you.

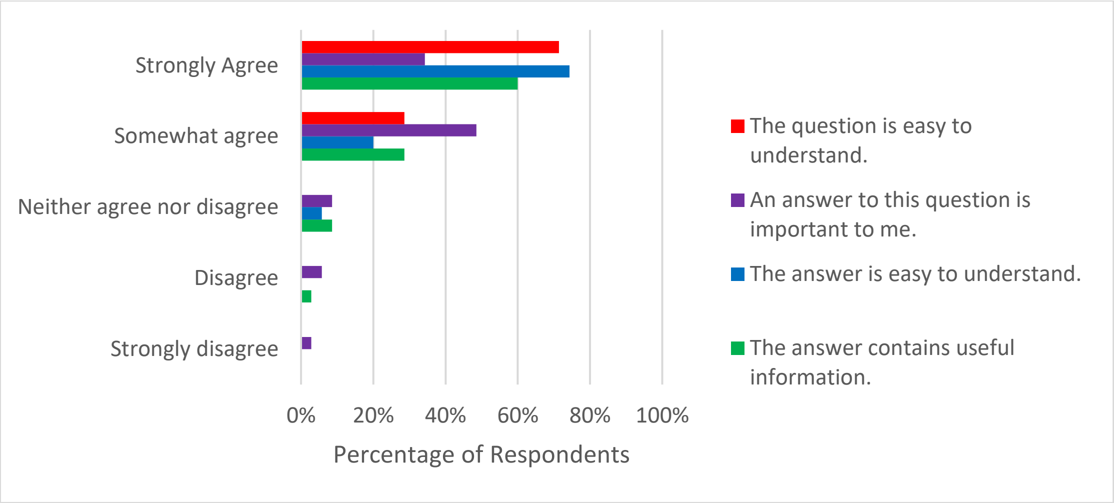

Error! Reference source not found.

Patient Matching is the process of linking records of the same real-world person from different databases. Patient matching helps researchers answer difficult questions. For example, is disease treatment A better than treatment B for keeping patients healthy? To do this, we might want to count the number of emergency room visits Jane Doe made this year across several hospitals. This requires linking records from all the hospitals she visited.

This is hard because a universal identification number does not exist to easily link records in different systems. Instead, we have to use the personally identifiable information (PII), like

social security numbers (SSNs), first & last names, birthdates, race, and gender to decide which records belong to the same person.
Still, patient matching is hard. Identifiers are not unique. Different people may share the same name. Names are inconsistent (e.g. nicknames). Data is sometimes missing (e.g. SSNs are often missing). Names change over time (e.g., changing a last name at marriage). Data can have errors (e.g. typos). Below is an example of what patient-matching might look like:

	ID	First name	Last name	DoB(M/D/Y)	Sex	Race
Patient in Database 1 →	8000002767	JUDE	WILLIAM	09/09/1906	M	W
Patient in Database 2 →	8000003567	JUDE	WILLIAM JR	09/09/1960	M	B

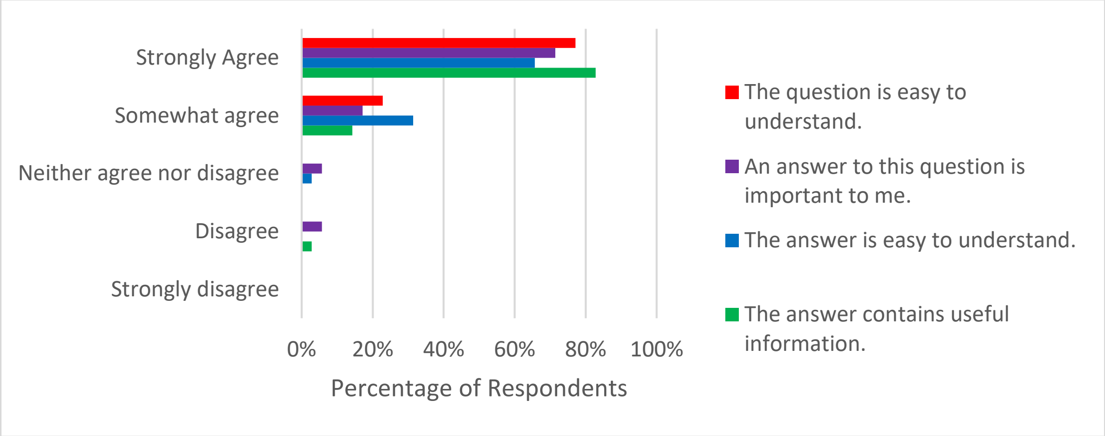

Error! Reference source not found.

MINDFIRL (Minimum Necessary Disclosure for Interactive Record Linkage) is a software that we use to help protect privacy in the patient matching process of our research. It protects privacy in a few ways. First, it separates the identifiers from the health-related study information. That way, the researchers can view identifiers to help them accurately match records without seeing sensitive information.

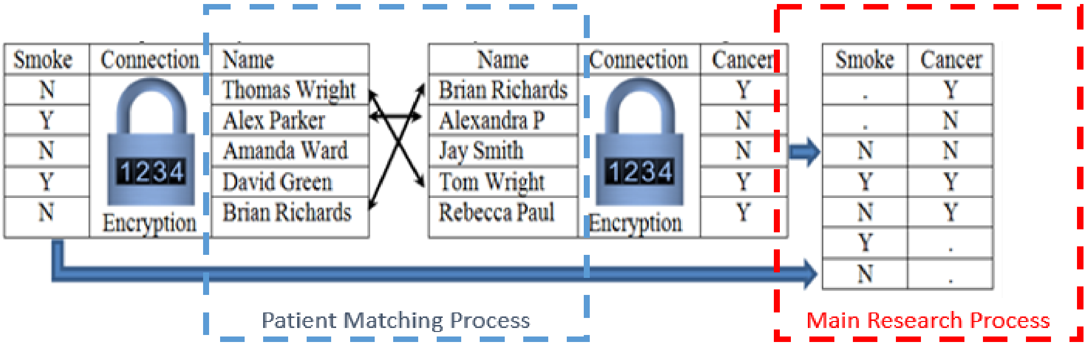

This image shows how MINDFIRL helps the patient-matching process while limiting access to sensitive information. The identifiers are separated from the rest of the information during the patient-matching process. In this way, if a researcher sees someone's identifying information, the researcher cannot see any other sensitive information at the same time.

Second, MINDFIRL hides identifiers, while giving our researchers clues about how similar or different the same identifier is in two records. That way, MINDFIRL allows our researcher to match records without seeing the specific identifiers. See below.

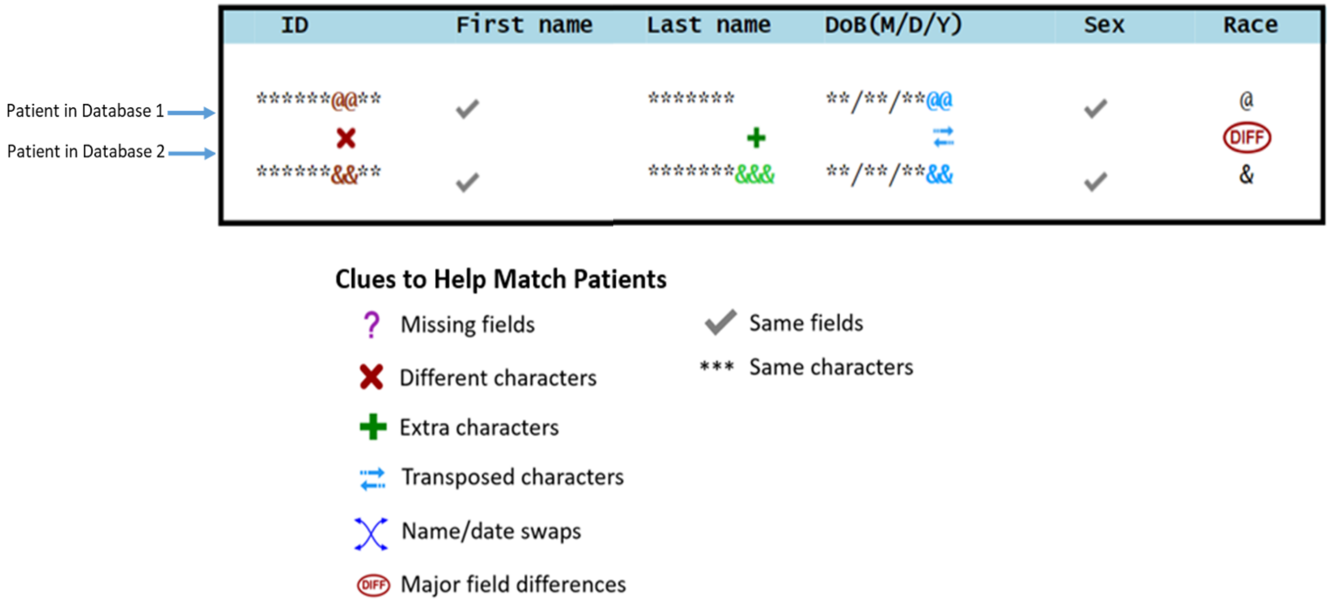

Third, MINDFIRL allows researchers to selectively reveal information to help accurately match patients. This way, a research does not have to see every identifier in order to make a correct match. You can see how this works in this clickable demo (You must use chrome on your PC for this demo to work well. The link will not work on a phone.): http://mindfil4.herokuapp.com/tutorial/clickable/demo.

Finally, MINDFIRL tracks and records the information that researchers see. In this way, the research supervisors can ensure that the researchers who are matching records are not abusing their position by revealing too much.

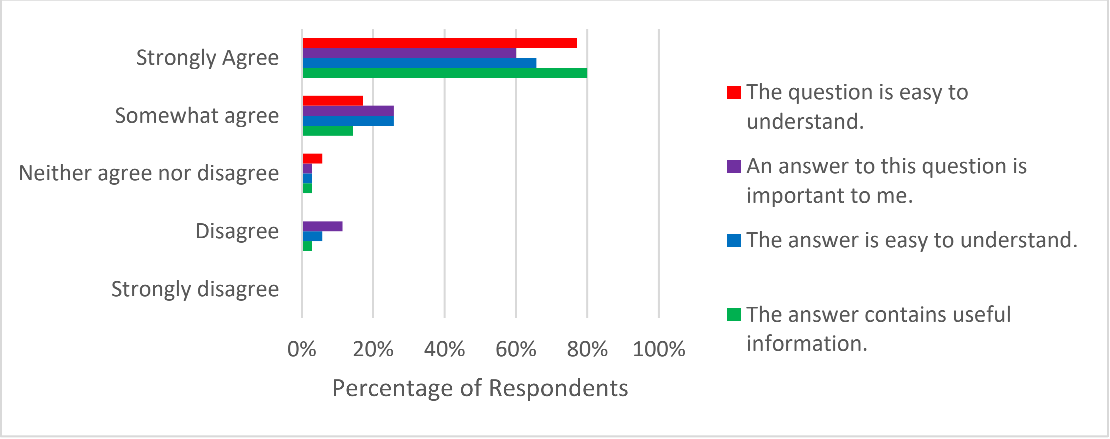

Error! Reference source not found.

It is unlikely, but possible, that you might be identified in the linked data. We remove all identifiers in the linked data, so if someone wanted to identify you in the linked data they would have to use some other information to tell you apart from everyone in the linked data (and the rest of the world). We use encryption and secure computer systems to protect this data to reduce the risk that someone can identify you in the data.

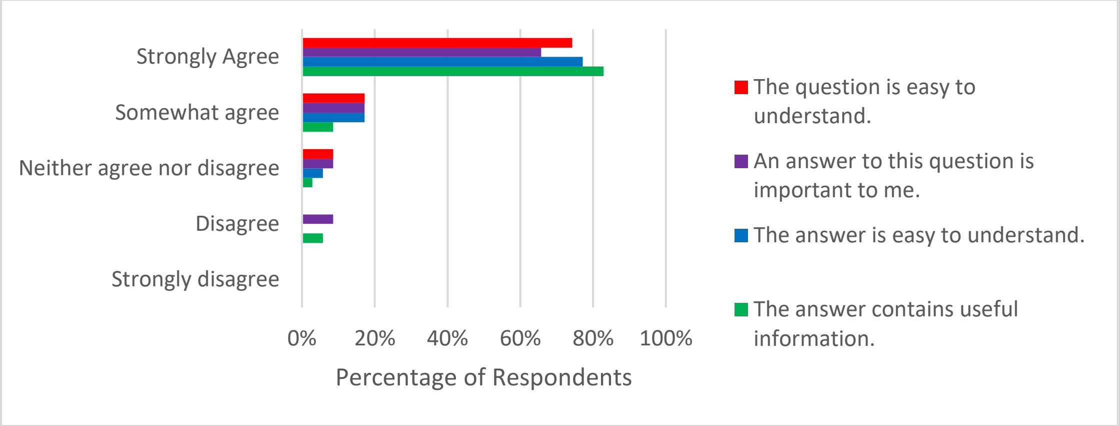

Error! Reference source not found.

Yes, MINDFIRL is designed to improve privacy in database studies. As a result, we expect that MINDFIRL will lower the risk of individual studies. MINDFIRL includes tools to promote transparency and monitor researcher activity to limit risk. For example, MINDFIRL tracks what identifiers are viewed and who viewed them. This is similar to a store that has a surveillance camera to make sure that the cashier does not take money from the cash register. This tracking information is used to discourage the misuse of your information. It also allows for setting hard limits on how much data is used. See figure below.

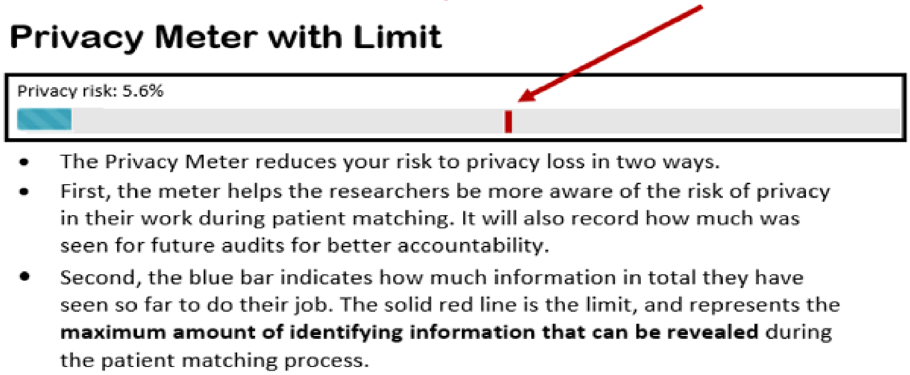

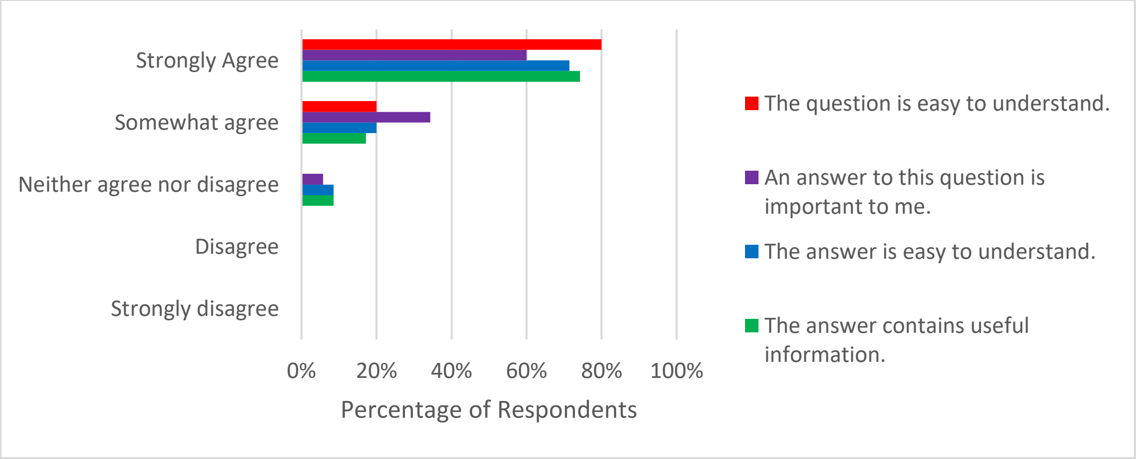

Error! Reference source not found.

No. One study showed that people who used an early version of MINDFIRL were just as accurate as people who saw 100% of the identifiers. However, the people who used MINDFIRL saw 93% fewer identifiers. This means that people using MINDFIRL were just as good at patient-matching as people who saw everyone's identifiers even though people using MINDFIRL saw far fewer identifiers.

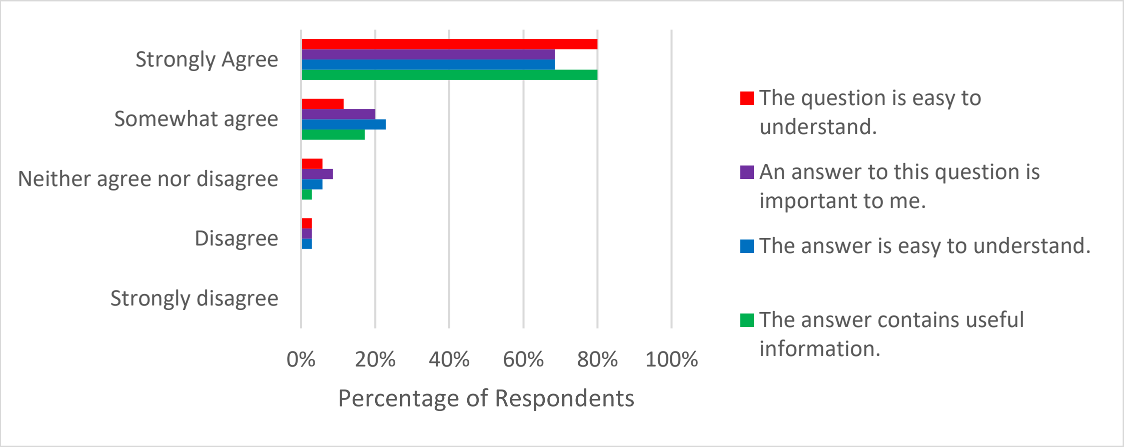

Error! Reference source not found.

In the example below, a researcher is trying to determine if the patient in Database 1 is the same as the patient in Database 2. The records are highly similar (look at the number of *), but there are some differences.

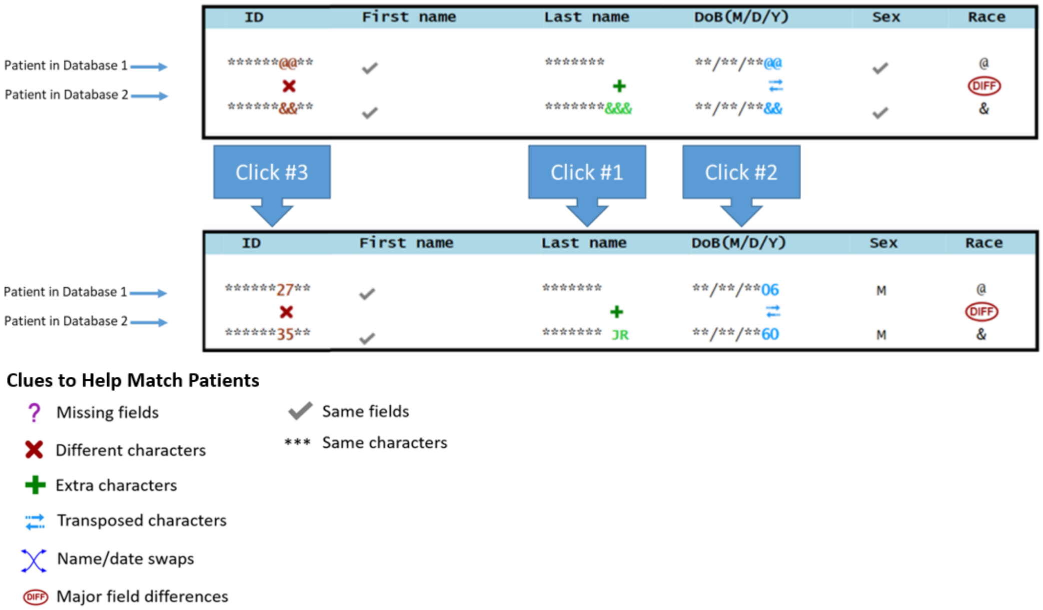

The researcher is interested in the differences in the last name so she clicks on it. With one click, MINDFIRL shows the researcher only what is different between the two records. In this case, it is possible that these are two different people, perhaps a father (Sr.) and a son (Jr.). Of course the researcher knows that it is possible that they are the same person, but the "Jr" was never recorded in Database 1, so the researcher clicks on the date of birth (Click 2). The date of birth is similar in Database 1 and Database 2, we can see that the age difference is reasonable for a father and son, but it is still

possible that a typo switched the “6” and “0” in one of the databases. The researcher needs more information, so she click on the ID Number. MINDFIRL only reveals the two numbers that are different. The researcher sees that these numbers are unlikely to be a typo and is now confident that these two records represent two different people. Looking at the bottom image, you can see how MINDFIRL helps researchers link records while hiding the bulk of information from view.

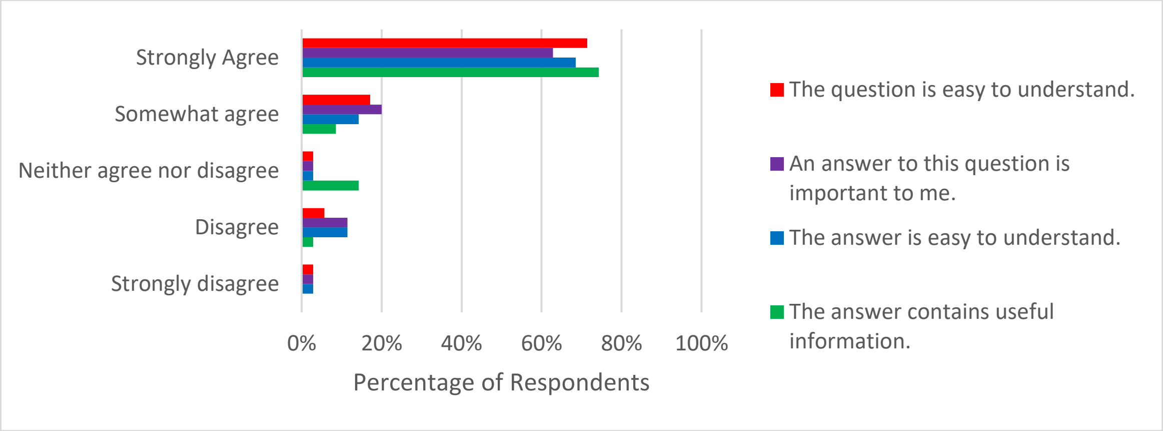

Error! Reference source not found.

This research is supervised and reviewed by the institutional review board (IRB) at [Researchers will fill in their IRB information]. The IRB is an organization that oversees research to make sure the research is legal and ethical. Our IRB is responsible to protect the rights, welfare and well-being of the individuals in our research. The IRB monitors this research to make sure that we are sticking to the approved research plan. For more on IRBs click here.

We are also using the MINDFIRL software for record linkage to limit access to information that can identify you. This is part of our commitment to conducting responsible research. Furthermore, … [This information will vary depending on the specific research project, protocol, and institutional policies. MINDFIRL allows researchers to customize settings for transparency and accountability. Researchers will describe the specific safeguards that are in place to ensure responsible data use, including policies, MINDFIRL settings, and required trainings.]

Additionally, we are using secure computers and encryption to ensure that only approved researchers can access this research data safely.

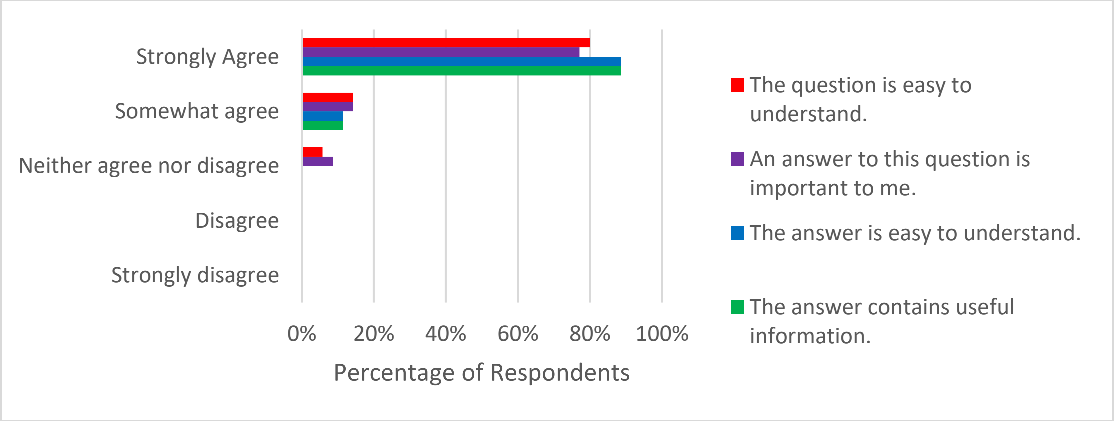

Error! Reference source not found.

In short, your data will help make any discoveries from this research more helpful to people like you. In research, we use information about a group of people, called a "sample," to understand things about a larger group or "population." If the sample is too different from the larger population then we cannot learn very much from the research. If people like you are not included in the research, then what we learn will not be useful to you or others like you. For example, if young adults are excluded from all studies about drug safety, it will be difficult to ever know if any drugs are safe to use on young adults. In other words, without your data it will be harder for us to understand how this research relates to people like you.

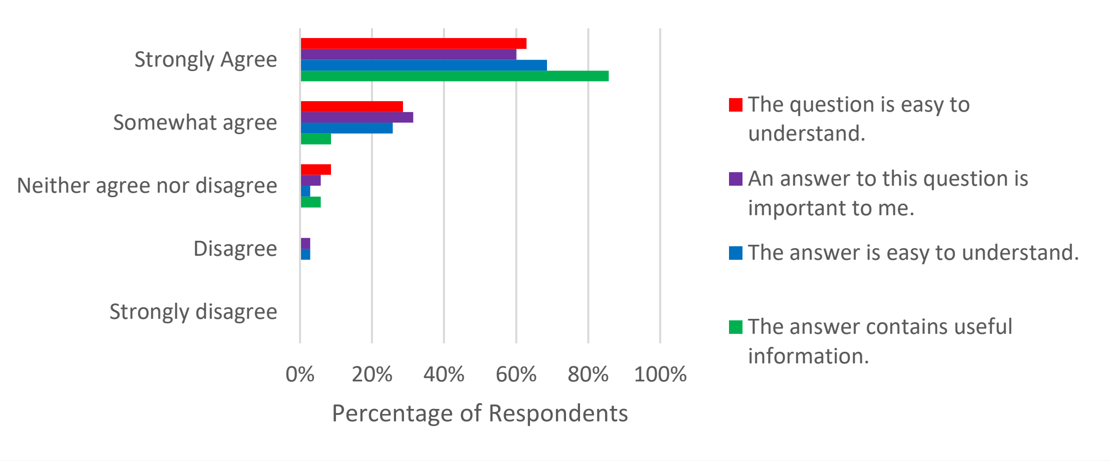

SECTION 3: Order of the FAQ
In the first survey, we asked you about whether you believed the FAQ sections were in the right order. Below are the sections in the same order as Round 1:

Section 2.1: Questions about the data and identifiers
Section 2.2: Questions about MINDFIRL and the patient matching process
Section 2.3: Questions about where and how my matched data will be stored and protected
Section 2.4: Questions about the researchers
Section 2.5: Questions about the impact my data will have
Section 2.6: Questions about what happens to my data once the study is completed

Q3.1 Where do you think the Section 2.4 questions should go?

First: 8
Second: 9
Third: 0
Fourth (Do not move): 12
Fifth: 2
Last: 4

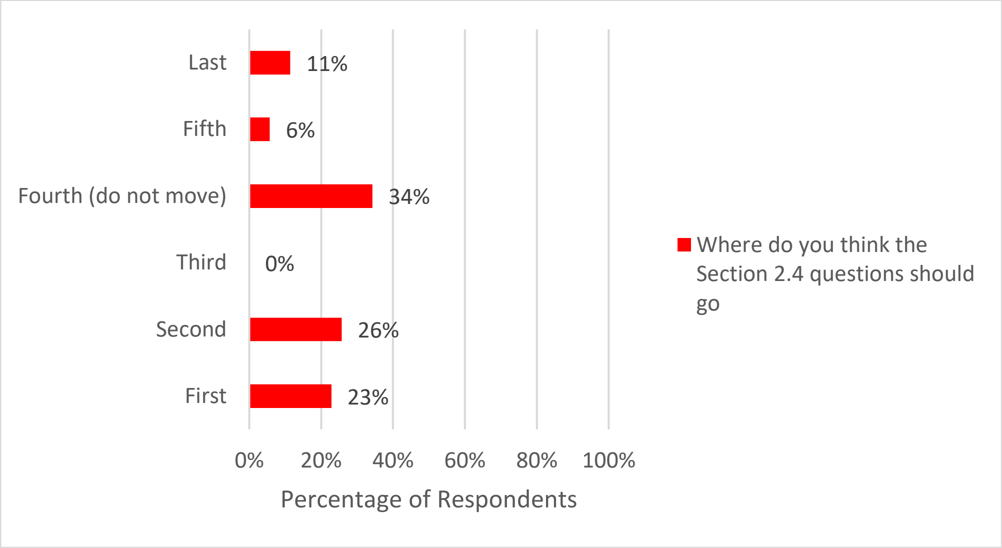

Q3.2 Where do you think the Section 2.5 questions should go:
First: 6
Second: 7
Third: 5
Fourth: 1
Fifth (Do not move): 13
Last: 3

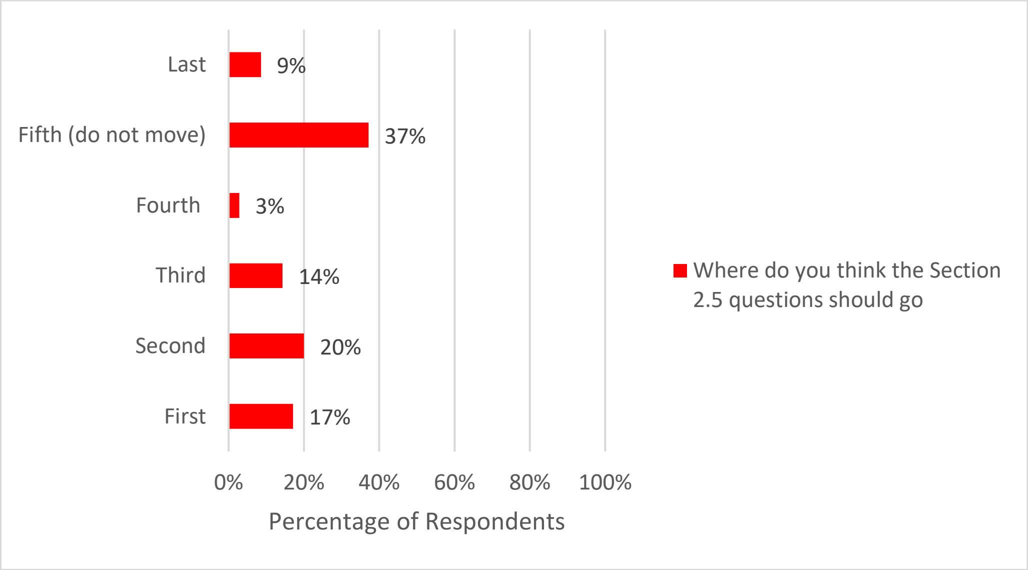

SECTION 4: Your thoughts about risk in database-only research

We will provide a summary of the questions about risk in database-only research following Round 3.
