# Supplementary material for: Communicating With Patients About Software for Enhancing Privacy in Secondary Database Research Involving Record Linkage: Delphi Study
Source: J Med Internet Res. 2020 Dec 15;22(12):e20783. doi: 10.2196/20783 (PMC7772068; doi:10.2196/20783)
Supplement: Multimedia Appendix 5 [file jmir_v22i12e20783_app5.pdf]

This is a Multimedia Appendix to a full manuscript published in the J Med Internet Res.
For full copyright and citation information see http://dx.doi.org/10.2196/jmir.20783

Introduction

Dear participant,

We thank you once again for your effort and feedback during the previous two rounds of our Delphi study. We are now moving to the 3rd and final round of the study.

The following pages contain follow up questions based on your Round 2 feedback, questions related to FAQ revisions, and a new MINDFIRL demonstration video, which we re-designed and adjusted based on your comments. In addition, we would like to gather your opinion on the FAQ document as an interactive website.

You will also find a link with a document which summarizes your votes, feedback, and comments in Round 2. To download the summary document, please click here: **Link for summary document**

We look forward to your responses and we once again thank you for your time.

Part 1

Part 1: Revisions to Selected FAQs.

In both Round 1 and Round 2, we received feedback supporting more detail and feedback requesting that we shorten the FAQs. A number of participants thought that the below FAQ is too long.

Please tell us if you think that the FAQ would be better without paragraphs (a) and (b).

What pieces of information about me will the researchers see?

We need different information for different steps of the research process. We need identifying information only to do patient matching (see section 2.1). Additionally, we need non-identifying information only when we are using your health related data to learn more about science or medicine. We will use a software program called MINDFIRL (Minimum Necessary Disclosure For Interactive Record Linkage) to keep identifying information separate from non-identifying information and **ensure that no one can access identifying information and non-identifying information together at the same time.** (for MINDFIRL, see sections 2.2 & 2.3)

In many cases, patient matching is done entirely with a linkage software, like MINDFIRL, and **a human may never see your identifying information** because the computer is automatically matching patients without human effort. For other records where the computer is not sure, researchers must manually make the matches.

a. Who will be able to see the identifying information?
The researchers who will be doing the patient matching will have access to identifying information. Information such as your name, date of birth, marital status, and gender help distinguish you from other people. Our researchers need to access identifying information to match patient records.

We are using the MINDFIRL software (see section 2.2) to protect identifying information and prevent unnecessary privacy loss during this process. First, MINDFIRL separates identifying information from the non-identifying information. This means that, **no one can access the identifying information AND the health-related data at the same time.**

Second, MINDFIRL tells researchers when two records have the same identifying information without showing details. In these cases, our researchers might not need to see any specific identifying information to make a match.

MINDFIRL also tells researcher when records are highly similar without showing details. MINDFIRL shows identifying information only on an 'as needed' basis. For example, a researcher might want to see some details to know if a difference is important (e.g, to tell twins apart). This means that MINDFIRL can help catch common matching problems, such as nicknames (e.g., Pam v. Pamela) or typos (e.g., John v. Jonh), without showing the rest of your identifying information. To see how MINDFIRL works with a specific example, see Section 2.3.

b. Who will be able to see the Non-identifying information or health-related study data?

The researchers who are doing the main research will be able to see the non-identifying, health-related data. The main research takes place after the patient-matching process and after all identifying information has been removed from the data using the MINDFIRL software. This means that, no one can access the identifying information AND the health-related data at the same time. We will code your non-identifying information to protect your identity. **This allows us to use your information to make scientific or medical discoveries without knowing which information belongs to you.**

Please tell us if you think that the FAQ would be better without paragraphs (a) and (b).

Yes, the FAQ is better without paragraphs (a) and (b)

No, the FAQ is better with paragraphs (a) and (b)

Do you have any other thoughts or comments about the FAQ above?
(If you have no comments, please type N/A)

Part 2

<u>Part 2: Revisions to Selected FAQ, continued.</u>

We received a number of comments relating to the FAQ “Does MINDFIRL reduce the quality of matched records?”. Some responders thought that the answer was too complicated for lay people. Other commenters provided suggestions for describing the study results. Some people thought the FAQ was better before the revisions.

Below are some questions relating to this FAQ.

Please help us choose the best way to communicate these study results to patients.

Which do you prefer?

The people who used the MINDFIRL prototype saw 93% less identifying information.

The people who used MINDFIRL prototype only saw 7% of the identifying information.

The MINDFIRL group needed 7 pieces of information for every 100 pieces available to the other group.

The people who used MINDFIRL saw over 14 times less identifying information.

None of these

Please let us know if you have any comments related to the previous question. (If you have no comments, please type in N/A)

Part 2.1

Please choose which approach to this question you prefer. Please note we will update the study details based on the results of the question above.

OPTION A: Does MINDFIRL reduce the quality of matched records?
Early evaluations of MINDFIRL show that it helps researchers make high-quality matches. People who used a prototype version of MINDFIRL were just as accurate as people who saw all of the identifying information. However, the people who used the MINDFIRL prototype saw 93% less identifying information. This means that **people using MINDFIRL were just as good at patient-matching as people who saw everyone's identifying information even though people using MINDFIRL saw far less identifying information.**

OPTION B: Does MINDFIRL reduce the quality of matched records?
No. One study showed that people who used an early version of MINDFIRL were just as accurate as people who saw 100% of the identifying information. However, the people who used MINDFIRL only saw 7% of the identifying information.

Which option do you prefer?

Option A

Option B

Do you have any other thoughts or comments about the FAQ above?
(If you have no comments, please type in N/A)

Part 3

<u>Part 3: Thoughts About Risk in Database Research</u>

In Round 2, we asked some questions about how you think about risk in database research. A number of the participants thought that many of these questions were difficult to understand. Based on this feedback, we re-worded the questions and have provided some additional background information that we hope will be helpful.

Below we provide background information on **1) "minimal risk" research, and 2) database research.** Please read and consider this information when answering the questions below.

"Minimal Risk" Research

The law that regulates research has some flexibility depending on how risky a study is. When the risks of research are high, the law requires a lengthy review process before the research can begin. If the risks are low, the law allows a quicker review process. Low-risk studies are sometimes called “minimal risk” research. Only research with “minimal risk” can go through this quick review process. The laws define “minimal risk” as the types of risk that we experience in daily life.

Here are some examples of **“Minimal Risk”** research:

- An identifiable survey or interview about a software (in daily life people regularly share opinions about products or experiences and sharing opinions about software is usually not risky)
- Data collected through observation of public behavior
- Research involving collecting simple blood tests (in daily life, patients will often get shots or injections, or routine blood work, and these tests are rarely risky or harmful)
- Research requiring healthy participants to do moderate exercise (exercise is an activity experienced in daily life)
- Research involving standard psychological assessments (these types of tests are not generally considered harmful)

Some examples of high-risk research might include:

- A survey about past criminal behavior (research participants can face jail or prison time if their answers are discovered)
- Research testing a new cancer drug on patients (the drug could have unintended harmful effects)

• Research where some or all of the participants could be vulnerable to coercion or undue pressure, such as children, prisoners, individuals with impaired decision-making, or other disadvantaged persons

It is important to note that “minimal risk” does not mean “risk-free.” Some everyday activities, like driving a car, are quite risky. As long as the risks are not greater than the types of risks experienced in daily life, the research is considered “minimal risk.”

“Minimal risk” research must still be approved by an Institutional Review Board (IRB). An IRB is an organization that reviews research plans to make sure the research is legal and ethical. The IRB is responsible for protecting the rights, welfare and wellbeing of research participants. **However, research that is minimal risk can be reviewed and approved in a more streamlined and efficient process,** and the IRB can always require specific protections to “minimal risk” research. Without this legal flexibility, low-risk research would experience significant delays and research costs would increase.

Database Research

In the questions below, we use the phrase “database research” to refer to research where:

1) The researchers use information that already exists, and

2) The information might have been collected for some other, non-research, purpose (e.g., medical records), and

3) The researchers have no contact with the individuals in the data, and

4) It would be very difficult, if not impossible, for researchers to contact the individuals in the data to get informed consent for the research.

Here are two examples of database research:

- Researchers using insurance data (e.g. data from Blue Cross Blue Shield) to understand how medical costs have changed for diabetes patients over five years.
- Researchers using hospital discharge data to understand why patients might be coming back to the hospital within 30 days (e.g. living alone, age, insurance status, and diagnosis).

In each of these examples, researchers are using information that has already been collected by insurance companies or hospitals for a main purpose that is not research (i.e., treatment or billing). The researchers did not have contact with the individuals in the data, and it would be difficult, if not impossible for the researchers to contact each person to obtain informed consent.

For each sentence below, please consider how much you agree with the given statement about database research or risk level based on your interpretation of the information above.

	Strongly agree	Agree	N[illegible] a[illegible] dis[illegible]
Q4.1 The types of risks that a person faces in database research are not meaningfully different from the other risks a person might experience in daily life.	○	○	
Q4.2 In today's technology driven world, data is everywhere, so people experience privacy and confidentiality risks daily because businesses, tech companies, and governments use and link our data for different purposes all the time.	○	○	
Q4.3 Database research that involves linking different datasets using identifiable information is "minimal risk" research because the risks are not greater than the types of risks experienced in daily life.	○	○	

	Strongly agree	Agree	N a dis
Q4.4 Even though MINDFIRL helps limit access to identifying information when linking records, all database research studies have unavoidable risks, such as a computer hacker breaching the computer system with the data.	○	○	
Q4.5 The unavoidable risk of a hacker breaching a research computer system is not more than the daily risk of hackers breaching computer systems with private data owned by businesses or governments.	○	○	
Q4.6 The use of the MINDFIRL software will further reduce research risks of patient matching to the minimum necessary.	○	○	
Q4.7 Do you believe that the risks of a database study (with identifiable information) are greater than “minimal risk” for **regular health data** (i.e., excluding sensitive information such as HIV, mental health, or substance abuse information).	○	○	
Q4.8 Do you believe that the risks of a database study (with identifiable information) are greater than “minimal risk” for **sensitive health data**, such as HIV, mental health or substance abuse information.	○	○	

Part 4

Part 4: Revisions to Selected FAQ, continued.

We received a lot of feedback relating to the FAQ “What difference is my data going to make?” Based on this feedback, we wrote two different versions of this FAQ.

What difference is my data going to make?

OPTION 1: Your information should help us make discoveries from this research in two ways. First, it is easier to make discoveries or find patterns if there are more records in the dataset. Second, it is important for our data to be representative of the population. If people like you are not included in the

research, then what we learn will not be useful to you or others in similar situations. In other words, without your information, our findings will not be representative of you.

OPTION 2: In short, your information could help make any discoveries from this research more helpful to people like you. In research, we use information about a group of people, called a “sample,” to understand things about a larger group or “population.” If our data is too different from the population then we cannot learn very much from the research. If people like you are not included in the research, then what we learn will not be useful to you or others like you. For example, if young adults are excluded from all studies about exercise, it will be difficult to know the types and varieties of exercise that are most beneficial to young adults. **[Suggestion for Researchers using this FAQ: Researchers should consider swapping this example with one that relates to the present research]** In other words, without your information it will be harder for us to understand how this research relates to people like you.

Please tell use which version of the FAQ you prefer.

Option 1

Option 2

A couple of participants were concerned with the use of phrase “people like you.” We are interested in your thoughts on the use of this phrase in the FAQ. (Please include any suggestions for replacing this phrase in your comments. If you have no comments, please type in N/A)

Do you have any other thoughts or comments about the FAQ above?
(If you have no comments, please type in N/A)

Part 5

<u>Part 5: Questions About the Video.</u>

In Round 1, we received a lot of feedback on the video. We updated the video based on this feedback. Please watch the video and answer the questions below.

Please rate the video.

	Very helpful	Helpful	Somewhat helpful	S h
How helpful was the updated version of the video explaining MINDFIRL?	○	○	○	
How helpful do you think this FAQ website will be to patients interested in learning about research?	○	○	○	

Please share your reason for your answer above.
(If you have no comments, please type N/A)

Part6

Part 6: Revised FAQ Wording and Format

Based on the Round 2 feedback, we revised the FAQ in two ways. First, we changed the wording of several FAQs and included many (but not all) participants' suggestions. Second, we changed the format of the FAQ document

to a website to give readers more freedom to navigate to the questions that are most relevant to them.
We would like you to spend a little time to read/explore this new FAQ and answer two questions:

1. How helpful was the new website format of the FAQ?

2. Please share any comments or suggestions for this new FAQ. Please include any question-specific feedback here. This is the last opportunity to share any suggestions for this FAQ.

Please follow this link to the updated FAQ:

Please tell us what you think about the new website.

	Very helpful	Helpful	Somewhat helpful	Slightly helpful	Not a[illegible] all helpfu[illegible]
How helpful was the new website format of the FAQ?	○	○	○	○	○

Please share any comments or suggestions for this new FAQ. Please include any question-specific feedback here. **This is the last opportunity to share any suggestions for this FAQ.**
(If you have no comments, please type N/A)

Closing

We thank you for your participation in our Delphi study.

Please select your preferred method of payment:

Amazon gift card

Target gift card

Powered by Qualtrics
