# Supplementary material for: Communicating With Patients About Software for Enhancing Privacy in Secondary Database Research Involving Record Linkage: Delphi Study
Source: J Med Internet Res. 2020 Dec 15;22(12):e20783. doi: 10.2196/20783 (PMC7772068; doi:10.2196/20783)

This is a Multimedia Appendix to a full manuscript published in the J Med Internet Res.
For full copyright and citation information see http://dx.doi.org/10.2196/jmir.20783

SUMMARY OF ROUND 3

Thirty-three (33) participants completed Round 3 of the Delphi process. Overall, most participants responded favorably to the website layout and the changes that were made based on responses to previous rounds. Below is a breakdown of your responses by section.

Part 1: Revisions to Selected FAQs

In both Round 1 and Round 2, we received feedback supporting more detail and feedback requesting that we shorten the FAQs. A number of participants thought that the FAQ is too long. We asked you to provide feedback on the inclusion of information on questions a and b listed below.

a. Who will be able to see the identifying information?

b. Who will b18e able to see the Non-identifying information or health-related study data?

Please tell us if you think that the FAQ would be better without paragraphs (a) and (b).

Yes, the FAQ is better without sections (a) and (b): N= 8

No, the FAQ is better with sections (a) and (b): N= 25

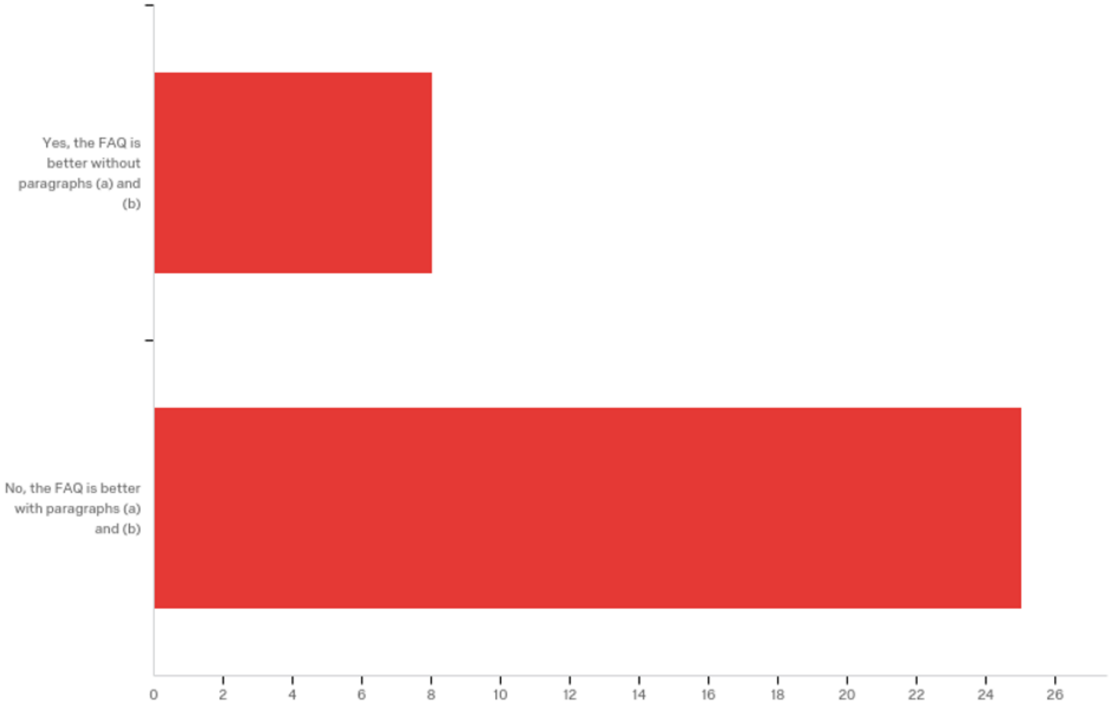

Most participants conveyed that information on who will see identifying and non-identifying information should be provided in the FAQ. The general consensus was that though the sections are lengthy, the information should still be provided for those who are interested.

With respect to the FAQ concerning how to convey comparisons of the record linkage process conventionally and using MINDFIRL, the most popular preference for conveying how well records could be matched with limited identifying information was, "The people who used the MINDFIRL prototype saw 93% less identifying information."

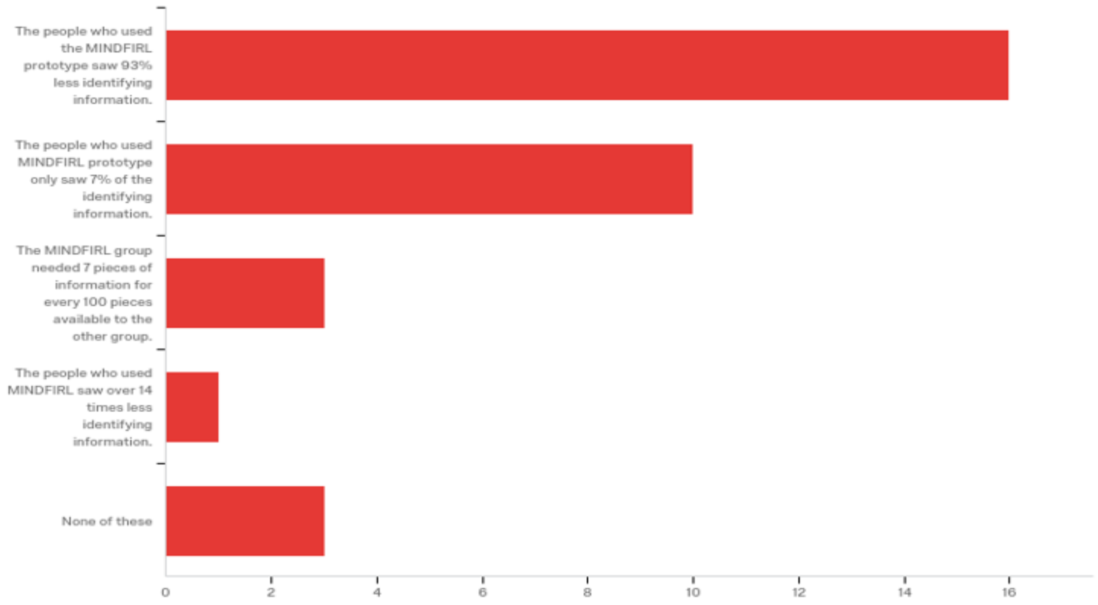

With respect to the response wording to the FAQ, "Does MINDFIRL reduce the quality of matched records?" most participants selected option B.

OPTION A: Does MINDFIRL reduce the quality of matched records?
Early evaluations of MINDFIRL show that it helps researchers make high-quality matches. People who used a prototype version of MINDFIRL were just as accurate as people who saw all of the identifying information. However, the people who used the MINDFIRL prototype saw 93% less identifying information. This means that **people using MINDFIRL were just as good at patient-matching as people who saw everyone's identifying information even though people using MINDFIRL saw far less identifying information.**

OPTION B: Does MINDFIRL reduce the quality of matched records?
No. One study showed that people who used an early version of MINDFIRL were just as accurate as people who saw 100% of the identifying information. However, the people who used MINDFIRL only saw 7% of the identifying information.

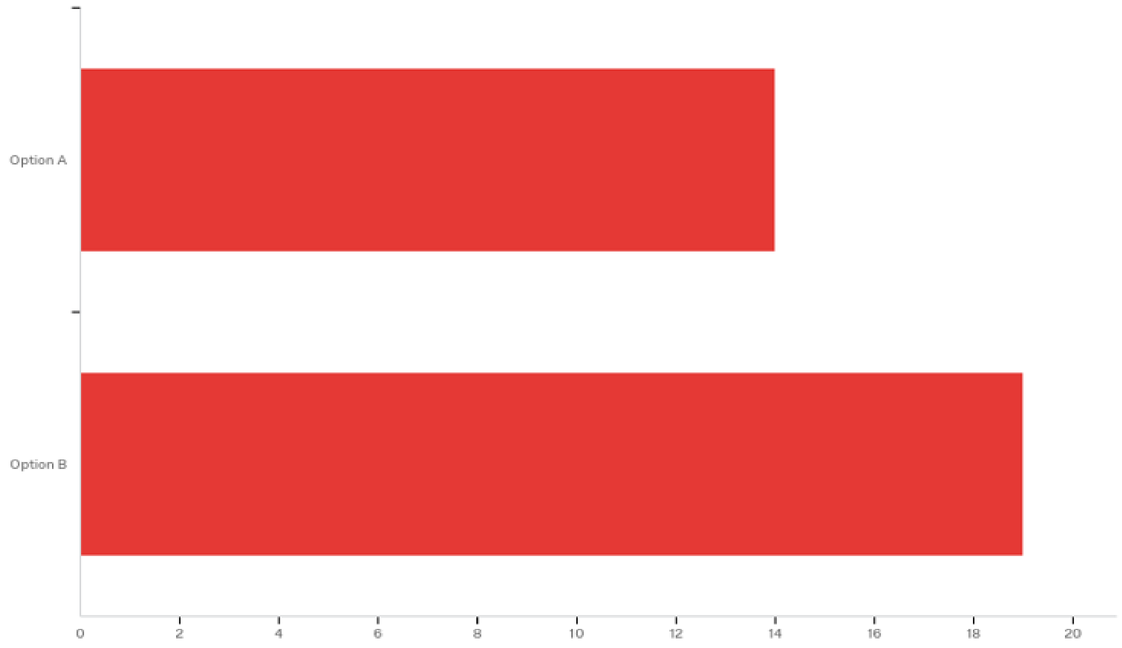

In response to FAQ, "What difference is my data going to make?" we provided to options, and most participants selected option 1.

OPTION 1: Your information should help us make discoveries from this research in two ways. First, it is easier to make discoveries or find patterns if there are more records in the dataset. Second, it is important for our data to be representative of the population. If people like you are not included in the research, then what we learn will not be useful to you or others in similar situations. In other words, without your information, our findings will not be representative of you.

OPTION 2: In short, your information could help make any discoveries from this research more helpful to people like you. In research, we use information about a group of people, called a "sample," to understand things about a larger group or "population." If our data is too different from the population then we cannot learn very much from the research. If people like you are not included in the research, then what we learn will not be useful to you or others like you. For example, if young adults are excluded from all studies about exercise, it will be difficult to know the types and varieties of exercise that are most beneficial to young adults. **[Suggestion for Researchers using this FAQ: Researchers should consider swapping this example with one that relates to the present research]** In other words, without your information it will be harder for us to understand how this research relates to people like you.

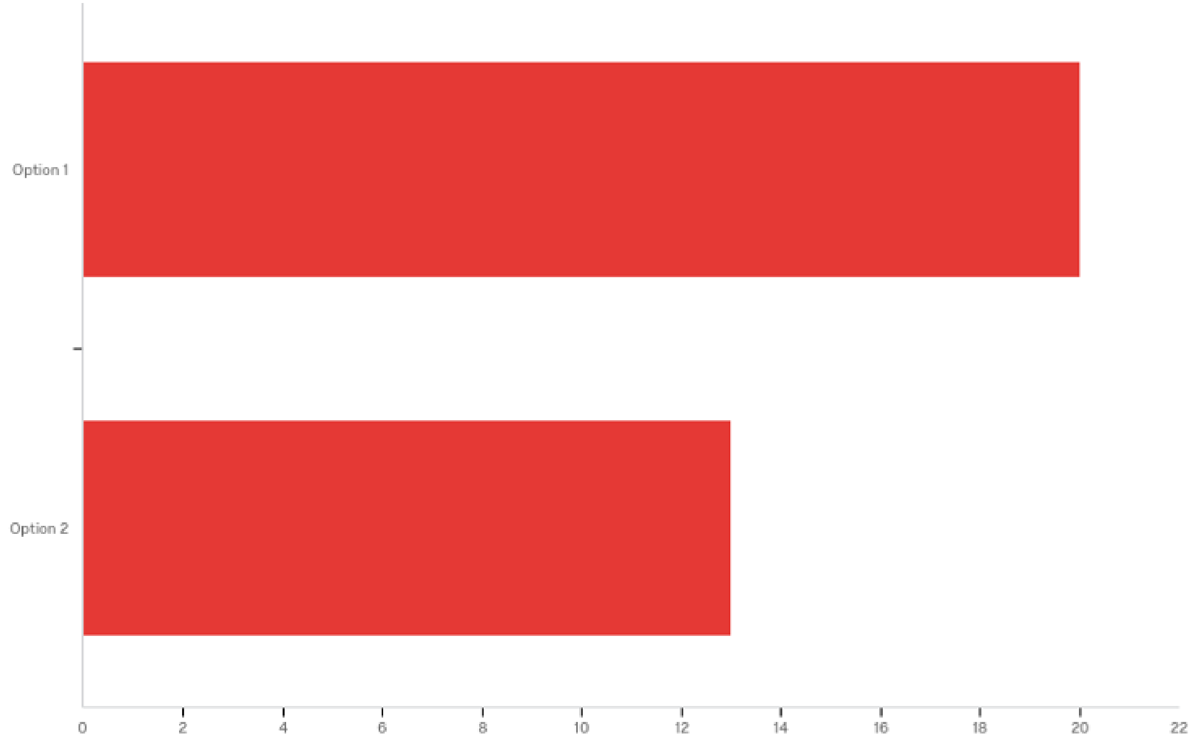

In terms of the video demonstrating MINDFIRL, most participants found it to be very helpful as shown below:

How helpful was the updated version of the video explaining MINDFIRL?

Very helpful. **N= 21**

Helpful. **N= 10**

Somewhat helpful. **N= 0**

Slightly helpful. **N= 1**

Not at all helpful. **N= 1**

When we examined your responses to the question about the potential helpfulness of the FAQ website to patients interested in research, most participants conveyed that the website will be very helpful as shown below:

How helpful do you think this FAQ website will be to patients interested in learning about research?

Very helpful. **N= 21**

Helpful. **N= 6**

Somewhat helpful. **N= 3**

Slightly helpful. **N= 1**

Not at all helpful. **N= 2**

The results concerning the video and the website layout are captured in the graph below:

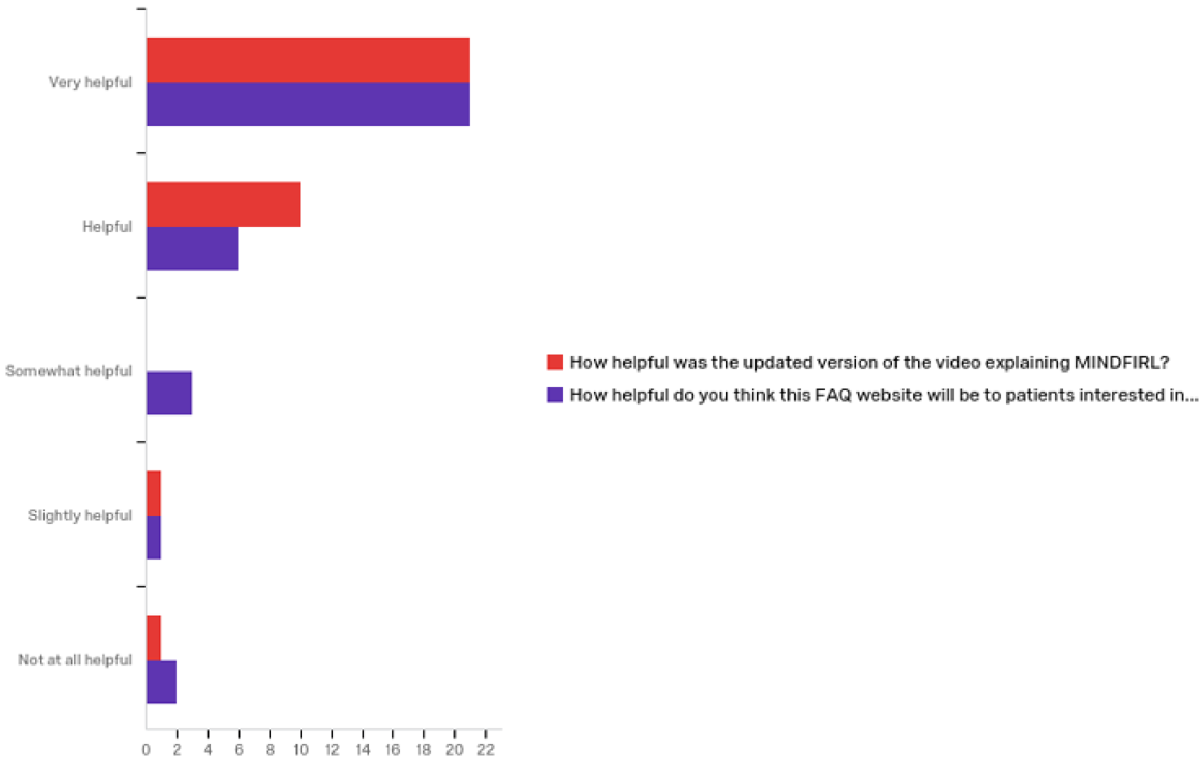

With respect to the new website format of the FAQ, most participants found the updated format to be very helpful as shown below:

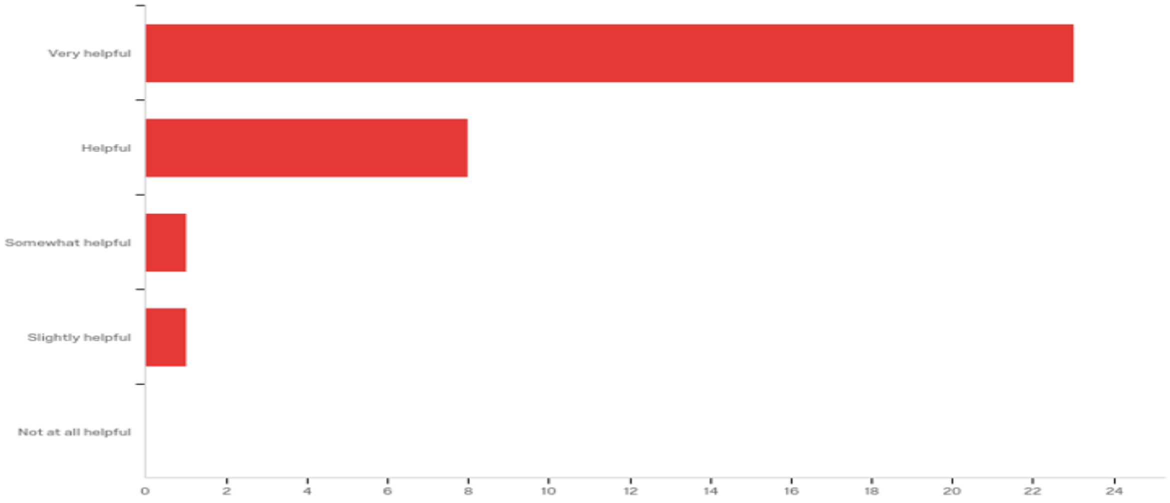

Supplement: Multimedia Appendix 6 [file jmir_v22i12e20783_app6.pdf]
