# Supplementary material for: Communicating With Patients About Software for Enhancing Privacy in Secondary Database Research Involving Record Linkage: Delphi Study
Source: J Med Internet Res. 2020 Dec 15;22(12):e20783. doi: 10.2196/20783 (PMC7772068; doi:10.2196/20783)
Supplement: Multimedia Appendix 7 [file jmir_v22i12e20783_app7.pdf]

# Frequently Asked Questions

☐ Section 1: Data and identifying information
☐ Section 2: MINDFIRL and the patient matching process
☐ Section 3: Protection and storage of my matched data
☐ Section 4: Importance and impact of using my data
☐ Section 5: Data handling after the completion of the study

## ☐ Section 1: Data and identifying information

**- 1.1 Why do you need to know who I am?**
When an organization, such as a hospital, collects information about an individual, it stores that information in a record. If the organization collects information about someone else, that information is stored in a different record. The collection of all these records is stored in a system of records called a database. Our goal is to match records from different databases. This means that, we need to know some limited information about you so we do not mismatch your records with someone else's. We refer to this limited information as 'identifying information.'

**- 1.2 What is identifying information?**
Identifying information is information that helps us tell you apart from someone else. It may include your date of birth, your gender, your marital status, your race or ethnicity, your identification number, or your name, among other things. This is the information that we use in the patient-matching process.

**- 1.3 What is non-identifying information?**
Non-identifying information is everything else in the *data*. Non-identifying information could include information such as diagnosis, medications, or blood pressure. This is the information that will help us answer our research question (see section 5.1).

**- 1.4 What pieces of information about me will the researchers see?**
We need different information for different steps of the research process. We need *identifying information* only to do patient matching (see section 2.1). Additionally, we need *non-identifying information* only when we are using your health related data to learn more about science or medicine. We will use a software program called MINDFIRL (MINimum Necessary Disclosure For Interactive Record Linkage) to keep identifying information separate from non-identifying information and **ensure that no one can access identifying information and non-identifying information together at the same time.** (for MINDFIRL, see sections 2.2 & 2.3)

In many cases, patient matching is done entirely with a linkage software, like MINDFIRL, and **a human may never see your identifying information** because the computer is automatically matching patients without human effort. For others records where the computer is not sure, researchers must manually make the matches.

*a. Who will be able to see the identifying information?*

The researchers who will be doing the record matching will have access to identifying information. Information such as your name, date of birth, marital status, and gender help distinguish you from other people. Our researchers need to access identifying information to match patient records.

We are using the MINDFIRL software (see section 2.2) to protect identifying information and prevent unnecessary privacy loss during this process. First, MINDFIRL separates identifying information from the non-identifying information. This means that, **no one can access the identifying information AND the health-related data at the same time.**

**Second, MINDFIRL tells researchers when two records have the same identifying information without showing details.** In these cases, our researchers might not need to see any specific identifying information to make a match.

**MINDFIRL also tells researcher when records are highly similar without showing details.** MINDFIRL shows identifying information only on an 'as needed' basis. For example, a researcher might want to see some details to know if a difference is

[illegible] important (e.g, to tell twins apart). This means that MINDFIRL can help catch common matching problems, such as nicknames (e.g., Pam v. Pamela) or typos (e.g., John v. Jonh), without showing the rest of your identifying information. To see how MINDFIRL works with a specific example, see section 2.3.

*b. Who will be able to see the Non-identifying information or health-related study data?*

The researchers who are doing the main research will be able to see the non-identifying, health-related data. The main research takes place after the patient-matching process and after all identifying information has been removed from the data using the MINDFIRL software. This means that, no one can access the identifying information AND the health-related data at the same time. We will code your non-identifying information to protect your identity. **This allows us to use your information to make scientific or medical discoveries without knowing which information belongs to you.**

- 1.5 If a researcher sees my name in the data when matching, how much will they know about me?

If a researcher sees your name in the *data*, they might see some more identifying information about you (e.g., birth dates, identification numbers, race, gender). But **they will not see other health details, such as your diagnosis, health status, blood pressure, etc.** Your name is an identifier. We use identifying information only during the patient-matching step of the research to help link records from different databases. In this step, other details about you, such as your health information, are not used to match records. **We only use this health-related data once we remove your identifying information to protect your privacy.**

## ☐ Section 2: MINDFIRL and the patient matching process

- 2.1 What is patient-matching?

**Patient-matching is the process of linking records of the same real-world person from different databases.** Patient matching helps researchers answer difficult questions. For example, is disease treatment A better than treatment B for keeping patients healthy? To do this, we might want to count the number of emergency room visits Jane Doe made this year across several hospitals. This requires linking records from all the hospitals she visited. After patient-matching, no identifying information is needed; it is removed from the *linked data*.

Patient-matching is challenging because a universal identification number does not exist to easily link records in different systems. Instead, **we have to use identifying information, like first and last names, birthdates, race, and gender to decide which records belong to the same person.** Below is an example of what patient-matching might look like:

*Figure 1: This image shows what patient matching looks like without MINDFIRL. In this example, the researchers can see all identifying information.*

| | ID | First name | Last name | DoB(M/D/Y) | Sex | Race |
|---|---|---|---|---|---|---|
| Patient in Database 1 → | 8000002767 | JUDE | WILLIAM | 09/09/1906 | M | W |
| Patient in Database 2 → | 8000003567 | JUDE | WILLIAM JR | 09/09/1960 | M | B |

- 2.2 What is MINDFIRL?

MINDFIRL (Minimum Necessary Disclosure for Interactive Record Linkage) is a software that we use to help protect privacy in the patient matching process of our research. It protects privacy in a few ways. First, **it separates the identifying information from the health-related study information.** That way, the researchers can view *identifying information* to help them accurately match records without seeing sensitive information.

The image below shows how MINDFIRL works. Data owners use MINDFIRL to give us their data for research. **MINDFIRL separates the identifying and *non-identifying information* immediately.** In this way, if a researcher sees identifying information

during the patient-matching process, the researcher cannot see any other sensitive information at the same time. After the patient-matching process, MINDFIRL gives us the *linked data* without any identifying information.

*Figure 2: This image shows how MINDFIRL helps the patient-matching process while limiting access to sensitive information. MINDFIRL uses encryption to separate identifying and non-identifying information during patient-matching and the main research.*

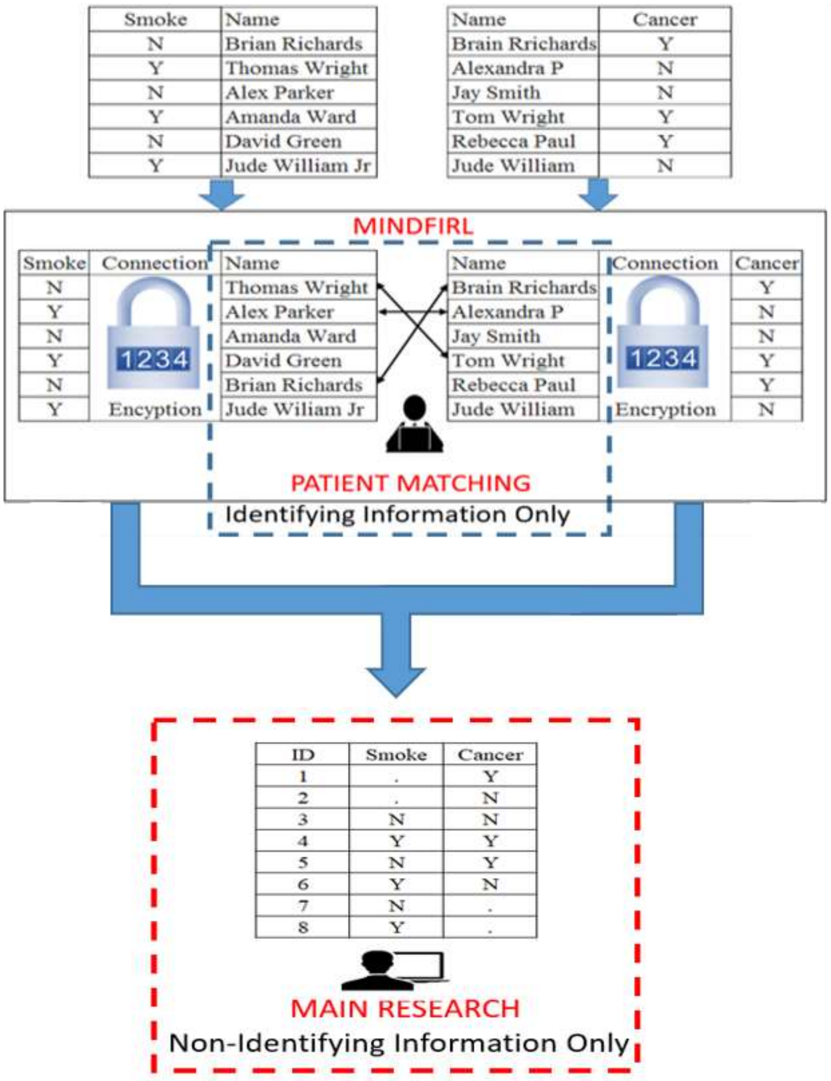


Second, MINDFIRL hides identifying information, while giving our researchers clues about how similar or different the same identifier is in two records. That way, **MINDFIRL allows our researcher to match records without seeing the specific identifying information.** See below.

*Figure 3: This image shows what researchers would see when they use MINFIRL during the patient-matching process. The symbols help researchers understand how similar or different the identifying information is without seeing every detail.*

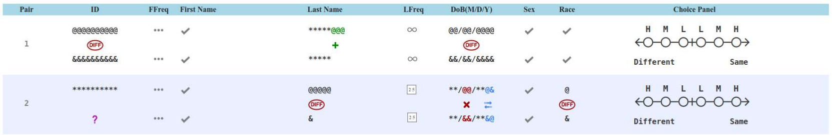


Third, MINDFIRL allows researchers to selectively reveal information to help accurately match patients. This way, **a researcher does not have to see every identifier in order to make a correct match.** You can see how this works in this clickable demo (You must use chrome on your PC for this demo to work well. The link will not work on a phone.): **Click here to try it.**

Finally, **MINDFIRL tracks and records the information that researchers see.** In this way, the research supervisors can ensure that the researchers who are matching records are not abusing their position by revealing too much.**[Suggestion for researchers using this FAQ: We encourage researchers to setup an audit and reporting process when using MINDFIRL and to be transparent about this process here.]**

- 2.3 What does MINDFIRL look like?
Click **here** to see a video of MINDFIRL and **here** to try it. (You must use chrome on your PC for this demo to work well. The link will not work on a phone).

- 2.4 What information do you need in order to match my records?
For some people, very little *identifying information* is needed to match records. For example, records from someone with a very unique name may be easily matched with just a name and perhaps their date of birth. In contrast, the records for someone with a common name may be harder for the researchers to accurately match. As a result, additional information such as gender, race or ethnicity, and identification numbers may be needed.

- 2.5 Can I be identified in the linked data?
It is highly unlikely, but possible, that you might be identified in the *linked data*. The linked data is created when the MINDFIRL software connects the non-identifying information from each database. **No *identifying information* is contained in the linked data, and no one can see the identifying information and non-identifying information at the same time.** If someone wanted to identify you in the linked data, they would have to use some other information to tell you apart from everyone in the linked data (and the rest of the world). We use encryption and secure computer systems to protect this data to reduce the risk that someone can identify you in the data.

- 2.6 Are there risks to patient matching?
Patient matching is not very risky on its own, but there are unavoidable risks to all data use just as with driving. There is always a slight chance that someone outside our research group may try to gain access to your data or that someone matching the data might misuse it.

We work hard to minimize these risks. First, we store all data on secure computers that meet legal standards to minimize the risk of someone breaking into the data. Second, each researcher on this project is trained to use the software properly and comply with the law. Third, we use the MINDFIRL software to limit access to identifying information and tracks what researchers see and do with the data. These efforts will minimize the risks of patient matching.

- 2.7 How does MINDFIRL lower the risks of patient matching?
MINDFIRL is designed to improve privacy in database studies. It limits access to identifying information. It also includes tools to promote transparency and monitor researcher activity to limit risk. For example, MINDFIRL tracks what identifying information is viewed and who viewed it. This is similar to a store that has a surveillance camera to make sure that the cashier does not take money from the cash register. This tracking tool is used to discourage the misuse of your information. It also allows for setting hard limits on how much data is used. Once this limit is reached, researchers cannot reveal more information. See figure below.

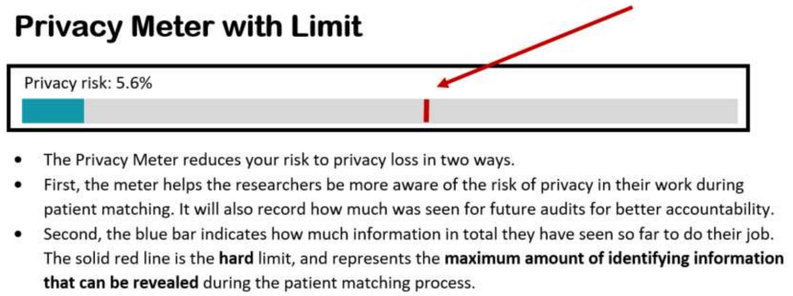

- 2.8 How does MINDFIRL enhance privacy?
MINDFIRL improves privacy by greatly reducing the identifying information viewed by researchers. MINDFIRL only shows identifying information if a researcher thinks they need it to match patient records accurately. In addition, MINDFIRL gives researchers clues to help researchers understand if two masked pieces of identifying information are the same, similar, or different. This helps researchers match records without revealing identifying **information**.

- 2.9 Does MINDFIRL reduce the quality of matched records?
No. One study showed that people who used an early version of MINDFIRL were just as accurate as people who saw 100% of the identifying information. However, the people who used the MINDFIRL prototype saw 93% less identifying information on average. See **this video** for an example.

☐ Section 3: Protection and storage of my matched data

**[Suggestion for researchers using this FAQ: The following sections contain FAQ questions that might have different answers depending on the specific research project. Researchers who use this FAQ for their projects will have to fill out the answers with information specific to their project.]**

- 3.1 Where will data about me be kept?
a. *Identifying information*

Your identifying information will be kept... **[Suggestion for researchers using this FAQ: This information will vary depending on the specific research project and protocol. Researchers should describe how they will store and protect this information.**

**One example response might be: All research data is stored in a secure server at Texas A&M University. The Texas A&M University Information Technology (IT) department maintains the servers. See the next section on how the data is secured on this server. Your identifying information will be kept in a separate secure location with different access controls (e.g., password protection). Only the staff involved with patient matching will have access to this folder.]**

b. *Non-identifying information*

Your non-identifying information such as diagnosis type, medications, etc., will be kept... **[Suggestion for researchers using this FAQ: This information will vary depending on the specific research project and protocol. Researchers should describe how they will store and protect this information.**

**One example response might be: The non-identifying information is stored in the same secured server as the identifying information. However, the non-identifying information will be stored in a different secure folder from the identifying information. Only staff conducting analysis with the data will have access to the folder with the non-identifying data.]**

- 3.2 What security measures are you using to protect me?
**[Suggestion for researchers using this FAQ: This information will vary depending on the specific research project and protocol. Researchers should describe the specific security measures for the study.**

**Some possible answers might be:**
**• The server is located in the secure facility with 24/7 monitoring**
**• Dual authentication (For example, a password and a code sent to a researcher's phone)**

**• Firewall (server is located behind a secure network with limited access from outside)**
**• Virtual Private Network (VPN) encrypted connections (connections from outside are always encrypted)**
**• No data is allowed to be taken off the server**
**• Identifying information is stored separately from non-identifying information**

**• Using MINDFIRL to reduce access to identifying information**
**• Security audits for compliance.]**

- 3.3 What are you doing to make sure that my data is being used responsibly?
This research is supervised and reviewed by the institutional review board (IRB) at **[Suggestion for researchers using this FAQ: please fill in your IRB information]**. The IRB is an organization that reviews research plans to make sure the research is legal and ethical. It contains members from the public to represent the interests of research participants. The IRB is responsible for protecting the rights, welfare and well-being of the individuals in our research. The IRB monitors this research to make sure that we are following the approved research plan. For more on IRBs **click here**.

**We are also using the MINDFIRL software for record linkage to limit access to information that can identify you.** This is part of our commitment to conducting responsible research. Furthermore, ... **[Suggestion for researchers using this FAQ: This information will vary depending on the specific research project, protocol, and institutional policies. MINDFIRL allows researchers to customize settings for transparency and accountability. Researchers should describe the specific safeguards that are in place to ensure responsible data use, including policies, MINDFIRL settings, and required trainings.]**

Additionally, **we are using secure computers and encryption during each step of the research process** to ensure that only approved researchers can access this research data safely.

- 3.4 What will you do if you discover that an unauthorized person has accessed my data or my data has been otherwise misused?
**[Suggestion for researchers using this FAQ: Researchers should consider including the following information relating to unauthorized data access or use in this section:**

**• Summaries or hyperlinks to resources describing the specific laws and legal requirements**
**• Summaries or hyperlinks to any rules or policies specific to your organization**
**• A summary of any requirements from your Institutional Review Board]**

- 3.5 Who will have access to my data?
**[Suggestion for researchers using this FAQ: This information will vary depending on the specific research project. Researchers should describe the qualifications for authorized data users and consider identifying the research team.]**

☐ Section 4: Importance and impact of using my data

- 4.1 Why is my data needed?
Your data is needed so that we can better understand ... **[Suggestion for researchers using this FAQ: This information will vary depending on the specific research project. Researchers should describe the specific research question and provide enough context for readers to understand the problem the research is trying to address. Researchers will try to help readers understand how their research contributes to addressing a specific problem. For example, "Your data is advancing our knowledge of problem A in that it helps us determine associations between X and Y." One example response might be: Your data is needed so that we can better understand who is using the emergency department (ED) at different locations,**

**how often, and why. This information is expected to better inform developing policies on ED use as well as improve operations of the ED.]**

- 4.2 What difference is my data going to make?

Your information should help us make discoveries from this research in two ways. First, it is easier to make discoveries or find patterns if there are more records in the dataset. Second, it is important for our data to be representative of the population. If persons who have a similar health history or background as you are not included in the research, then what we learn will not be useful to you or others in similar situations. In other words, without your information our findings will not be representative of you.

☐ Section 5: Data handling after the completion of the study

- 5.1 What will happen to my data after this study is completed?

**[Suggestion for researchers using this FAQ: This information will vary depending on the specific research project. Researchers should discuss their plans to destroy, store, or reuse the study data.]**

- 5.2 Will my matched data be used for other research studies?

**[Suggestion for researchers using this FAQ: This information will vary depending on the specific research project. Researchers should discuss any plans to reuse the study data.]**

- 5.3 Where can I get more information?

**[Suggestion for researchers using this FAQ: This information will vary depending on the specific research project. Researchers should provide contact information.]**

pinfo@2019 all rights reserved.
Population Informatics Lab, Texas A&M University
