# Supplementary material for: Communicating With Patients About Software for Enhancing Privacy in Secondary Database Research Involving Record Linkage: Delphi Study
Source: J Med Internet Res. 2020 Dec 15;22(12):e20783. doi: 10.2196/20783 (PMC7772068; doi:10.2196/20783)
Supplement: Multimedia Appendix 9 [file jmir_v22i12e20783_app9.docx]

This is a Multimedia Appendix to a full manuscript published in the J Med Internet Res. For full copyright and citation information see http://dx.doi.org/10.2196/jmir.20783

Preliminary Thematic Analysis from Open-Ended Questions in Delphi Rounds 1-3 with Representative Quotes from Patient Participants

| Theme | Brief Description | Representative Quote |
| --- | --- | --- |
| Simplicity and brevity | Feedback expressing a preference for short and direct explanations | *“much shorter. much clearer. overall, much better. additionally, it emphasizes the concept that MIDFIRL limits patient identifying information exposure”*  *“Option B is better mainly due to being less wordy. Less is More!”* |
| Detail and completeness | Feedback expressing a preference for complete explanations with sufficient details for clarity and understanding | *“personally more info is better. i dont mind spending time reading more.”*  *“This is an EXCELLENT use of layman's speech for some very technical information, and you need to include the paragraphs.”*  *“If people find the paragraphs too long, they will skim or skip when they have enough information. They are ‘long’ for clarity purposes, and people appreciate that the info is there if they want it.”* |
| Readability | Feedback expressing a preference for content that is easy to read and uses layman language | *“Overall this is not a document I would consider as being in layman's terms - one that a high school student could easily understand. I have a B.S. and had to reread some areas a couple of times.”*  *“The statement about samples and populations is pretty well done but it's a tricky area. I had to read the example over a couple of times to understand it.”* |
| Terminology and definitions | Feedback expressing a preference for clearly defined terms and avoiding technical jargon | *“Have to define terms for patients!!! Can't assume they have same knowledge or understanding as researchers. Be aware of high health literacy!!!”*  *“The definition is still not clear in layman terms.”*  *“The answer is too much like a textbook answer. No need to explain what a sample is, etc.”* |
| Tone | Feedback concerning the tone of explanations, e.g., conversational, not patronizing. | *“The answers feel more personal, as if someone is talking to the participant, and that goes a long way when talking about the use of their personal information.”* |
| Examples | Feedback concerning the utility of examples of key concepts | *“I think an example of the type of research questions that can be addressed through database studies and the need to link patient records helps patients understand the need and importance of the software and the topic.”* |
| Visuals | Feedback concerning the utility of graphics, video, and interactive aids | *“The images really help,”* referring to the same content as *“I think the diagrams can be omitted”*  *“This video is a great example of what the researchers are trying to accomplish, and should be included as 'mandatory viewing' for persons trying to understand the program.” referring to the same content as*  *“Video narrator was too slow. Did not sound natural. Sounds like you’re speaking to dummies.”*  *“I am not as visual as some people are, I think it is important to provide the visuals, but I do think that the interactive visuals need some work.”*  *“The legend is helpful but there are a number of items listed that also use uncommon symbols (for lay people), requiring a little more attention than a legend with fewer than 5 symbols or using more commonly recognized symbols.”* |
| Data disposition and future uses | Feedback concerning patient concerns relating to what happens to the research data at the end of the project, e.g., destruction, reuse, storage | *“How can participants know their data will be destroyed or deleted at the end of the study? What reassurances can this FAQ list offer in that regard?”*  *“You might mention if data is likely to be published and then give them the opportunity to be contacted when anything is published if they are interested.”*  *“Will I be given the opportunity to choose if I want my data used for another study?”* |
| Patient Rights | Feedback concerning the explanation of patient rights and protections. | *“FAQ 15 does not inspire much confidence – ‘we'll do what we legally have to do’ is what it seems to be saying”*  *“What are the legal guidelines for breach notification?  Are there any ramifications for the institution for breach? Are there any remediation efforts that will be undertaken?”*  *“This IS very important, as often patients truly want to be in research because they want to feel they are contributing and furthering the science and research. Explaining the details and the How's and Why's their participation is valuable is very important to them.”* |
